# FHL2 enhances ITGB1-mediated ECM remodeling and cellular stiffness to promote radioresistance in non-small cell lung cancer

**DOI:** 10.1038/s41420-025-02757-6

**Published:** 2025-10-24

**Authors:** Xiaoyu Pu, Kexin Chen, Lihua Dong, Junxuan Yi, Mingwei Wang, Xinfeng Wei, Mingqi Zhao, Mengdie Zhao, Xinyan Wang, Lijuan Ding, Shunzi Jin

**Affiliations:** 1 Jilin Provincial Key Laboratory of Radiation Oncology & Therapy, Department of Radiation Oncology & Therapy, The First Hospital of Jilin University, Changchun, China; 2 Institute of Translational Medicine, The First Hospital of Jilin University, Changchun, China; 3 National Health Commission Key Laboratory of Radiobiology, School of Public Health, Jilin University, Changchun, China

**Keywords:** Cancer therapy, Oncogenes

## Abstract

Radiotherapy is a cornerstone treatment for non-small cell lung cancer (NSCLC), but its efficacy is frequently limited by tumor-intrinsic radioresistance. Cellular stiffness and extracellular matrix (ECM) interactions are critical mechanisms underlying this resistance. The adaptor protein four-and-a-half LIM domains 2 (FHL2) has emerged as a key regulator of tumor radioresistance. This study elucidates the role of FHL2 in enhancing radioresistance in NSCLC through ECM remodeling and cellular stiffness. FHL2 was found to promote cell survival, DNA damage repair, and ECM remodeling in response to irradiation, with its interaction with integrin β1 (ITGB1) playing a pivotal role. Depletion of FHL2 significantly reduced cell survival and radioresistance in radioresistant NSCLC cell lines, while FHL2 overexpression upregulated ITGB1 expression. Notably, FHL2 depletion elicited effects comparable to ITGB1 knockdown, suggesting ITGB1 acts as a downstream effector of FHL2. Mechanistically, FHL2 enhances ITGB1-mediated ECM remodeling and cellular stiffness via FAK/MAPK signaling pathways, thereby promoting radioresistance. These findings position FHL2 as a potential biomarker and therapeutic target for overcoming radioresistance in NSCLC, offering a foundation for developing strategies to improve radiotherapy outcomes. This study underscores the critical role of FHL2/ITGB1 axis in tumor resistance mechanisms and highlights its therapeutic potential in NSCLC treatment.

## Introduction

Radiotherapy is essential for treating thoracic malignancies, but its efficacy is often limited by tumor cell resistance to ionizing radiation. Non-small cell lung cancer (NSCLC) exhibits greater radioresistance than small cell lung cancer, with 20–30% local recurrence in advanced and inoperable cases post-radiation [1]. This radioresistance arises not only from intrinsic cellular factors, genomic characteristics, and epigenetic modifications, but is also profoundly influenced by tumor hypoxia, radiation damage repair capacity, and the tumor microenvironment [2]. The crucial impact of radioresistance on treatment outcomes emphasizes the necessity for strategies to reverse this resistance to improve NSCLC management.

The extracellular matrix (ECM) constituting a major tumor component and providing essential mechanical support and microenvironment regulation, it is considered fundamental among the intrinsic resistance mechanisms [3, 4]. Tumor cells actively remodel the ECM through dysregulated mechanosensitive signaling and molecular interactions [5–7]. Reciprocally, the ECM critically drives tumor progression via alterations in macromolecular composition, enzyme remodeling, and biomechanical properties [8]. Previous studies demonstrated that cell-ECM interactions potentiate radiotherapy resistance [9, 10]. Crucially, integrin-dependent bidirectional ECM-cell signaling serves as a central hub coordinating pro-survival responses, such as enhanced DNA damage repair capacity and suppression of cell death [11, 12]. Specifically, the ECM engages with tumor cells via integrins, which are transmembrane receptors that mediate cell-ECM adhesion and activate downstream signaling cascades [13]. These cascades reorganize the actin cytoskeleton and reshape epithelial plasticity, enhance tumor cell stemness, and consequently heighten therapeutic resistance [14, 15].

In this context, adapter proteins, which facilitate these critical interactions and signaling processes, emerge as key components. Four-and-a-half LIM domains 2 (FHL2), an important adapter protein that promotes protein interactions through its LIM regions, has attracted attention for its role in tumour biology and therapy [16–18]. FHL2 has been shown to modulate various oncogenic pathways across multiple cancers. For example, FHL2 attenuates tumorigenesis in gastrointestinal stromal tumors by negatively regulating KIT signaling [19]. In lung adenocarcinoma, FHL2 activates the β-catenin/Wnt signaling pathway through its interaction with APC and TRIM63 complexes [18], while its expression in cancer-associated fibroblasts promotes metastasis and angiogenesis [20]. In pancreatic cancers, FHL2 expression is a critical determinant of both survival and radioresistance of cancer cells [10]. Recent studies have also highlighted that FHL2 contributes to the progression of esophageal squamous cell carcinoma by enhancing β-catenin nuclear translocation through pathways involving TAB182, reinforcing its involvement in tumor-associated signaling and progression [21]. Furthermore, FHL2 functions as a mechanotransducer of cellular activity in the tumor microenvironment, influencing cellular response to mechanical cues [22]. These findings highlight the multifaceted roles of FHL2 in mechanical regulation within tumors.

Although emerging evidence highlights the involvement of FHL2 in multiple cancers, its contribution to radioresistance, particularly in NSCLC, remains underexplored. This study aims to explore the molecular mechanisms of FHL2 in NSCLC radioresistance, specifically its role in enhancing ECM remodeling, mitigating DNA damage and providing a survival advantage against radiotherapy. Comprehensive molecular analysis will identify novel therapeutic targets and deepen the understanding of radioresistance mechanisms, potentially leading to more effective NSCLC treatment strategies.

## Results

### FHL2 is overexpressed in radioresistant NSCLC cell lines

**Fig. 1:**
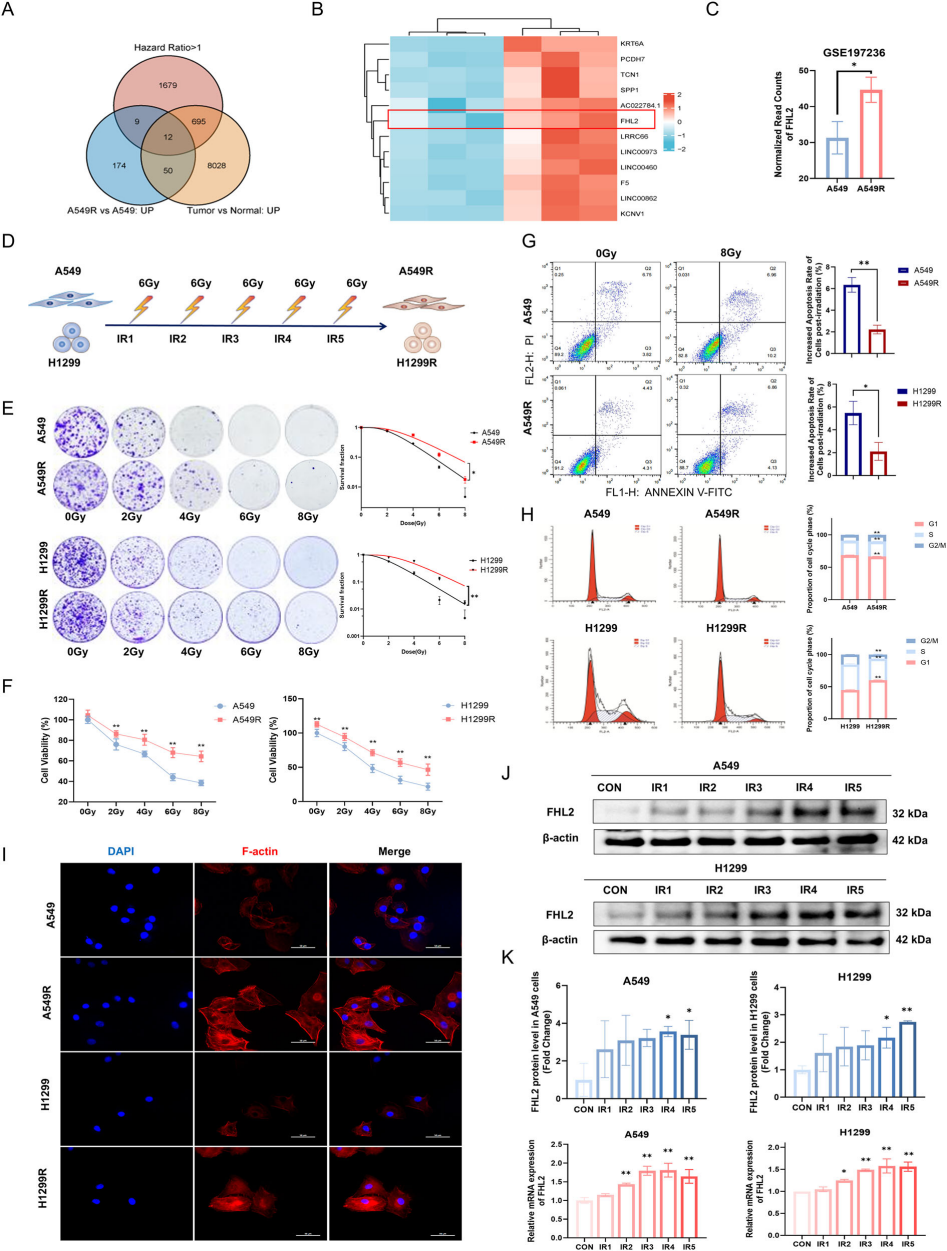
Increased cell survival and phenotypic changes in radioresistant NSCLC post-irradiation. **A** Venn diagram displaying 12 candidate genes associated with radioresistance, malignant features, and poor prognosis that were identified through analysis of GSE197236 and TCGA datasets. **B** Heatmap illustrating different expression levels of the 12 genes between NSCLC-R and parental cells using GSE197236 dataset. **C** Elevated FHL2 gene expression in A549R cell lines as evidenced by the GSE197236 dataset analysis (*n* = 3). **D** Schematic diagram of the fractionated irradiation protocol used to construct NSCLC-R models (including A549R and H1299R). **E** Clonogenic assay indicating enhanced survival of A549R and H1299R cells post-irradiation (*n* = 3). **F** The CCK-8 assay indicating enhanced survival of A549R and H1299R cells under irradiation conditions of 0, 2, 4, 6 and 8 Gy (*n* = 3). **G** Apoptosis assay showing the lower increase of radiation-induced apoptotic rate in A549R and H1299R cells (*n* = 3). **H** Cell cycle analysis demonstrating increased S phase in irradiated A549R cells and decreased G2/M phase and increased G1 phase in irradiated H1299R cells compared to their respective parental cells (*n* = 3). **I** Immunofluorescence staining revealing enhanced cytoskeletal stress fiber formation in A549R and H1299R cells compared to parental cells. Progressive elevation of **J** FHL2 protein and **K** mRNA expression through five repeated fractions of radiation (*n* = 3). Mean ± SD. *p < 0.05, **p < 0.01.

Bioinformatics analyses of radioresistant NSCLC (NSCLC-R) cell models were performed using the GSE197236 dataset combined with The Cancer Genome Atlas (TCGA) data. Venn diagram analysis revealed 12 candidate genes linked to radioresistance and poor prognosis (Fig. 1A). A heatmap displayed expression differences between NSCLC-R and parental cells (Fig. 1B), with FHL2 significantly elevated in radioresistant lines (Fig. 1C). The A549R and H1299R cell lines were constructed through repeated irradiation, culminating in a total dose of 30 Gy (Fig. 1D). Clonogenic survival and CCK-8 assays showed enhanced survival and viability in A549R and H1299R cells post-irradiation (Fig. 1E, F). Apoptosis analysis indicated a significantly lower radiation-induced apoptotic rate in A549R compared to A549, and a similar trend in H1299R versus H1299 (Fig. 1G). Additionally, the radioresistant lines exhibited altered cell cycle distributions. A549R showed an extended S phase while H1299R had a shortened G2/M phase and G1 phase arrest, indicating increased radioresistance (Fig. 1H). A549R and H1299R cells also displayed more pronounced cytoskeletal stress fibers than their parental counterparts (Fig. 1I). qPCR analysis identified FHL2 as the most significantly upregulated gene in H1299R versus parental H1299 cells (Supplementary Fig. 1A). Longitudinal analysis revealed progressive FHL2 elevation during five fractions of radiation, with notable increases in mRNA and protein levels in A549R and H1299R, highlighting its critical role in radioresistance (Fig. 1J, K).

### Elevated FHL2 expression drives radioresistance in NSCLC cell lines

**Fig. 2:**
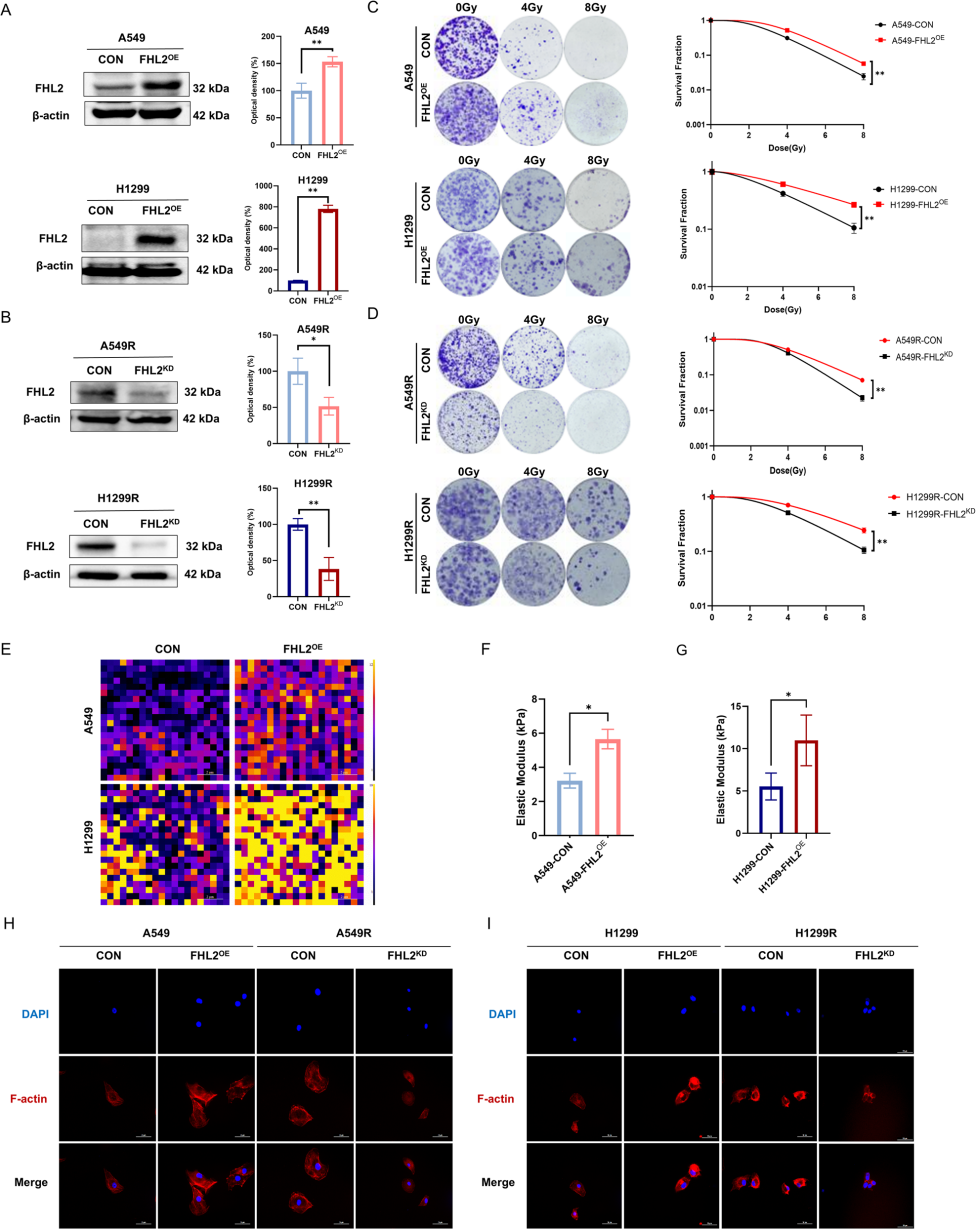
Elevated FHL2 expression in radioresistant NSCLC cell lines drives resistance. **A**, **B** Western blotting analysis demonstrating efficient FHL2 overexpression in A549 and H1299 cells and successful knockdown in A549R and H1299R cells (*n* = 3). **C**, **D** Clonogenic assay showing increased cell survival post-irradiation in A549-FHL2^OE^ and H1299-FHL2^OE^ cells compared to control cells and reduced cell survival post-irradiation in A549R and H1299R cells with FHL2 knockdown (*n* = 3). **E** Modulus heatmaps in A549 and A549-FHL2^OE^, H1299 and H1299-FHL2^OE^ cells. **F**, **G** Quantitative analysis of elastic modulus values in A549 and A549-FHL2^OE^, H1299 and H1299-FHL2^OE^ cells (*n* = 3). **H**, **I** Immunofluorescence images showing cytoskeletal changes in A549 and A549R, H1299 and H1299R, following the modulation of FHL2 expression. Mean ± SD. * *p* < 0.05, ** *p* < 0.01.

We successfully established radioresistant cell lines A549R and H1299R from their parental counterparts and examined the time-dependent alterations in FHL2 protein and mRNA expression post-irradiation. As shown in Supplementary Fig. 1B, C, FHL2 expression progressively increased in parental cells post-irradiation, while resistant models consistently exhibited higher levels and further increases in FHL2 expression. Overexpression and knockdown experiments were conducted to investigate the functional impact of FHL2. Western blotting analysis confirmed efficient overexpression of FHL2 in A549 and H1299 cells and successful knockdown in A549R and H1299R cells (Fig. 2A, B). Clonogenic assays demonstrated that FHL2 overexpression significantly enhanced survival rates post-irradiation in A549 and H1299 cells compared to controls (Fig. 2C). Conversely, knocking down FHL2 in radioresistant A549R and H1299R cell lines resulted in decreased survival post-irradiation (Fig. 2D). Correspondingly, CCK-8 assay results confirmed these trends (Supplementary Fig. 2A–D). By knocking down FHL2 in A549 or H1299 cells, we found that the post-irradiation survival fraction of the cells decreased further (Supplementary Fig. 2E). These results demonstrate that FHL2 is critical for radioresistance in NSCLC cells, as elevated expression enhances survival, while reduced expression compromises resistance.

### FHL2 enhances cytoskeletal structure and cellular stiffness in tumor cells

To explore the critical role of FHL2 in regulating tumor cell properties mechanics, its impact on cytoskeletal structure and cellular stiffness was examined in NSCLC cells. AFM was utilized to assess the apparent elastic modulus of FHL2-overexpressing NSCLC cells in comparison to that of their parental cells. The results showed a positive association between elevated FHL2 expression and increased cellular stiffness. The modulus heatmaps and quantitative analysis demonstrated that FHL2 overexpression exhibited higher elastic modulus values in A549 and H1299 cells, indicating a more rigid cellular architecture (Fig. 2E–G). FHL2 overexpression in A549 and H1299 cells led to a diffuse distribution and increased bundling of cytoskeletal stress fibers, while FHL2 knockdown reversed these effects in A549R and H1299R cells (Fig. 2H, I). These findings highlight the pivotal role of FHL2 in modulating the mechanical properties of tumor cells, which may impact their invasive and metastatic behaviors.

### Crucial function of FHL2 in modulating EMT phenotype and ECM deposition in NSCLC

**Fig. 3:**
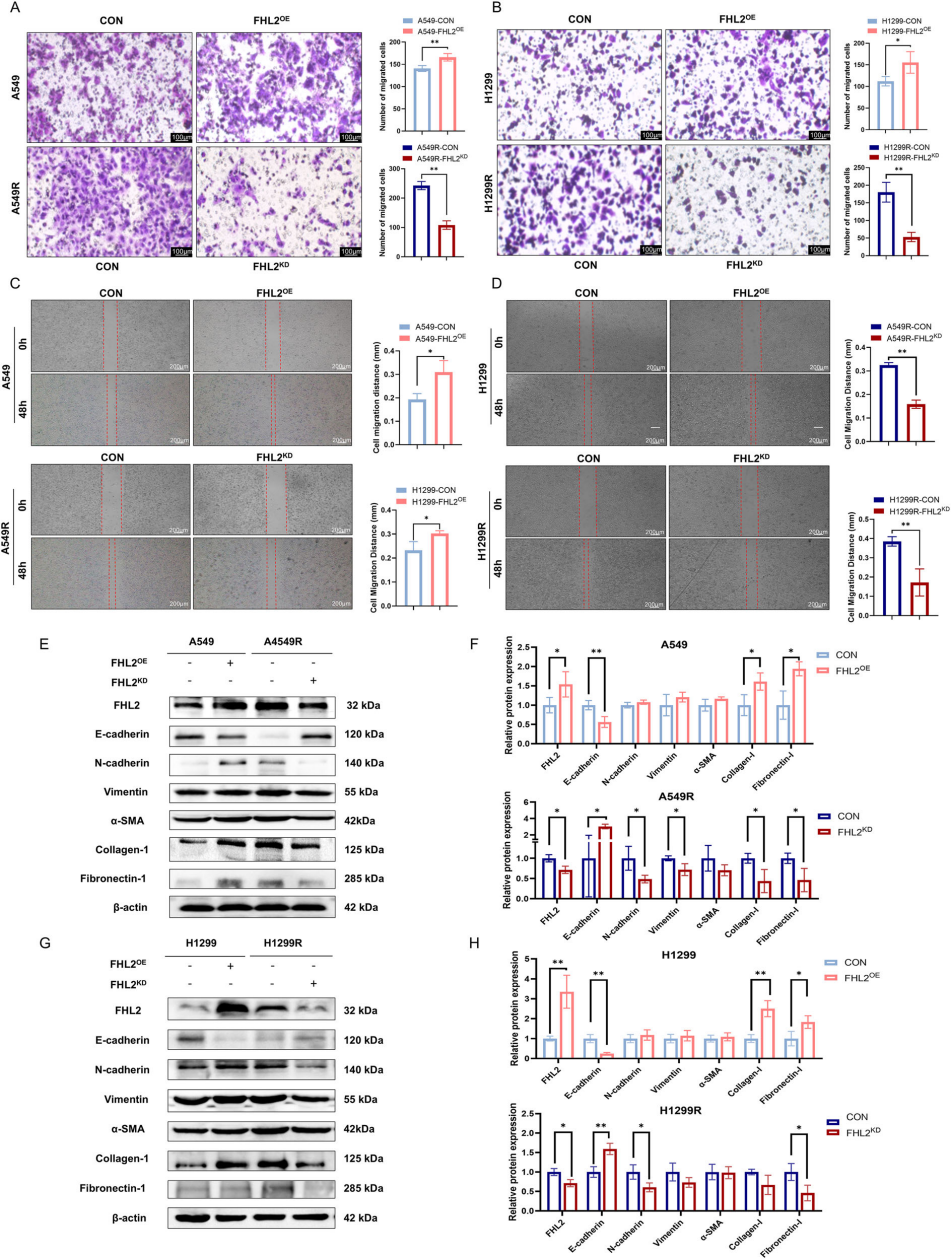
FHL2 promotes ECM deposition and EMT phenotype in NSCLC cells. **A**, **B** Transwell assay and quantitative analysis after overexpression of FHL2 in A549 and H1299 cells, and knockdown of FHL2 in A549R and H1299R cells (*n* = 3). **C**, **D** Wound healing assay and quantitative analysis after overexpression of FHL2 in A549 and H1299 cells, and knockdown of FHL2 in A549R and H1299R cells (*n* = 3). **E**, **F** Western blotting and quantitative analysis of FHL2, E-cadherin, N-cadherin, vimentin, α-SMA, collagen-1, and fibronectin-1 expression in A549 with FHL2 overexpression, and in A549R with FHL2 knockdown (*n* = 3). **G**, **H** Western blotting and quantitative analysis of FHL2, E-cadherin, N-cadherin, vimentin, α-SMA, collagen-1, and fibronectin-1 expression in H1299 with FHL2 overexpression, and in H1299R with FHL2 knockdown (*n* = 3). Mean ± SD. *p < 0.05, **p < 0.01.

Next, experiments were conducted to examine the impact of FHL2 expression on EMT phenotype and ECM deposition in NSCLC. Transwell migration and wound healing assays revealed that FHL2 enhanced the migratory capabilities of A549 and H1299 cells, while FHL2 knockdown in radioresistant A549R and H1299R cells significantly reduced their migratory capacities (Fig. 3A–D). FHL2 regulation also affected the expression of EMT- and ECM-related proteins in both NSCLC lines and their radioresistant variants (Fig. 3E–H). Specifically, FHL2 overexpression in A549 cells decreased E-cadherin and upregulated collagen-I and fibronectin-I, with a non-significant trend toward increased N-cadherin, vimentin, and α-SMA. Conversely, FHL2 knockdown in A549R cells upregulated E-cadherin and downregulated N-cadherin, vimentin, collagen-I, and fibronectin-I. In H1299 cells, FHL2 overexpression similarly downregulated E-cadherin and upregulated collagen-I and fibronectin-I, while knockdown in H1299R led to increased E-cadherin and decreased N-cadherin and fibronectin-I.

Furthermore, heatmaps derived from NSCLC data analysis within the TCGA database represented the correlations between FHL2 and various genes implicated in the ECM composition, EMT progression, and cell-matrix interactions pathways (Supplementary Fig. 3A). A positive correlation was observed between FHL2 gene expression and EMT markers (Cor = 0.39, *P* < 0.001), as well as the collagen formation pathway (Cor = 0.40, *P* < 0.001), indicating that higher gene expression correlated with increased pathway activity (Supplementary Fig. 3B, C). These findings highlight the pivotal role of FHL2 in modulating tumor biology by modulating EMT- and ECM-related pathways in NSCLC.

### FHL2 overexpression activates ITGB1-mediated FAK/MAPK signaling pathway in NSCLC

**Fig. 4:**
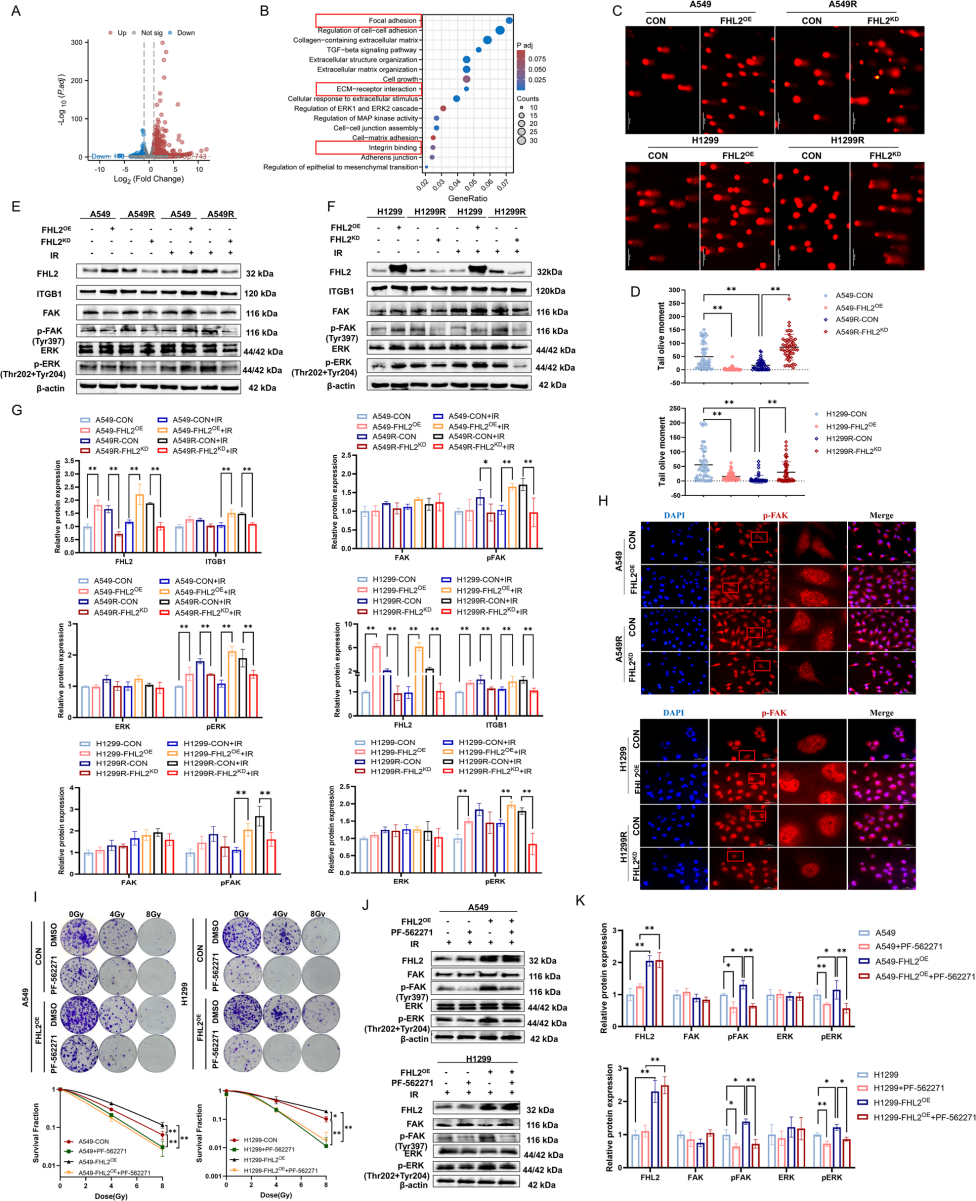
FHL2 facilitates ITGB1-mediated ECM remodeling and activates FAK/MAPK signaling. **A** Volcano plot showing differentially expressed genes between H1299-FHL2^OE^ and parental H1299 cells based on transcriptome sequencing. **B** Gene Ontology (GO) enrichment analysis of differentially expressed genes in parental H1299 and H1299-FHL2^OE^ cells. **C**, **D** Representative images and quantitative analysis of Comet assays after overexpression of FHL2 in A549 and H1299 cells, and knockdown of FHL2 in A549R and H1299R cells (*n* = 50, cell count). **E**–**G** Western blotting and quantitative analysis of the expression of FHL2 and key proteins in the FAK/MAPK pathway after overexpression of FHL2 in A549 and H1299 cells, and knockdown of FHL2 in A549R and H1299R cells under 0-Gy and 8 Gy irradiation conditions (*n* = 3). **H** Representative images of Immunofluorescence showing the phosphorylation of FAK in A549 and H1299 cells with FHL2 overexpression, and in A549R and H1299R cells with FHL2 knockdown. **I** Clonogenic assay demonstrating the FAK inhibitor PF-562271 decreased survival rates of irradiated A549 and H1299 cells including those overexpressing FHL2 (*n* = 3). **J**, **K** Western blotting and quantitative analysis of FHL2 and key proteins in the FAK/MAPK pathway after FHL2 overexpression and PF-562271 treatment in A549 and H1299 cells (*n* = 3). Mean ± SD. *p < 0.05, **p < 0.01.

Furthermore, RNA sequencing was employed to investigate the genes with varying expression levels in H1299 and H1299-FHL2^OE^ cells. Consequently, we discovered 913 genes that exhibited significant differences with an FDR of <0.05 and fold change of >1. Of these, 743 genes were upregulated and 170 genes were downregulated in H1299-FHL2^OE^ cells. A volcano plot was utilized to highlights major shifts in gene expression, emphasizing alterations in key regulatory genes (Fig. 4A). Gene Ontology (GO) enrichment analysis further confirmed the significant enrichment of these differentially expressed genes in pathways pertinent to focal adhesion, ECM-receptor interaction, and the MAPK signaling pathway (Fig. 4B). These ECM interactions and focal adhesion signaling are crucial for modulating tumor radiosensitivity [23]. The activation of these pathways significantly enhances the mechanisms of cellular damage repair and survival. Consistently, comet assays showed that FHL2 overexpression improved DNA repair in A549 and H1299 cells, while knockdown reduced this capacity in radioresistant models (Fig. 4C, D). Specifically, FAK activation via integrin-mediated adhesion to the ECM activates the downstream MAPK signaling pathway, promoting tumor aggressiveness and radiotherapy resistance.

We then investigated the impact of FHL2 on ITGB1 expression and the FAK/MAPK signaling pathway activation (Fig. 4E, F). FHL2 overexpression in A549 and H1299 cells significantly increased ITGB1 protein levels and the phosphorylation of FAK (Tyr397) and ERK (Thr202+Tyr204) after irradiation (Fig. 4G). Conversely, FHL2 knockdown in A549R and H1299R cells reduced pFAK and pERK levels, especially post-irradiation. Immunofluorescence analysis revealed that FHL2 upregulation correlated with enhanced FAK phosphorylation and focal adhesion aggregation (Fig. 4H). Meanwhile, FHL2 downregulation in radioresistant cell models was accompanied by a decrease in phosphorylated FAK level, although to a lesser extent than the increase from FHL2 upregulation. To further elucidate the role of FAK in this process, we utilized PF-562271, a selective inhibitor of FAK activation, at a concentration of 5 µM. Colony formation assays and CCK-8 assays demonstrated that inhibiting FAK activation countered the increased post-radiation cell survival from FHL2 overexpression (Fig. 4I and Supplementary Fig. 4A, B), and resulted in reduced phosphorylation levels of FAK and ERK (Fig. 4J, K). Thus, inhibiting FAK reversed FHL2-mediated radioresistance and suppressed downstream MAPK activation. These findings suggest that FHL2 regulates the FAK/MAPK signaling pathway in NSCLC radioresistance.

### FHL2 interacts with ITGB1 and promotes its stabilization in NSCLC

**Fig. 5:**
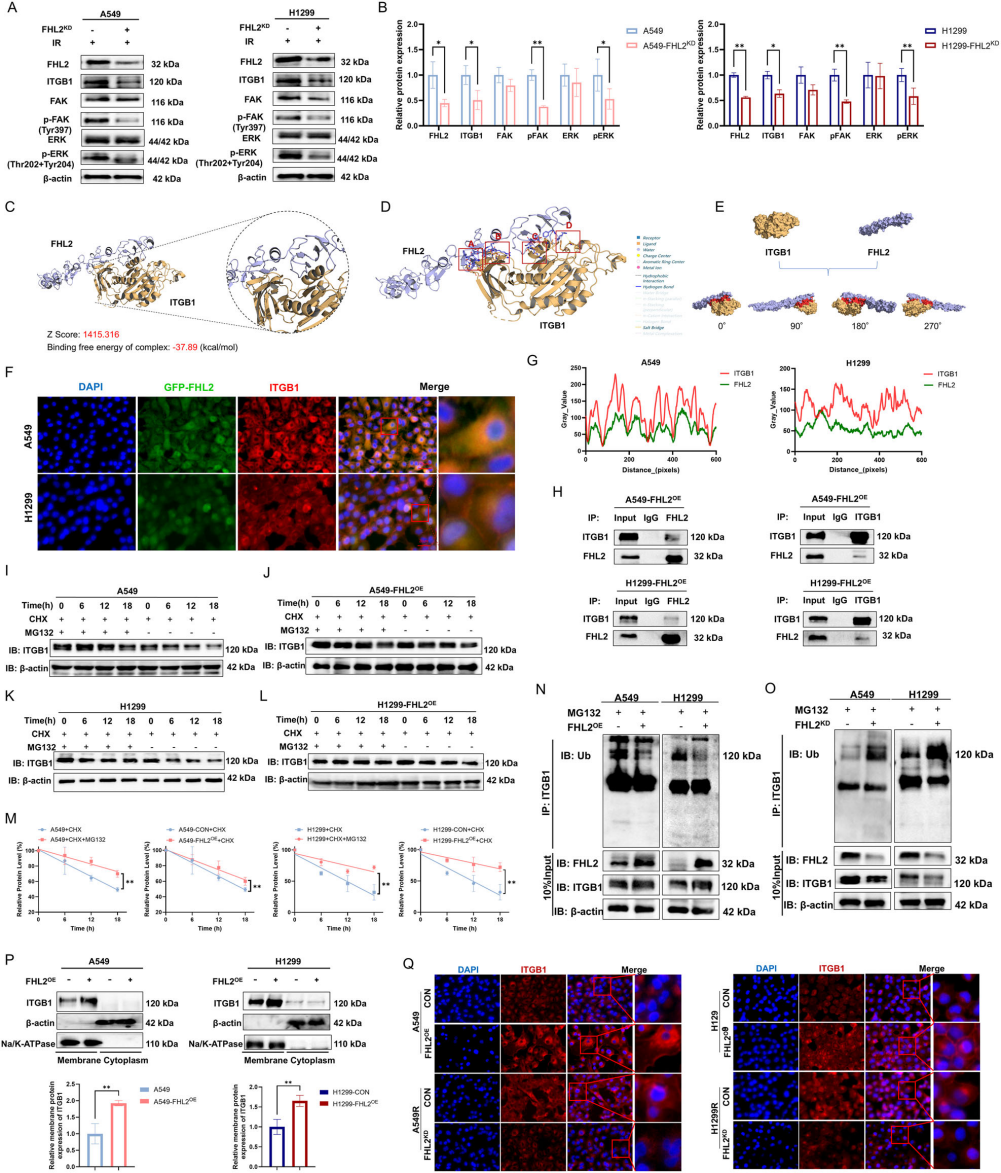
Co-localization and Interaction of FHL2 and ITGB1 in NSCLC. **A**, **B** Western blotting and quantitative analysis of the expression of FHL2, ITGB1 and key proteins in the FAK/MAPK pathway after knockdown of FHL2 in A549 and H1299 cells (*n* = 3). **C** Protein-protein docking analysis of ITGB1 and FHL2 structures from the Uniprot database showing the highest-scoring complex with a docking score of 1415.316 and a binding free energy of −37.89 kcal/mol. **D** PLIP analysis identifying multiple hydrophobic interactions, eight hydrogen bonds and one salt bridge between ITGB1 and FHL2, indicating high stability and specificity of their interaction. **E** Visualization of docking results in Surface representation. **F**, **G** Immunofluorescence images demonstrating significant co-localization of FHL2 and ITGB1 proteins in A549 and H1299 cells. **H** Co-immunoprecipitation assay confirming the physical interaction between FHL2 and ITGB1 proteins. **I**–**M** Western blotting and quantitative analysis of ITGB1 protein levels in A549, A549-FHL2^OE^, H1299, and H1299-FHL2^OE^ cells at 0, 6, 12 and 18 h, with or without MG132 treatment to inhibit proteasomal activity (*n* = 3). **N**, **O** Co-immunoprecipitation assay reveals ITGB1 ubiquitination levels in A549 and H1299 cells following FHL2 overexpression and knockdown. **P** Membrane protein extraction and Western blot validation of ITGB1 localization and expression (*n* = 3). **Q** Immunofluorescence assay was performed to observe the localization and expression of ITGB1 in A549 and H1299 cells following FHL2 overexpression (*n* = 3). Mean ± SD. *p < 0.05, **p < 0.01.

Regarding the mechanism of FHL2 regulation of ITGB1, PCR analysis revealed that FHL2 has no significant effect on ITGB1 transcription (Supplementary Fig. 5A, B). However, FHL2 knockdown in A549 or H1299 cells led to a marked reduction in ITGB1 protein levels, suggesting that FHL2 regulates ITGB1 post-transcriptionally by modulating its degradation (Fig. 5A, B). Further investigation using interaction predictions from the GeneMANIA database (Supplementary Fig. 6) and protein-protein docking analysis via ZDOCK showed a top docking score of 1415.316 and a binding free energy of −37.89 kcal/mol (Fig. 5C). Visualizations revealed multiple hydrophobic interactions, eight hydrogen bonds, and one salt bridge between ITGB1 and FHL2, suggesting a stable and specific interaction (Fig. 5D, E). Immunofluorescence assays revealed significant co-localization of FHL2 and ITGB1 in A549 and H1299 cells (Fig. 5F, G). Pearson correlation coefficients quantifying the association between FHL2 and ITGB1 reached 0.91 in A549 and 0.78 in H1299 cells, confirming significant colocalization (Supplementary Fig. 7A, B). This interaction was further validated by co-immunoprecipitation assays revealing direct physical binding (Fig. 5H).

These findings highlight their co-regulatory roles in tumor biology and suggest a collaborative function in ECM remodeling. Furthermore, we observed that the addition of the proteasome inhibitor MG132 significantly inhibited ITGB1 degradation in both cell lines, while FHL2 overexpression stabilized ITGB1 by reducing its proteasomal degradation and decreasing its ubiquitination levels (Fig. 5I–N). Conversely, the absence of FHL2 led to an increase in the ubiquitination of ITGB1 (Fig. 5O). The separate extraction of membrane and cytosolic proteins revealed differences in ITGB1 levels, indicating that FHL2 enhances the localization of ITGB1 on the cell membrane (Fig. 5P), thereby further confirming the stabilizing effect of FHL2 on ITGB1 protein. This conclusion is further supported by the immunofluorescence results (Fig. 5Q).

### ITGB1 knockdown reverses FHL2-driven radioresistance and FAK/MAPK signaling activation in NSCLC

**Fig. 6:**
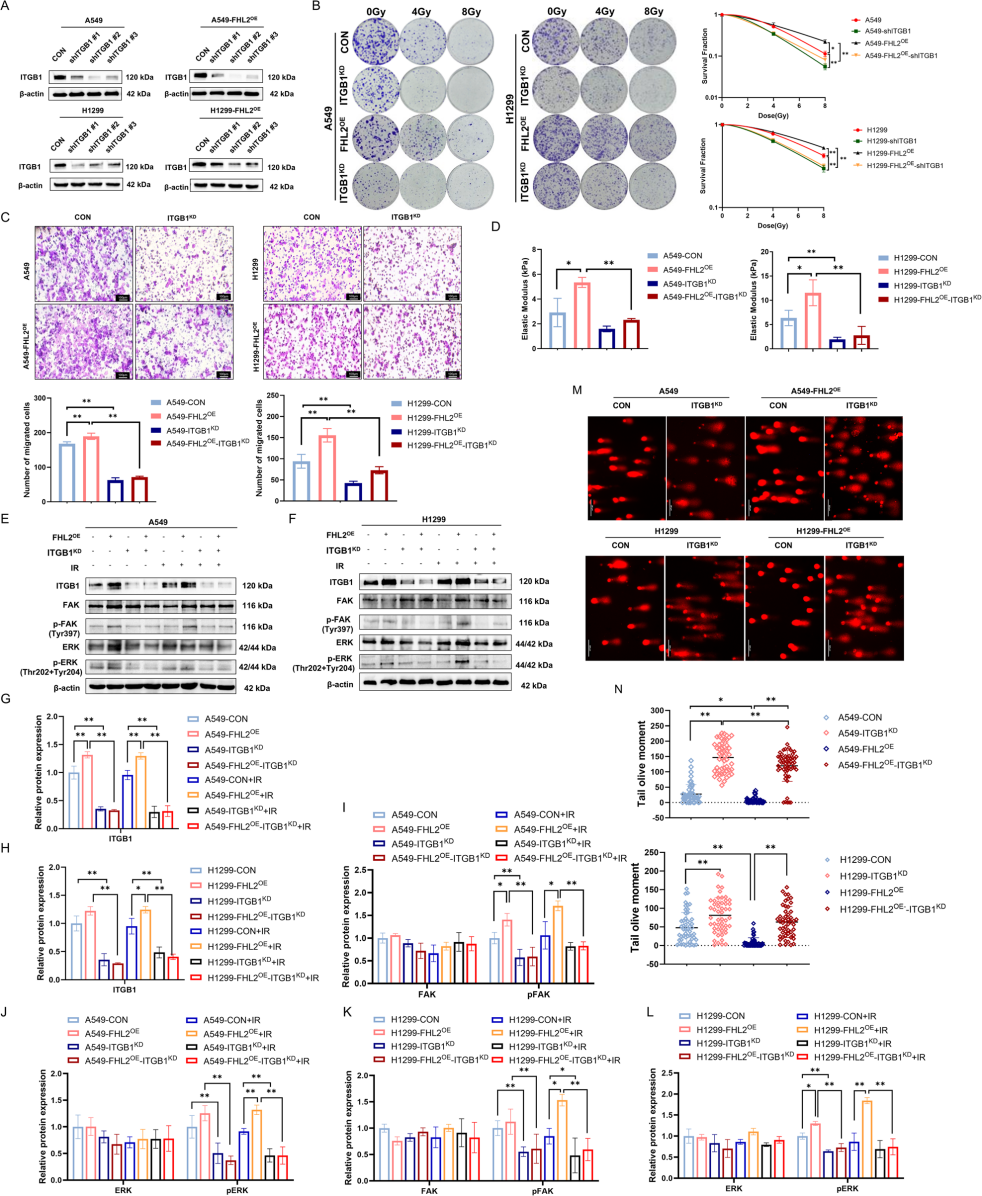
ITGB1 Knockdown Attenuates FHL2-Induced Radioresistance and FAK/MAPK Signaling Activation in Tumor Cells. **A** Stable ITGB1 expression downregulation by three different shRNAs was confirmed by western blotting in A549, A549-FHL2^OE^, H1299, and H1299-FHL2^OE^ cells. **B** Clonogenic assay demonstrating ITGB1 knockdown markedly reverses FHL2-induced radioresistance in NSCLC (*n* = 3). **C** Transwell assay showing reduced migratory capabilities in A549, A549-FHL2^OE^, H1299, and H1299-FHL2^OE^ cells following ITGB1 knockdown (*n* = 3). **D** Elastic modulus test indicating lower cell stiffness in ITGB1 knockdown cells compared to A549, A549-FHL2^OE^ and H1299, H1299-FHL2^OE^ cells (*n* = 3). **E**, **F** Western blotting results showed FAK/ERK pathway protein expression levels in ITGB1 knockdown A549 and H1299 cells under 0-Gy and 8 Gy irradiation conditions, including FHL2-overexpressing variants. **G**–**L** Quantitative analysis of the expression of the key proteins in the FAK/MAPK pathway after knockdown of ITGB1 in A549, A549-FHL2^OE^, H1299, and H1299-FHL2^OE^ cells under 0-Gy and 8 Gy irradiation conditions (*n* = 3). **M**, **N** Representative images and quantitative analysis of Comet assays after overexpression of FHL2 in A549 and H1299 cells with ITGB1 knockdown (*n* = 50, cell count). Mean ± SD. *p < 0.05, **p < 0.01.

Building on the interactions between ITGB1 and FHL2, we further explored the functional consequences of modulating ITGB1 levels. Effective ITGB1 knockdown was achieved using three different shRNAs in various NSCLC cell lines (Fig. 6A). Clonogenic and CCK-8 assays confirmed that ITGB1 knockdown significantly decreased the survival rates of irradiated A549 and H1299 cells, reversing FHL2-associated radioresistance (Fig. 6B, Supplementary Fig. 8A–D). Transwell migration assays showed reduced migratory capabilities in all four cell groups after ITGB1 knockdown (Fig. 6C). Additionally, the elastic modulus test revealed that ITGB1 knockdown decreased cell stiffness, counteracting the FHL2-induced increase (Fig. 6D). Western blotting demonstrated lower phosphorylated FAK and ERK levels in ITGB1 knockdown cells compared to their FHL2-overexpressing counterparts (Fig. 6E–L). Comet assays further confirmed the reduced DNA repair capacity in both parental and FHL2-overexpressing models following ITGB1 knockdown (Fig. 6M, N).

### FHL2 enhances radioresistance in NSCLC mouse models and is associated with adverse prognosis

**Fig. 7:**
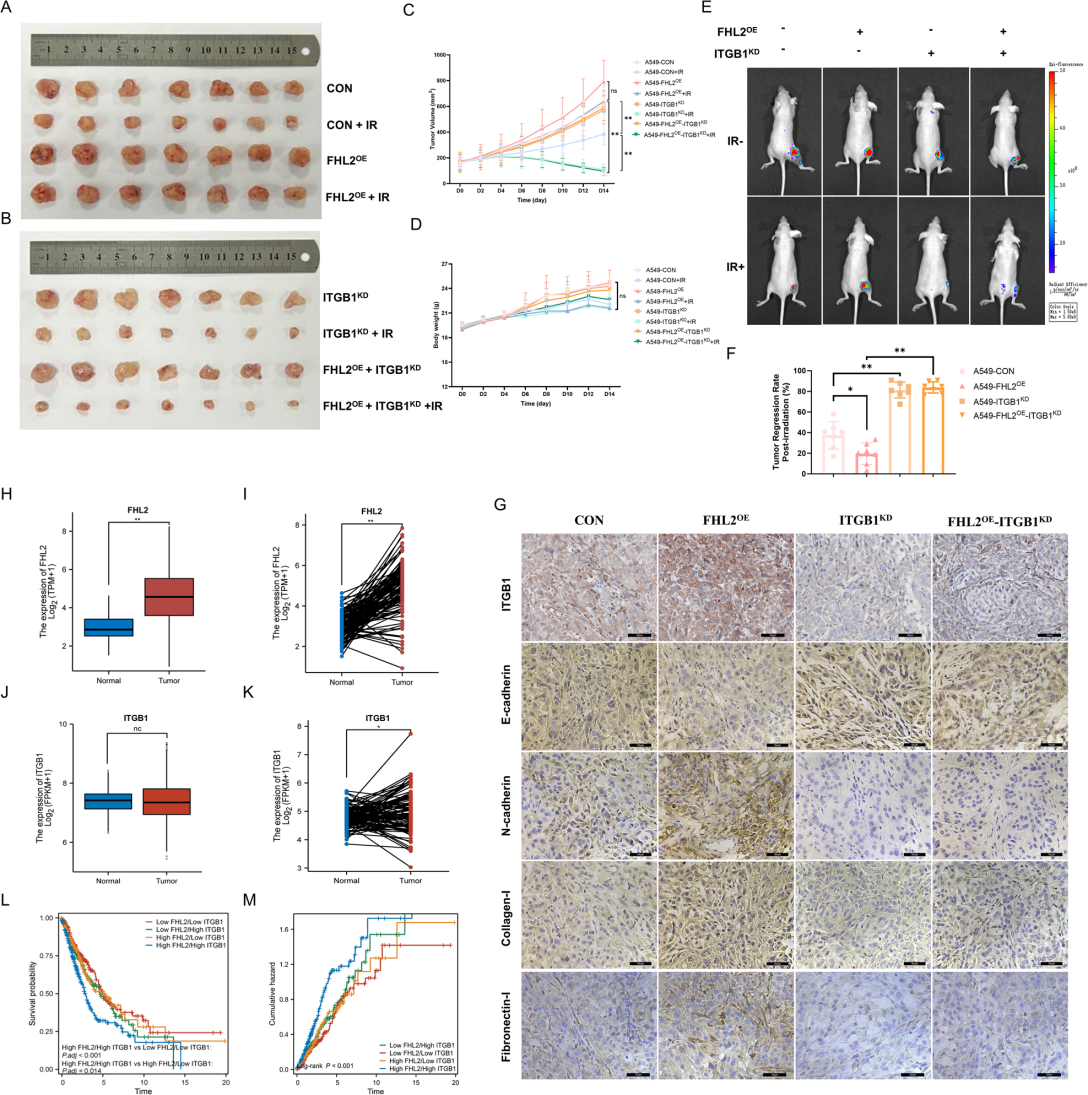
Influence of FHL2 and ITGB1 on Tumor Growth, Radioresistance, and Prognostic Outcomes in NSCLC. **A**, **B** Representative images of tumors from CDX Mouse Models bearing A549, A549-FHL2^OE^, A549-ITGB1^KD^, and A549-FHL2^OE^-ITGB1^KD^ cells, with and without irradiation (*n* = 7). **C** Tumor volume and **D** Body weight changes following tumor irradiation in different CDX model groups (*n* = 7). **E** Representative bioluminescence imaging and **F** quantification of post-irradiation tumor regression across different groups (*n* = 7). **G** Representative immunohistochemical staining images of ITGB1, E-cadherin, N-cadherin, collagen I, and fibronectin I in tumor tissues of CDX models. **H**–**K** mRNA expression levels of FHL2 and ITGB1 in paired and unpaired lung cancer samples compared to normal lung tissues, sourced from TCGA and GTEx databases. **L** Prognostic co-analysis of FHL2 and ITGB1 expression levels in NSCLC patients. **M** Cumulative hazard associated with different FHL2 and ITGB1 expression combinations. Mean ± SD. *p < 0.05, **p < 0.01, ns not significant.

FHL2 overexpression in A549 cells within cell-derived xenograft (CDX) models significantly increased tumor volume and radioresistance, as evidenced by progressive tumor growth over 14 days post-radiation (Fig. 7A). Conversely, ITGB1 knockdown in both parental and FHL2-overexpressing CDX models resulted in a noticeable reduction in tumor volume and effectively reversed the enhanced FHL2-mediated radioresistance (Fig. 7B, C). Body weight measurements across different experimental groups post-irradiation showed no significant changes (Fig. 7D). Bioluminescence imaging confirmed these effects, demonstrating distinct tumor responses to FHL2 overexpression and ITGB1 knockdown (Fig. 7E). Significant differences in tumor regression rates across CDX model groups, highlighting the critical roles of FHL2 and ITGB1 in tumor growth and radiotherapy response (Fig. 7F). Immunohistochemistry results revealed that FHL2 overexpression upregulated ITGB1 and mesenchymal markers while downregulating E-cadherin, enhancing tumor aggressiveness and radioresistance in vivo (Fig. 7G).

Clinically, elevated FHL2 and ITGB1 mRNA levels were consistently found in lung cancer samples relative to normal tissues across both paired and unpaired samples in the TCGA database (Fig. 7H–K), underscoring their critical roles in NSCLC pathogenesis. Co-expression analysis indicated that FHL2 and ITGB1 levels impact survival outcomes in NSCLC, with higher expression correlating to poorer prognoses (Fig. 7L, M). Additionally, the ROC curve analysis using TCGA-NSCLC and GTEx-normal cohorts (Supplementary Fig. 9A–C) indicates that FHL2 and ITGB1 have potential diagnostic value and serve as references for diagnosis.

## Discussion

**Fig. 8:**
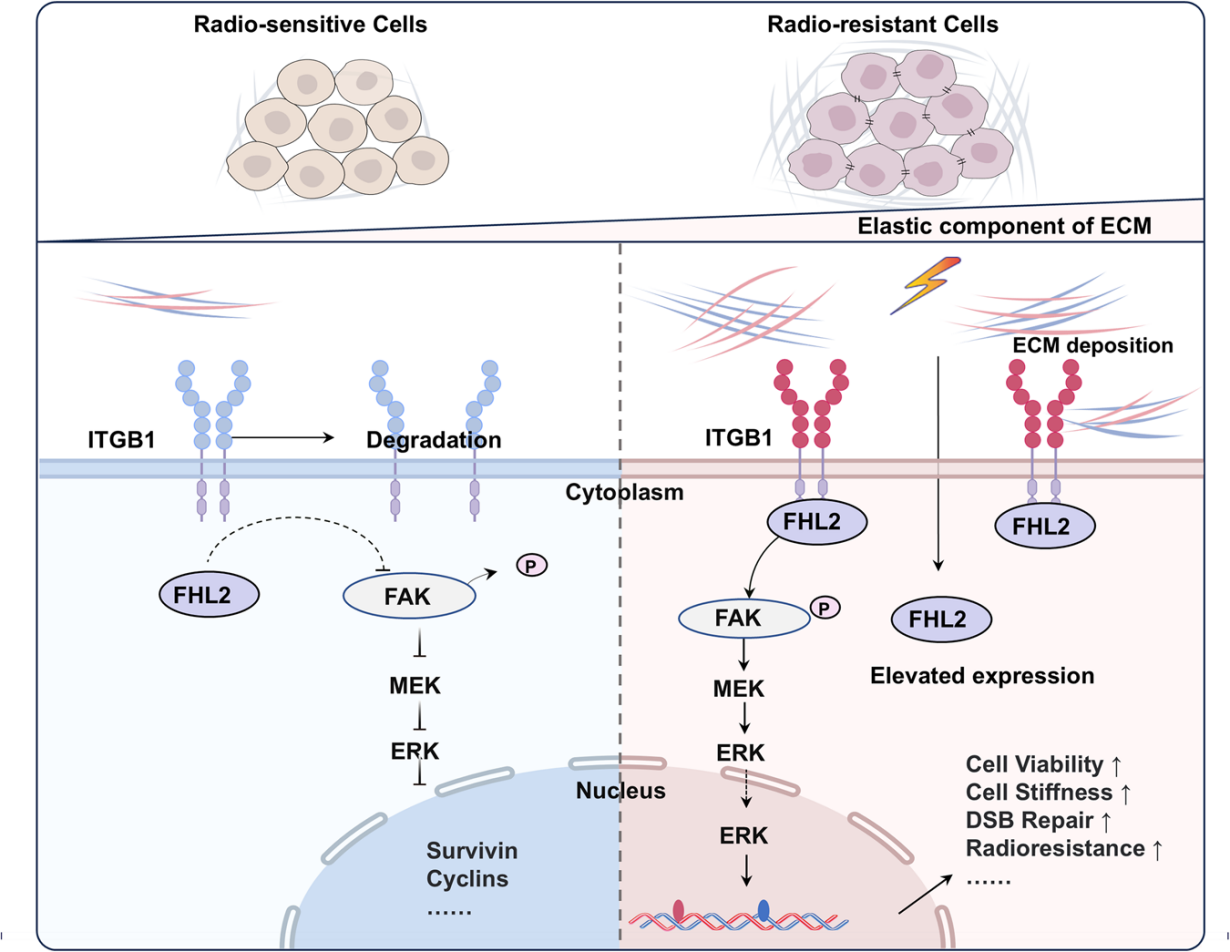
Schematic diagram of FHL2-mediated modulation of FAK/MAPK signaling in radioresistance of NSCLC.

This study investigates the mechanisms by which FHL2 mediates radioresistance in NSCLC, focusing on its role in ECM remodeling and activation of the FAK/MAPK signaling pathway. Supported by bioinformatics analysis and in vitro and in vivo experiments, the study results revealed that elevated FHL2 expression strongly correlated with increased radioresistance in NSCLC cells. Specifically, FHL2 interacted with ITGB1 to stabilize the ITGB1 protein, subsequently activating the FAK/MAPK signaling pathways, and significantly influencing cellular stiffness and ECM remodeling (Fig. 8). This interaction enhanced cellular stiffness and adapted the tumor microenvironment, intensifying the NSCLC cell resistance to radiotherapy.

FHL2 upregulation critically drives dedifferentiation and malignant progression across diverse cancer types. Previous studies demonstrate that in esophageal squamous cell carcinoma, FHL2 interacts with TAB182 to modulate the G2-M checkpoint, thereby conferring radioresistance [24]. Additionally, FHL2 enhances the MDM2-mediated degradation of IER3, which regulates the proliferation of cervical cancer cells [25]. The FHL2-GLI2 fusion is hypothesized to function as the primary oncogenic driver in ovarian tumors, defining the genotypic-phenotypic association of these neoplasms [26]. Notably, stiffness-mediated FHL2 signaling induces chemoresistant dormancy in basal cell carcinoma, mirroring its mechanosensitive role in therapy evasion [27]. However, FHL2 exhibits tumor-suppressive phenotypes in specific contexts. In hepatocellular carcinoma, FHL2 functions independently of tumor growth inhibition, exerting anti-apoptotic effects [28]. In addition, studies have shown that FHL2 mitigates the tumorigenesis of gastrointestinal stromal tumors by negatively regulating KIT signal transduction [19]. Therefore, these findings support the potential of FHL2 as a multifaceted therapeutic target, providing critical mechanistic context for its role in NSCLC.

In line with previous evidence that FHL2 was identified as an oncogene in multiple cancers, our study revealed that this expression was not only increased in NSCLC compared to normal lung tissue, but also more pronounced in the radioresistant cell model. Notably, by constructing stable NSCLC transfectants overexpressing FHL2, it was observed that the upregulated FHL2 expression amplified the production of key ECM components, thereby enhancing the structural integrity and stiffness of tumor cells. Additionally, this upregulation featured the EMT phenotype characterized by an increase in mesenchymal markers, including N-cadherin, α-SMA, and vimentin, and a corresponding decrease in the epithelial marker E-cadherin. This ECM deposition and EMT phenotype transition is not only confirmed to be associated with radiation-induced fibrosis [29], but also closely connected to subsequent alterations in the tumor microenvironment and enhanced tumor invasiveness [4]. Morphologically, these alterations were characterized by an enlarged cytoskeleton and significant aggregation of stress fibers. These modifications increased the structural complexity and functional dynamics of the cells, leading to heightened migration and enhanced resistance to radiotherapy in NSCLC cells. These results suggest that FHL2 may influence cell polarity, differentiation, proliferation, migration, and invasion by regulating cell adhesion molecules and integrin interactions. Conversely, the reversal of the above phenotypic alterations was observed in radioresistant NSCLC cell models following FHL2 knockdown.

Focal adhesion kinase (FAK) is a non-receptor tyrosine kinase associated with cytoplasmic adhesion complexes, and it is widely overexpressed in multiple cancers, including ovarian, lung, and colorectal cancers [30]. Serving dual roles as a kinase and a scaffolding protein, FAK regulates crucial biological processes such as cell proliferation and migration [31]. Increasing evidence suggests that FAK exerts its kinase activity by directly—or in cooperation with proteins such as EGFR—indirectly activating ERK, thereby promoting angiogenesis or tumor progression in lung and colorectal cancers [32, 33]. In addition, the negative regulatory role of FAK in tumor radiosensitization has been increasingly substantiated in recent years [34, 35]. FAK inhibition could remodel the extracellular matrix and activate interferon signaling to restore tumor immunity, thereby enhancing the radiotherapy sensitivity of pancreatic ductal adenocarcinoma [36]. In nasopharyngeal carcinoma research, FAK enhances tumor cell resistance to radiotherapy by activating the AKT/JNK pathway through its phosphatase activity [37]. MAPK, as a key signaling molecule for resisting DNA damage and mediating EMT under ionizing radiation, can be activated by FAK in NSCLC to promote HIF1-α accumulation, thereby significantly enhancing tumor growth and migration [32]. Therefore, we hypothesize that the FAK/MAPK pathway is likely a crucial target for investigating the mechanisms of NSCLC radioresistance. In the signaling network of interactions between cells and the extracellular matrix, ITGB1 plays a crucial role. ITGB1 is a key integrin β subunit that interacts with various α subunits to form receptors capable of recognizing and binding ECM proteins like fibronectin and collagen [38]. This interaction facilitates the aggregation of structural and signaling proteins, promoting the interaction of FAK with the cytoplasmic tail of ITGB1, leading to the phosphorylation of tyrosine at position 397 (pFAK Tyr397) [39, 40]. This autophosphorylation event triggers an amplification of cellular signaling cascades, ultimately activating the MAPK/ERK pathway, thereby effectively bridging extracellular signals to intracellular processes, while also regulating essential cellular functions such as proliferation and migration.

Our study found that FHL2 upregulation enhances the expression and stabilization of ITGB1 protein, which is pivotal in activating the FAK/MAPK signaling pathway and promoting ECM remodeling. The phosphorylation-triggered activation of FAK, driven by the clustering of ITGB1-containing integrin complexes, not only enhances signal transduction but also critically regulates cellular functions like survival and therapeutic resistance [41–43]. However, the knockdown of ITGB1 effectively reversed the FHL2-driven radioresistance. This observation aligns with previous research demonstrating that ITGB1 may enhance tumor radioresistance by regulating the DNA damage response and facilitating EMT [44]. These mechanistic insights were further corroborated in xenograft mouse models, where disruption of the FHL2/ITGB1 axis significantly attenuated tumor radioresistance and suppressed EMT progression. Clinically, elevated FHL2 expression correlated with poorer prognosis. Prognostic stratification revealed that NSCLC patients exhibiting elevated FHL2/ITGB1 co-expression demonstrated accelerated disease progression, underscoring their clinical utility. Mechanistically, targeting this axis resensitized tumors to radiotherapy through coordinated EMT reversal and suppression of cytoskeletal hyperactivation. These findings advocate for combinatorial therapeutic strategies integrating FHL2 inhibition with radiation to overcome treatment resistance, with translational potential extending beyond NSCLC to malignancies characterized by adaptive stress survival mechanisms.

## Conclusion

In conclusion, this study underscores the critical role of FHL2 in promoting radioresistance in NSCLC. Mechanistically, FHL2 stabilizes ITGB1 by inhibiting ubiquitin-proteasomal degradation, thereby reinforcing the cytoskeletal structure and cellular stiffness, and driving ECM remodeling and EMT progression via the FAK/MAPK signaling pathway. Targeting the FHL2/ITGB1 axis notably improved the efficacy of radiotherapy in vivo. Overall, these findings position FHL2 as a promising biomarker for radioresistance and a valuable therapeutic target to combat this resistance in NSCLC.

## Materials and methods

### Cell lines

Human NSCLC lines A549 and H1299 were sourced from the Cell Bank Type Culture Collection at the Chinese Academy of Sciences (Shanghai, China). A549 were grown in DMEM and H1299 were in RPMI-1640 (Gibco, USA) in a 37 °C humidified incubator with 5% CO2. Radioresistant models were established by irradiating cells with 6 Gy per exposure, administered at rates of 1.02 Gy/min for A549 and 0.75 Gy/min for H1299, for a total of five exposures (30 Gy) using an X-ray device (X-RAD320iX, USA). The selective FAK inhibitor PF-562271 was applied at 5 µM for 24 h, with an equivalent dose of DMSO added as control.

### Animal study

Female BALB/c-nu nude mice (4–5 weeks old, 18–20 g) were purchased from Sibeifu Biotechnology Co., Ltd. (Beijing, China). All protocols were approved by the Institutional Animal Care and Use Committee of Jilin University, and all animals were managed at the laboratory animal centers in accordance with its guidelines (No. SY202401016), and all animals were housed under specific pathogen-free (SPF) conditions at the institutional laboratory animal center with controlled temperature (22 ± 2 °C), humidity (55 ± 10%), and 12 h light/dark cycles. After one week of acclimatization, mice were randomly divided into 8 groups (*n* = 7 per group). Mice were inoculated with A549 (CON/FHL2^OE^/ITGB1^KD^/FHL2^OE^-ITGB1^KD^) cells to establish a cell-derived xenograft model for irradiation and non-irradiation treatments. 1 × 10^6^ cells was injected subcutaneously into the right limb of each mouse. Health status and tumor volumes were regularly monitored, using the formula: Tumor volume = *a* × *b*² / 2, where *a* is tumor length (mm) and *b* is tumor width (mm). A 20 Gy radiation dose was applied when tumors achieved an average size of 200 mm³. All animals were humanely euthanized 14 days post-radiotherapy to minimize suffering and reach the study endpoint.

### Lentiviral vector synthesis and transfection

Cells were transduced with lentiviruses encoding shFHL2 or shITGB1 to achieve knockdown of the target genes, while the control group was transduced with lentiviruses containing negative control sequences. For target gene overexpression, cells were transfected with plasmids encoding FHL2, whereas the control group was transfected with empty vector controls. Transfections were performed following the guidelines with Lipofectamine 2000 (Thermo Fisher, USA). Cells were cultured in a solution of 2 μg/mL puromycin (Solarbio, China) for a minimum of two weeks to select for stably transfected cells through resistance. The transfection efficiency was assessed using the western blotting method. The sequences of related plasmids and shRNA oligonucleotides are shown in Supplementary Table 1.

### Western blotting

Total protein extracts were obtained using RIPA (Beyotime, China). Equal quantities of protein for each sample were separated by SDS-PAGE, and subsequently transferred onto PVDF membranes, and probed with primary antibodies for immunodetection, followed by secondary antibodies for chemiluminescent detection using Pierce ECL kit (Thermo Fisher, USA). Details of the primary and secondary antibodies employed in this study are listed in Supplementary Table 2.

### Comet assay

The neutral comet assay was conducted with the comet assay kit (Beyotime, China). Cells were embedded in low-melting agarose on comet slides, followed by electrophoresis and propidium iodide staining as the manufacturer’s instructions. Fluorescence microscopy captured cell images, and CometScore software assessed the olive tail moment. Additional methods are detailed in the supplementary materials.

## Supplementary information

Supplementary Figures and Tables

Supplementary Methods

Original Western Blots data

## Data Availability

The data and materials supporting the findings of this study are included in the article or supplementary information files, and further inquiries can be directed to the corresponding author. The RNA-seq data from this study are available in the NCBI BioProject database under accession ID PRJNA1297386.
