# Supplementary material for: FHL2 enhances ITGB1-mediated ECM remodeling and cellular stiffness to promote radioresistance in non-small cell lung cancer
Source: Cell Death Discov. 2025 Oct 24;11:480. doi: 10.1038/s41420-025-02757-6 (PMC12552696; doi:10.1038/s41420-025-02757-6)
Supplement: Supplementary file 1 — Supplementary Figures and Tables [file 41420_2025_2757_MOESM1_ESM.docx]

**Supporting Information**

**FHL2 Enhances** **ITGB1-Mediated ECM Remodeling and Cellular Stiffness to Promote Radioresistance in Non-Small Cell Lung Cancer**

Xiaoyu Pu ^1^, Kexin Chen ^2^, Lihua Dong ^1^, Junxuan Yi ^3^, Mingwei Wang ^3^, Xinfeng Wei ^3^, Mingqi Zhao ^3^, Mengdie Zhao ^3^, Xinyan Wang ^3^, Lijuan Ding ^1*^, Shunzi Jin ^3*^

**Affiliation**

^1^ Jilin Provincial Key Laboratory of Radiation Oncology & Therapy, Department of Radiation Oncology & Therapy, The First Hospital of Jilin University, Changchun, China;

^2^ Institute of Translational Medicine, The First Hospital of Jilin University, Changchun, China.

^3^ National Health Commission Key Laboratory of Radiobiology, School of Public Health, Jilin University, Changchun, China

*** Correspondence:**

Shunzi Jin, E-mail: [jinsz@jlu.edu.cn](mailto:jinsz@jlu.edu.cn); National Health Commission Key Laboratory of Radiobiology, School of Public Health, Jilin University, Changchun 130021, China.

Lijuan Ding, E-mail: dinglijuan@jlu.edu.cn; Department of Radiation Oncology & Therapy, The First Hospital of Jilin University, Changchun 130021, China.

**Supplementary Figures and Tables:**

**
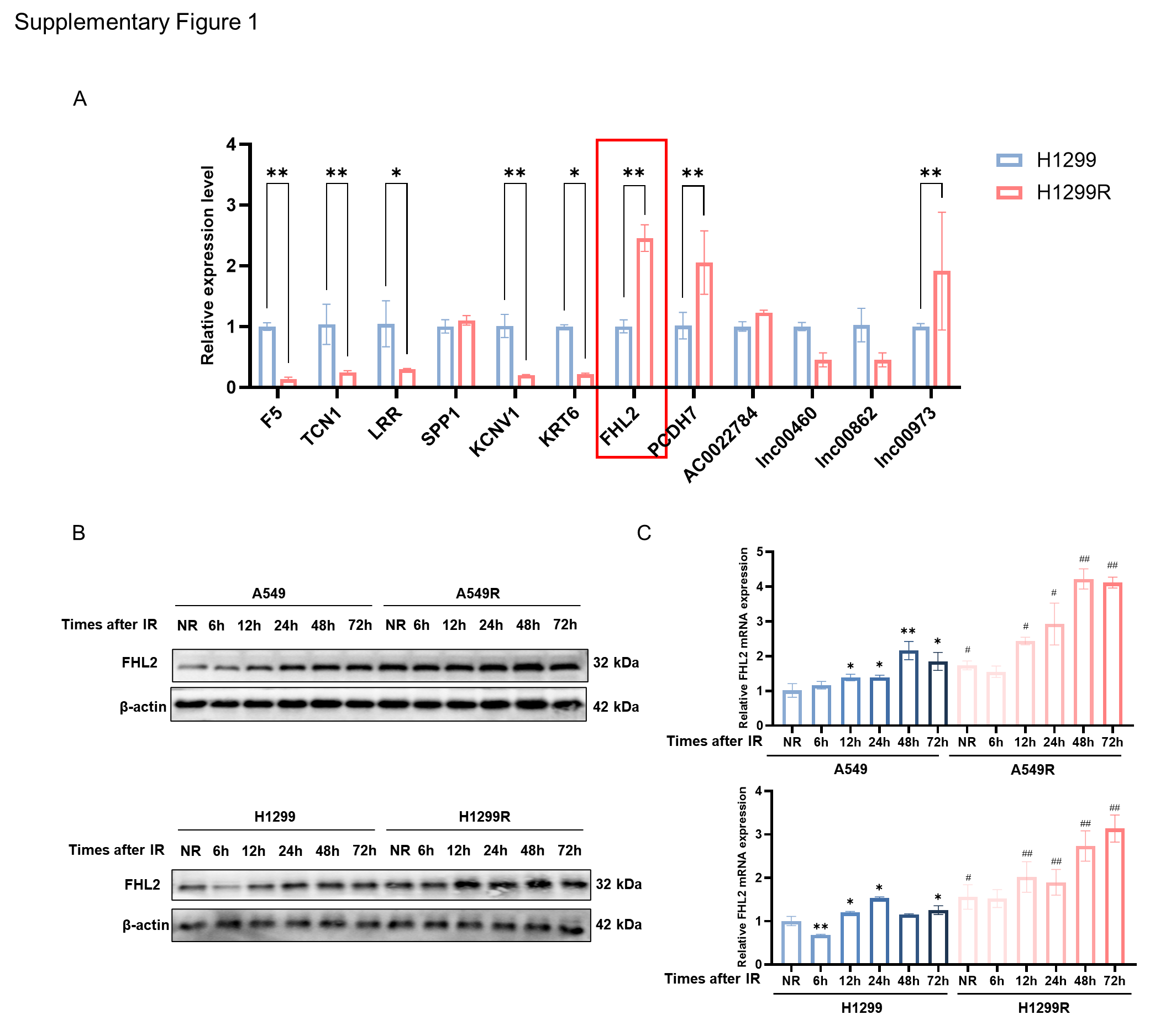
Supplymentary Fig. 1****: Identification and Radiation-Responsive Expression Dynamics of FHL2 in Radioresistant NSCLC.** (A) qPCR analysis of the relative expression levels of the 12 candidates in radioresistant H1299R cells compared to H1299 cells (n = 3). (B) Western blotting and (C) qPCR analysis showing the expression levels of FHL2 protein and mRNA before irradiation and at 6, 12, 24, 48, and 72 hours post-irradiation. ^*^ *p* < 0.05, ^**^ *p* < 0.01, ^#^ *p* < 0.05, ^##^ *p* < 0.01.


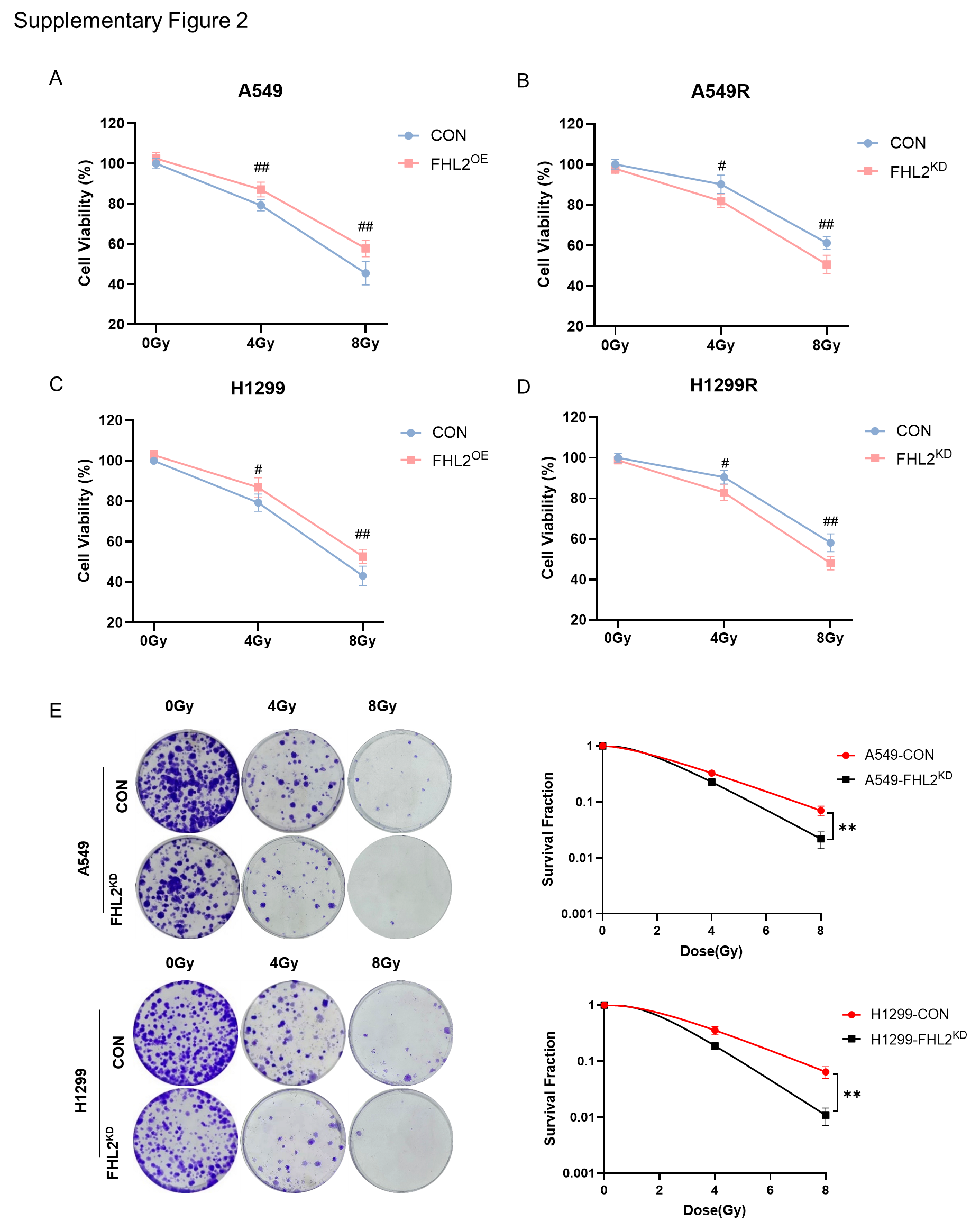


**Supplementary Fig. 2: Impact of FHL2 on Cell Viability and Survival Following Irradiation.** (A-D) The CCK8 test assessed cell viability and proliferation in A549 and H1299 cells with FHL2 overexpression, and A549R and H1299R cells with FHL2 knockdown, under irradiation doses of 0, 4 and 8 Gy (n = 3). (E) Clonogenic assays indicate a reduced survival rate of A549 and H1299 cells following FHL2 knockdown post-irradiation (n = 3). ^*^ *p* < 0.05, ^**^ *p* < 0.01, ^#^ *p* < 0.05, ^##^ *p* < 0.01.


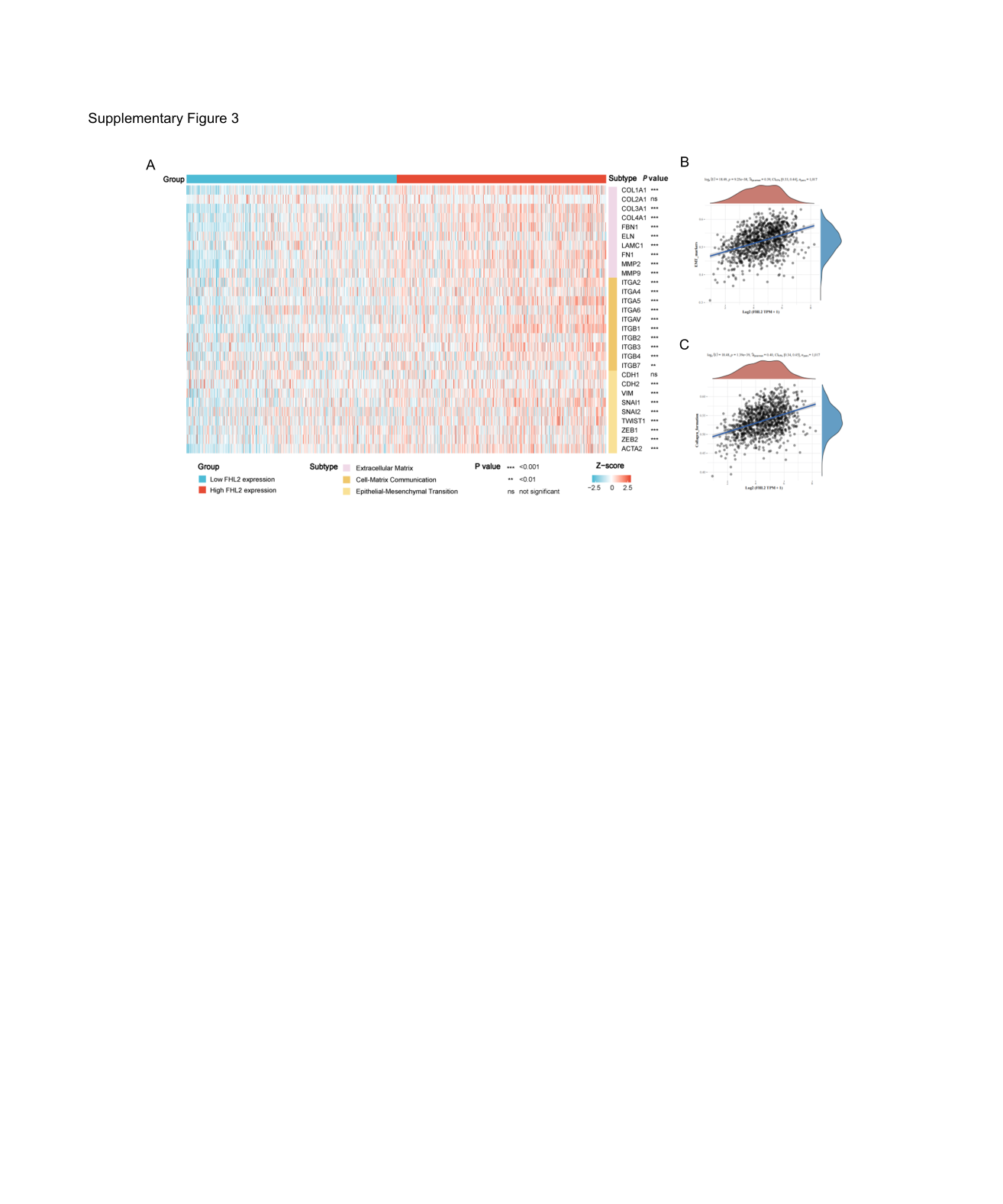


**Supplymentary Fig. 3: Differential Expression of ECM, EMT, and Cell-matrix Interaction Genes in Parental and Radioresistant NSCLC Cells.** (A) Heatmap of correlations between FHL2 and genes involved in ECM, EMT, and cell-matrix interactions based on TCGA database analysis of NSCLC samples. (B-C) Correlation analysis between FHL2 and pathway scores reveals statistically significant correlations between FHL2 and EMT pathways as well as collagen formation pathways.


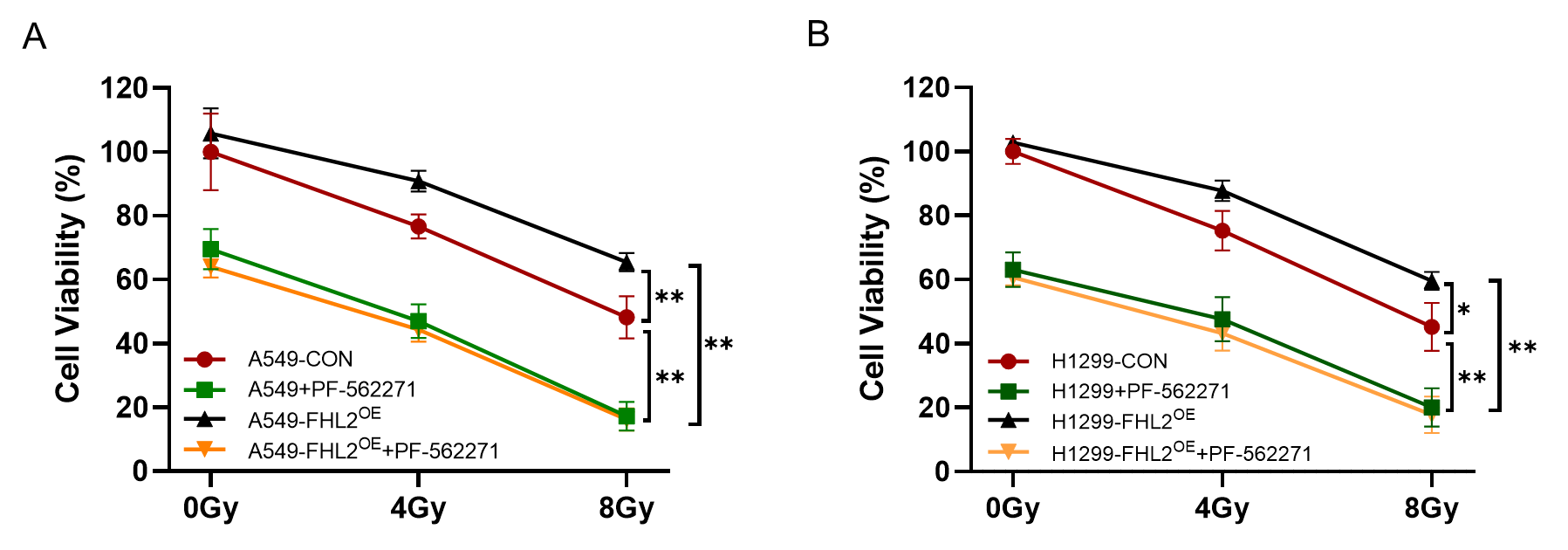


**Supplementary Fig. 4: CCK-8 viability assessment post-FAK inhibition in irradiated NSCLC cells.** The CCK-8 assays demonstrating the FAK inhibitor PF-562271 decreased survival rates of irradiated A549 and H1299 cells including those overexpressing FHL2 (n=3). ^*^ *p* < 0.05, ^**^ *p* < 0.01.


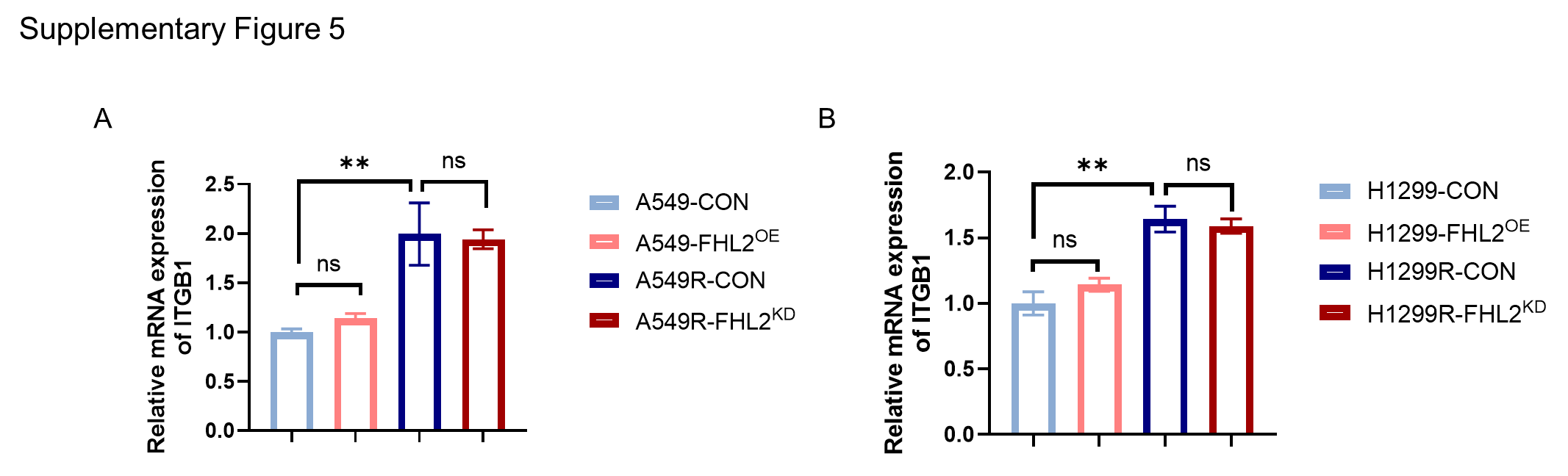


B

B

**Supplementary Fig. 5: ITGB1 mRNA Expression in Parental and Radioresistant NSCLC cells.** (A) ITGB1 mRNA levels after overexpression of FHL2 in A549 cells, and knockdown of FHL2 in A549R cells. (B) ITGB1 mRNA levels after overexpression of FHL2 in H1299 cells, and knockdown of FHL2 in H1299R cells. ^**^ *p* < 0.01, ns = not significant.

**
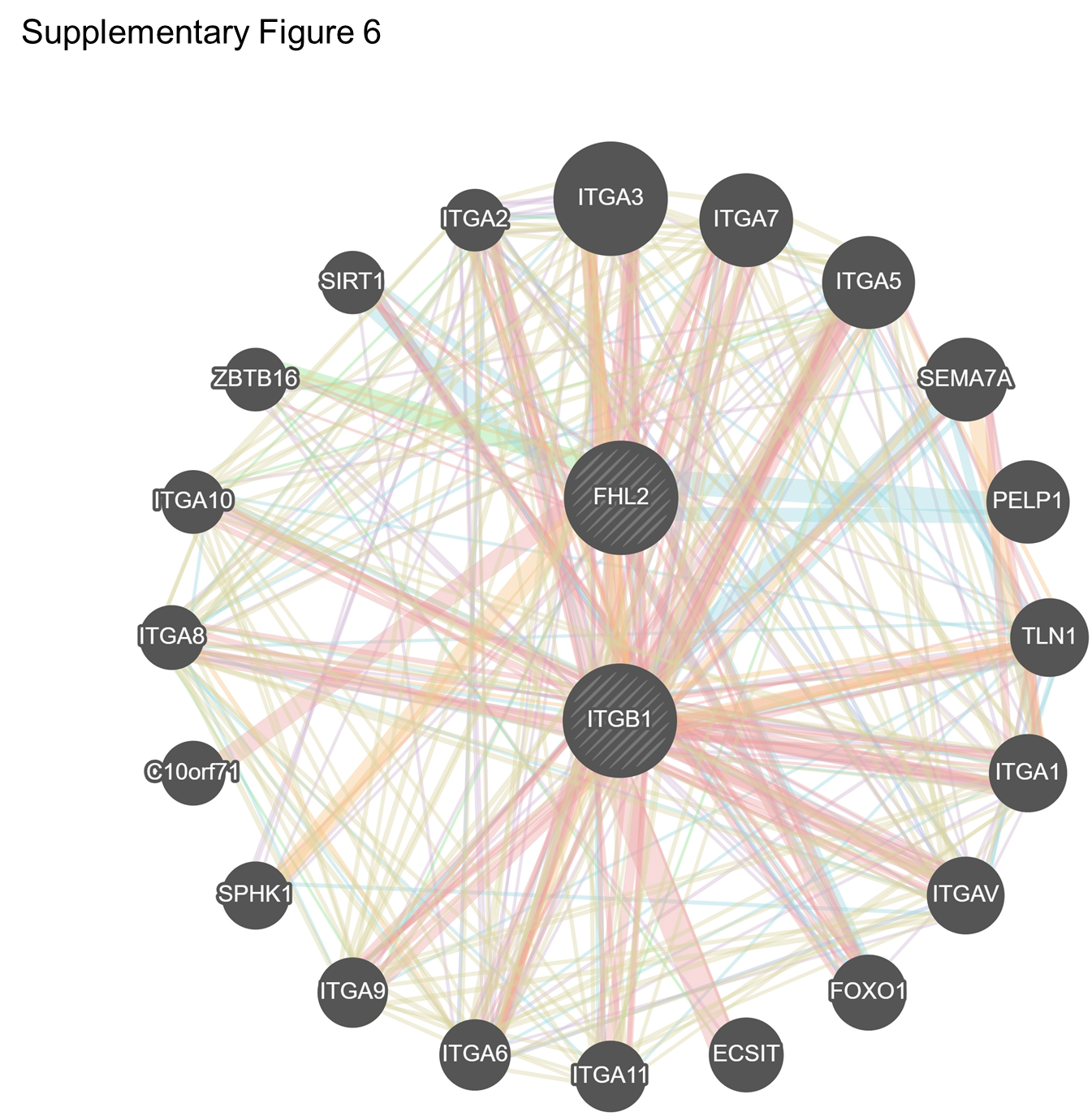
**

**Supplementary Fig. 6: Prediction of the FHL2-ITGB1 Interaction Based on the GeneMANIA Database.**

**
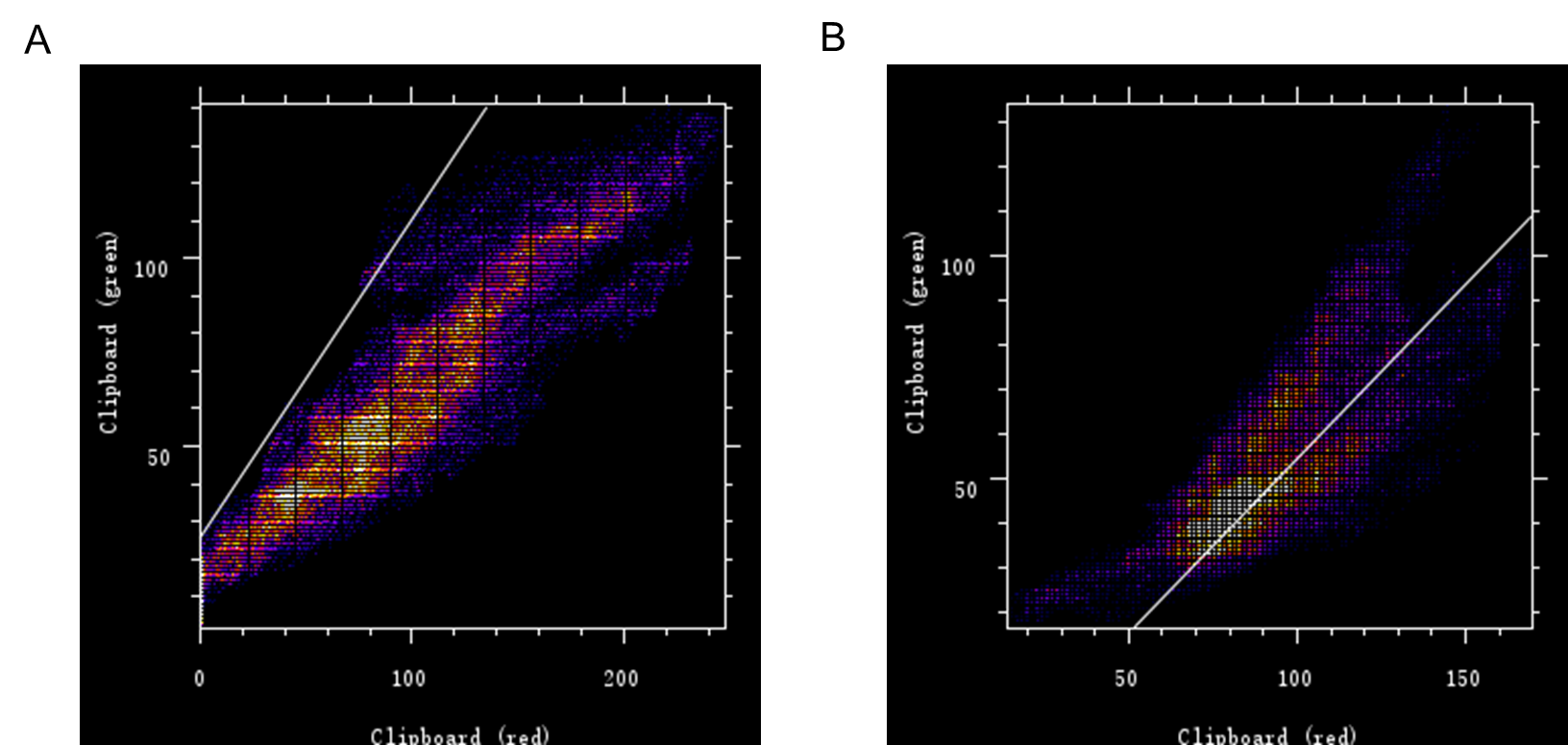
**

**Supplementary Fig. 7: FHL2-ITGB1 colocalization scatter plots in NSCLC.** Colocalization scatter plots of FHL2 and ITGB1 in (A) A549 and (B) H1299 with Pearson coefficients of (A) 0.91 and (B) 0.78.

**
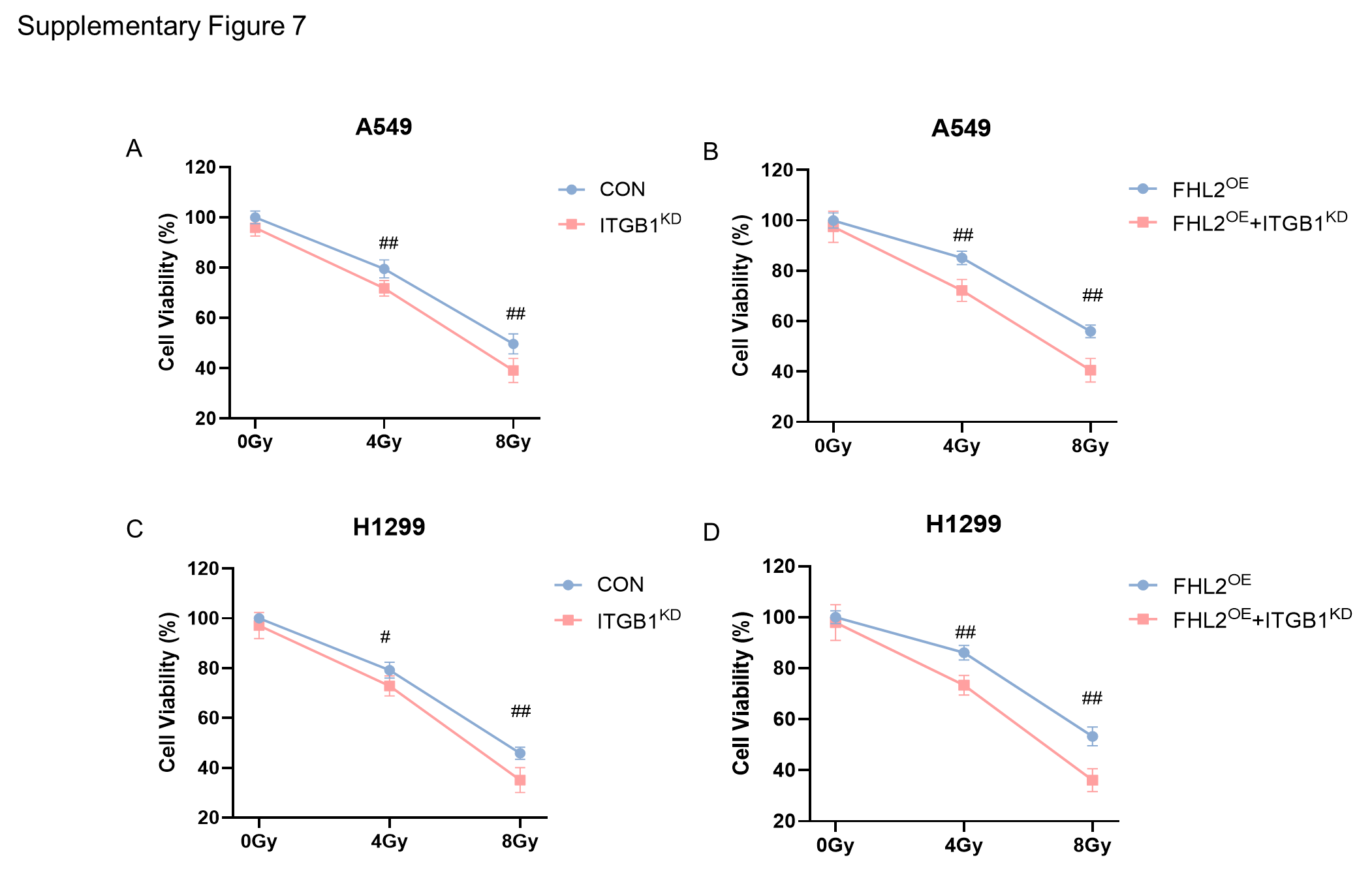
**

**Supplementary Fig. 8: Impact of ITGB1 on Cellular Proliferation Following Irradiation.** (A-D) The CCK8 test assessed cell viability and proliferation in A549, A549-FHL2^OE^, H1299, and H1299-FHL2^OE^ cells with ITGB1 knockdown, under irradiation doses of 0, 4 and 8 Gy (n = 3). ^#^ p < 0.05, ^##^ p < 0.01.


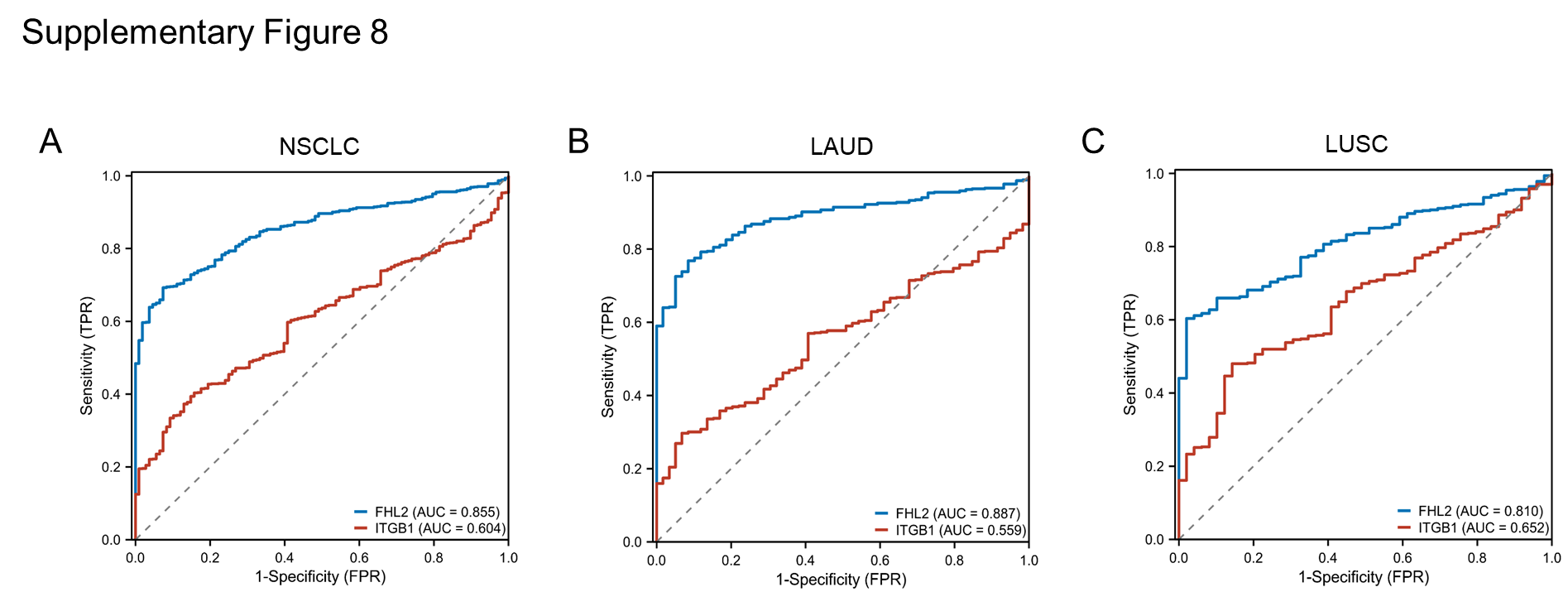


**Supplementary Fig. 9: Diagnostic ROC Curve of FHL2 and ITGB1 for NSCLC.**

The diagnostic ROC curves of FHL2 and ITGB1 for (A) NSCLC, including (B) LUAD and (C) LUSC.

**Supplementary Table 1: The sequences of** **plasmids and shRNAs**

| plasmids | Sequence |
| --- | --- |
| FHL2 | NM_001450.4 |
| shFHL2 #1 | CGACTGCTTTAACTGTAAGAA |
| shFHL2 #2 | CGAATCTCTCTTTGGCAAGAA |
| shFHL2 #3 | CCAATTGGAACCAAGAGTTTC |
| shITGB1 #1 | GCCTTGCATTACTGCTGATAT |
| shITGB1 #2 | GCCCTCCAGATGACATAGAAA |
| shITGB1 #3 | CCAAATCATGTGGAGAATGTA |

**Supplementary Table 2: A list of all antibodies used in this work and dilutions.**

| Antibodies | Catalog number | Company | Dilutions |
| --- | --- | --- | --- |
| Anti-FHL2 | AB202584 | Abcam | 1:1000 |
| Anti-ITGB1 | PA5-78028 | ThermoFisher | 1:5000 |
| Anti-Collagen-1 | BS70155 | Bioworld | 1:1000 |
| Anti-Fibronectin-1 | MB63314 | Bioworld | 1:1000 |
| Anti-E-cadherin | BS1098 | Bioworld | 1:1000 |
| Anti-N-cadherin | BS72312 | Bioworld | 1:1000 |
| Anti-Vimentin | YM3158 | Immunoway | 1:1000 |
| Anti-α-SMA | YM3364 | Immunoway | 1:1000 |
| Anti-FAK | bs-1340R | Bioss | 1:1000 |
| Anti-phosphorylated FAK | bs-3159R | Bioss | 1:1000 |
| Anti-ERK | bs-2637R | Bioss | 1:1000 |
| Anti-phosphorylated ERK | bs-3016R | Bioss | 1:1000 |
| Anti-β-actin | AP0060 | Bioworld | 1:1000 |
| Goat anti-Mouse IgG (H+L) HRP | BS12478 | Bioworld | 1:50000 |
| Goat anti-Rabbit IgG (H+L) HRP | BS13278 | Bioworld | 1:10000 |
| Cy3 goat anti-Mouse antibody | GB21303 | Servicebio | 1 : 200 |
| Cy3 goat anti-Rabbit antibody | GB21301 | Servicebio | 1 : 200 |
| Normal Rabbit IgG | 2729S | CST | 1:100 |
