# Supplementary material for: FHL2 enhances ITGB1-mediated ECM remodeling and cellular stiffness to promote radioresistance in non-small cell lung cancer
Source: Cell Death Discov. 2025 Oct 24;11:480. doi: 10.1038/s41420-025-02757-6 (PMC12552696; doi:10.1038/s41420-025-02757-6)
Supplement: Supplementary file 2 — Supplementary Methods [file 41420_2025_2757_MOESM2_ESM.docx]

**Supporting Information**

**FHL2 Enhances** **ITGB1-Mediated ECM Remodeling and Cellular Stiffness to Promote Radioresistance in Non-Small Cell Lung Cancer**

Xiaoyu Pu ^1^, Kexin Chen ^2^, Lihua Dong ^1^, Junxuan Yi ^3^, Mingwei Wang ^3^, Xinfeng Wei ^3^, Mingqi Zhao ^3^, Mengdie Zhao ^3^, Xinyan Wang ^3^, Lijuan Ding ^1*^, Shunzi Jin ^3*^

**Affiliation**

^1^ Jilin Provincial Key Laboratory of Radiation Oncology & Therapy, Department of Radiation Oncology & Therapy, The First Hospital of Jilin University, Changchun, China;

^2^ Institute of Translational Medicine, The First Hospital of Jilin University, Changchun, China.

^3^ National Health Commission Key Laboratory of Radiobiology, School of Public Health, Jilin University, Changchun, China

*** Correspondence:**

Shunzi Jin, E-mail: [jinsz@jlu.edu.cn](mailto:jinsz@jlu.edu.cn); National Health Commission Key Laboratory of Radiobiology, School of Public Health, Jilin University, Changchun 130021, China.

Lijuan Ding, E-mail: dinglijuan@jlu.edu.cn; Department of Radiation Oncology & Therapy, The First Hospital of Jilin University, Changchun 130021, China.

**Supplementary Methods**

- 1. **Colony Formation Assay**

1000 cells were plated in six-well plates and exposed to X-ray radiation (0, 4, and 8 Gy) the following day. After 2 weeks, the colonies were washed thrice with PBS, fixed in 4% paraformaldehyde (Solarbio, China) for 20 minutes, and stained with crystal violet (Solarbio, China) for 25 minutes. Finally, the number of colonies per plate was counted.

**1.2. Flow Cytometry Analysis**

Cells were irradiated with either 0 or 8 Gy and harvested using trypsin 24 hours post-irradiation. Cell apoptosis was assessed by staining with Annexin V-FITC/PI (Meilunbio, China) according to the instructions. To analyze the cell cycle, ethanol-fixed cells were stained with PI (Biotopped, China) according to the instructions.

- 1. **Cell Migration Assay**

1 × 10^5^ cells in serum-free medium were seeded in the upper cavity of the insert (Millipore, USA). A medium containing 10% fetal bovine serum was supplemented to the lower cavity. After a 24-h incubation period, the cells in the upper cavity were removed, and the cells on the surface of the lower membrane were fixed, dyed with 0.5% crystal violet and then counted.

- 1. **Immunofluorescence**

For intracellular staining, the cells were washed with PBS, fixed using 4% paraformaldehyde (Solarbio, China), and then permeabilized with 0.5% Triton X-100 (Sigma, USA). They were then washed with PBS, sealed with 10% bovine serum albumin for 1 h, and incubated overnight at 4°C with specific primary antibodies at a dilution of 1:200. And then, the samples were washed with PBS and incubated with a 1:200 dilution of fluorescently labeled secondary antibody (Servicebio, China) for 1 h. A sealing agent concluding DAPI was then applied, and the samples were kept at 4°C in a dry container. A fluorescence microscope (Olympus, Japan) was utilized for imaging.

- 1. **Immunohistochemistry**

Tumor sections (5 µm) were subjected to a process of deparaffinisation and rehydration. The endogenous peroxidase was inactivated by the addition of 3% hydrogen peroxide for a period of 10 minutes. Antigen retrieval was conducted using a citrate buffer at 95°C for 20 min. The sections were treated with 5% goat serum for 1 h and incubated approximately 12h at 4°C with primary antibody. After rinsing, sections were treated with biotinylated secondary antibody for 1 h and streptavidin-biotin complex with horseradish peroxidase for 30 min. Staining was developed using diaminobenzidine (DAB). The samples were then counterstained with hematoxylin, followed by dehydration and clearing.

- 1. **Immunoprecipitation**

Cell lysis buffer for Western and IP (Beyotime, China) with 1mM PMSF were used to perform immunoprecipitation. Protein lysates (400 μg) were incubated with 5 μg/ml of anti-FHL2 antibody (Abcam, UK) or anti-ITGB1 antibody (Thermo Fisher, USA) and incubated with gentle rotation overnight at 4°C. The immune complexes were precipitated by the addition of 25 μL of protein A/G agarose beads (Beyotime, China), which were then gently rotated for 6 h at 4°C. The cell lysates were then centrifuged at 1,200 *g* for 3 min at 4°C and the supernatant was removed. The beads were subjected to three washes with cold immunoprecipitation buffer, after which they were resuspended in sample buffer and heated for a period of 5 min to separate the immune complexes from the beads. The supernatant was obtained by centrifugation and analyzed by western blotting assay.

- 1. **Atomic Force Microscopy (AFM) Measurements**

AFM measurements were carried out with an AFM setup (HORIBA, Japan) in force mapping mode, employing a high-quality tip (MikroMasch, USA). The cantilever spring constant was 0.5 N. The tips were cleaned with ethanol and ultraviolet light after each force mapping to prevent contamination. The topography and Young’s modulus images were obtained to identify the bio-sample structures. The Young’s modulus maps on a scan area of 30 µm* 30 µm with 20 µm*20 µm force-indentation curves were tested to access the Young’s modulus for each cell sample.

- 1. **Structural Analysis and Molecular Docking**

We utilized the crystal structure of ITGB1_HUMAN (Uniprot ID: P05556) and the predicted full-length structure of FHL2_HUMAN (Uniprot ID: Q14192) from the Uniprot database (https://www.uniprot.org/) as the receptor and ligand proteins, respectively. Protein-protein docking was performed using the ZDOCK server, which integrates physical and bioinformatics methodologies to simulate the structure of the biomolecular complexes. The binding free energy was computed using the MM/GBSA method on the HawkDock server. Analysis of non-covalent interactions was conducted using the PLIP platform, with detailed structural visualization provided through pyMOL.

- 1. **Data Collection and Pre-Processing**

The RNAseq data were downloaded from the TCGA-LUAD and TCGA-LUSC projects via the TCGA database (https://portal.gdc.cancer.gov/). The data were processed using the STAR pipeline and represented in TPM format, alongside the corresponding clinical data. TPM data were log2-transformed (log2(value + 1)) for standardization. The radioresistant A549 cell dataset (GSE197236) from the GEO database was used to select differentially expressed genes.

**1.10. Prognostic Analysis**

Survival analysis was conducted using the survival (version 3.3.1), survminer (version 0.4.9), and ggplot2 (version 3.3.6) packages[1, 2]. Initially, the proportional hazards assumption was tested using the survival package, and a Cox proportional hazards regression model was fitted to assess the impact of variables on survival time. The surv_cutpoint function in the survminer package was then used for optimal cutoff point selection. Survival and cumulative risk curves were visualized by the survminer and ggplot2 packages, ensuring clear presentation of the results. The log rank test was employed for statistical comparisons.

**1.11. Diagnostic Analysis**

The data used for analysis comprised NSCLC tumor samples from TCGA and normal lung tissues from GTEx. Diagnostic analysis was conducted using the pROC (version 1.18.0) and ggplot2 (version 3.3.6) packages. The pROC package was employed for the purpose of conducting receiver operating characteristic (ROC) analysis, evaluating diagnostic test performance by plotting true positive rates against false positive rates. The generated ROC curves were then visualized using the ggplot2 package, ensuring clear and informative graphical representations of the diagnostic test performance.

**1.12. Gene Correlation Analysis**

The Spearman correlation analysis was used to calculate the associations between FHL2 and genes ralated to EMT and ECM, with visualization through a co-expression heatmap created using ggplot2 in R (version 4.0.3). Gene and pathway correlation analyses were performed with the GSVA package using the 'ssgsea' method, and Spearman correlation was applied to assess the association between genes and pathway scores [3]. A *P* value <0.05 was regarded as statisticaly significant.

**1.13. Statistical Analysis**

The results are the representative of experiments repeated at least three times and were
presented as mean ± SD. The experimental data were statistically analyzed using GraphPad Prism (version 8.0.2) and R software (version 4.0.3.). The statistical comparisons were conducted using the two-tailed Student's t-test and one-way ANOVA. The n reflects the number of biologically independent experiments in each group. A *P* value <0.05 was regarded as statistically significant.

**Reference:**

1. Zhang Z, Lin E, Zhuang H, et al. Construction of a novel gene-based model for prognosis prediction of clear cell renal cell carcinoma. Cancer Cell Int. 2020;20:27.

2. Lin W, Wu S, Chen X, et al. Characterization of Hypoxia Signature to Evaluate the Tumor Immune Microenvironment and Predict Prognosis in Glioma Groups. Front Oncol. 2020;10:796.

3. Wei J, Huang K, Chen Z, et al. Characterization of Glycolysis-Associated Molecules in the Tumor Microenvironment Revealed by Pan-Cancer Tissues and Lung Cancer Single Cell Data. Cancers (Basel). 2020;12(7).
