# Supplementary material for: FHL2 enhances ITGB1-mediated ECM remodeling and cellular stiffness to promote radioresistance in non-small cell lung cancer
Source: Cell Death Discov. 2025 Oct 24;11:480. doi: 10.1038/s41420-025-02757-6 (PMC12552696; doi:10.1038/s41420-025-02757-6)

Figure 1J

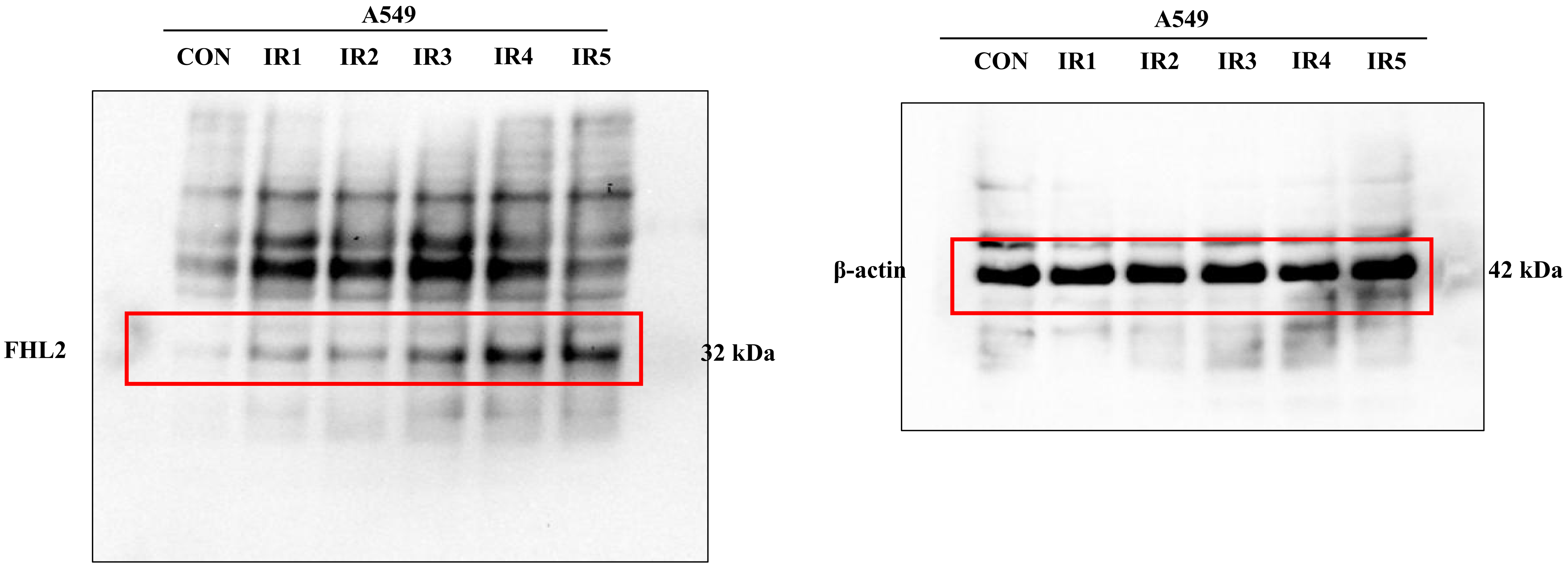
A549
CON
IR1
IR2
IR3
IR4
IR5
FHL2
32 kDa
A549
CON
IR1
IR2
IR3
IR4
IR5
β-actin
42 kDa

Figure 1J

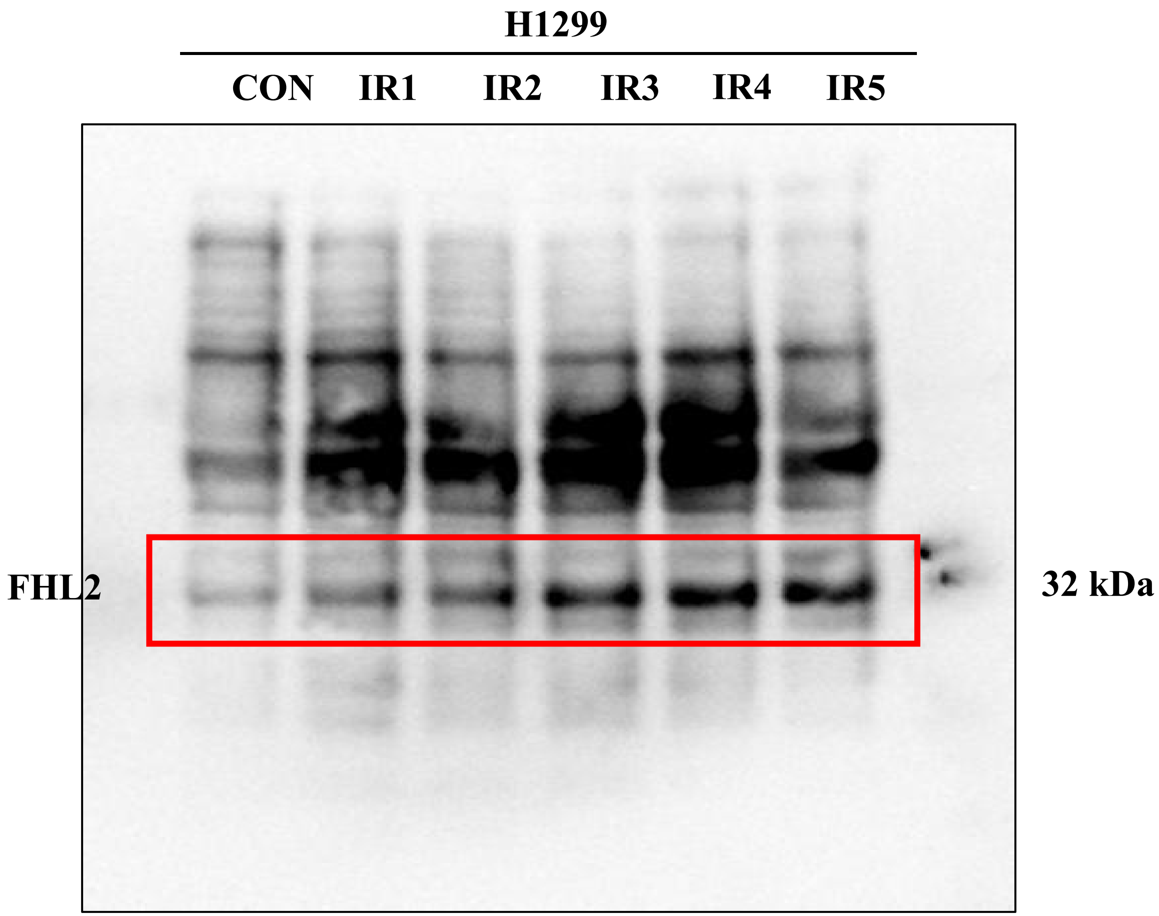
H1299
CON
IR1
IR2
IR3
IR4
IR5
FHL2
32 kDa

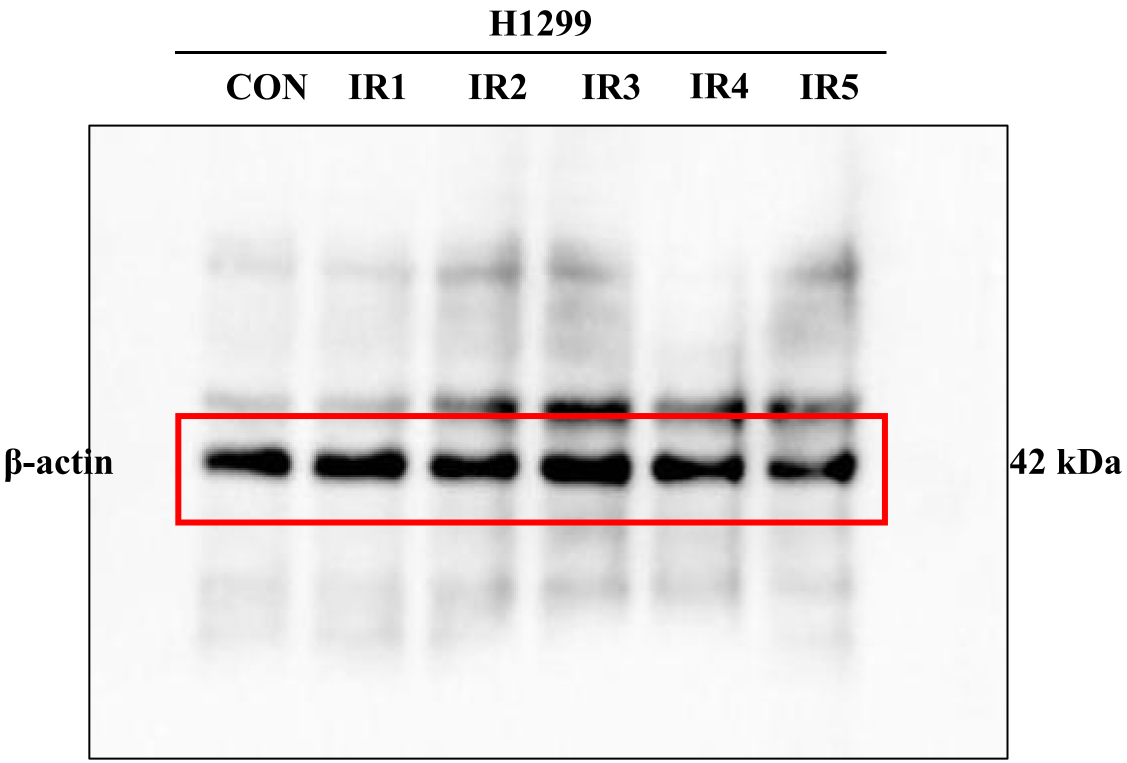
H1299
CON
IR1
IR2
IR3
IR4
IR5
β-actin
42 kDa

Figure 2A

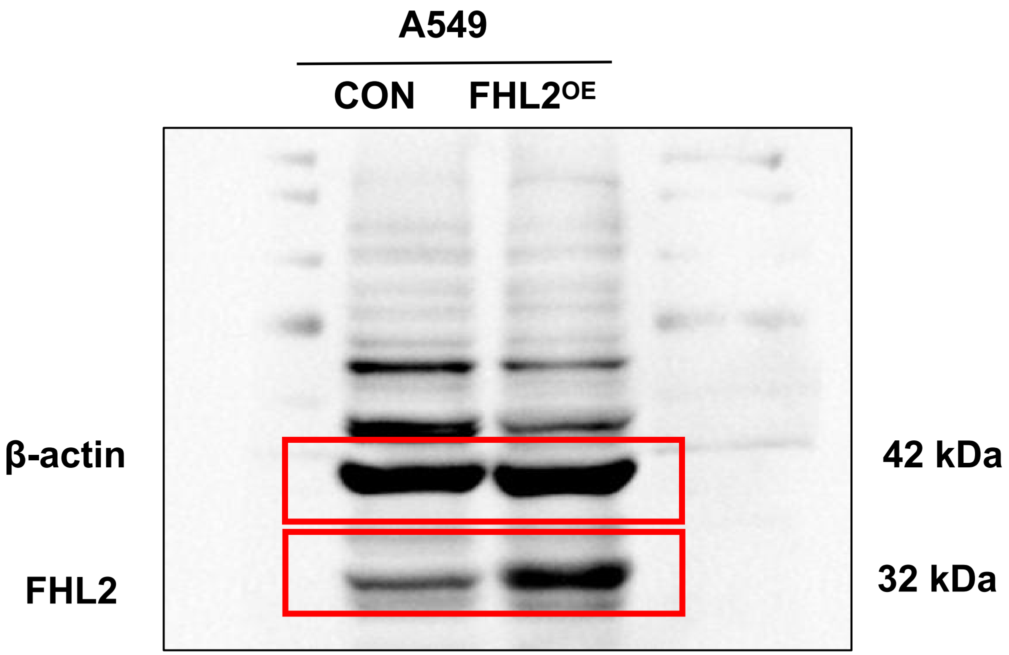

Figure 2A

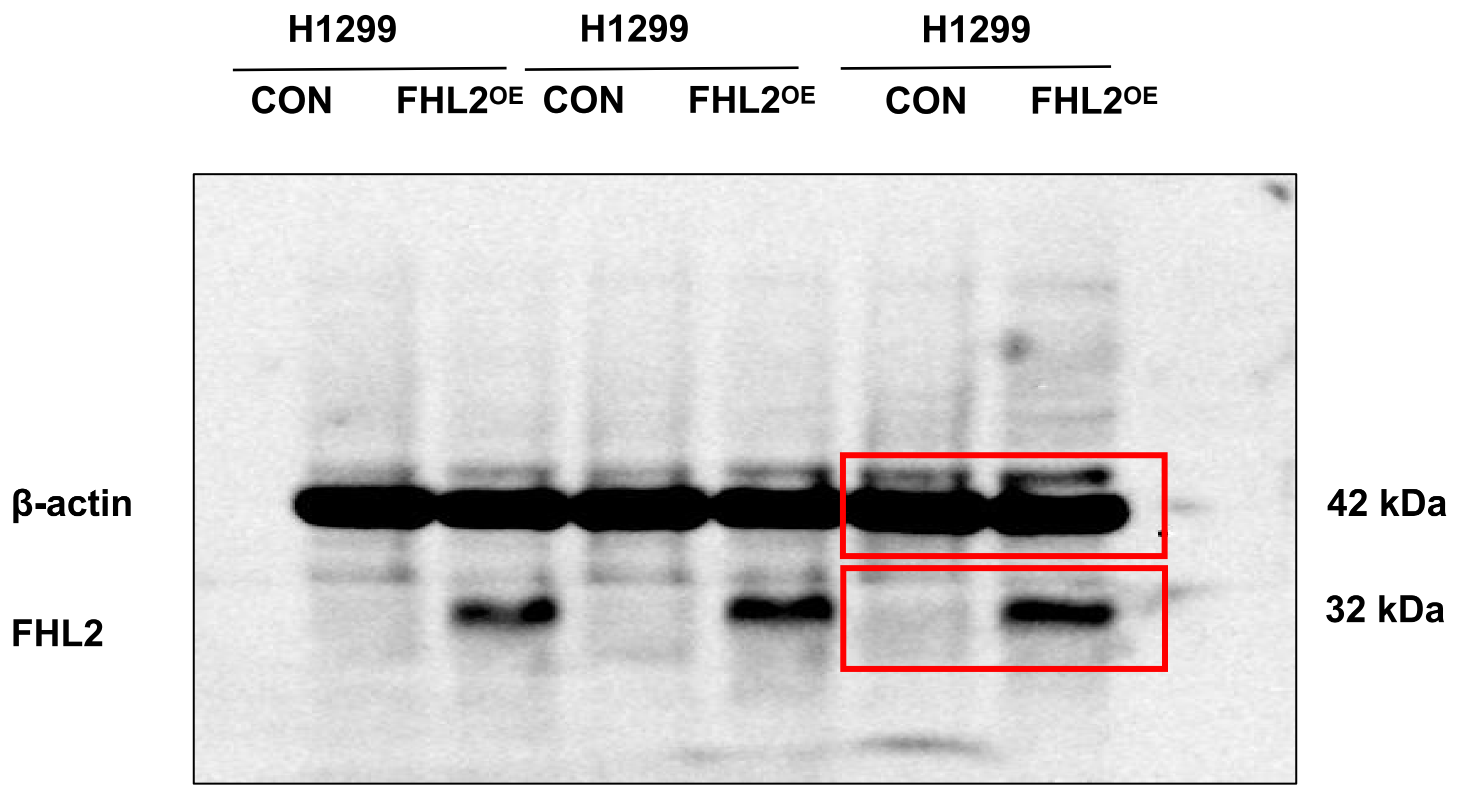
H1299
H1299
H1299
CON
FHL2OE
CON
FHL2OE
CON
FHL2OE
β-actin
FHL2
42 kDa
32 kDa

Figure 2B

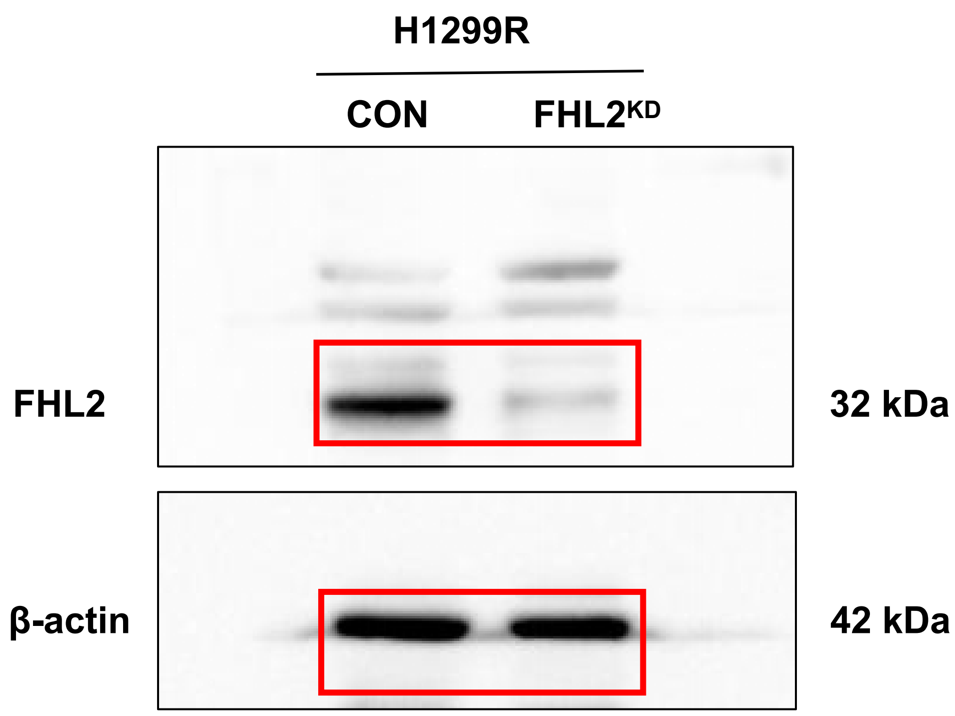

Figure 3D

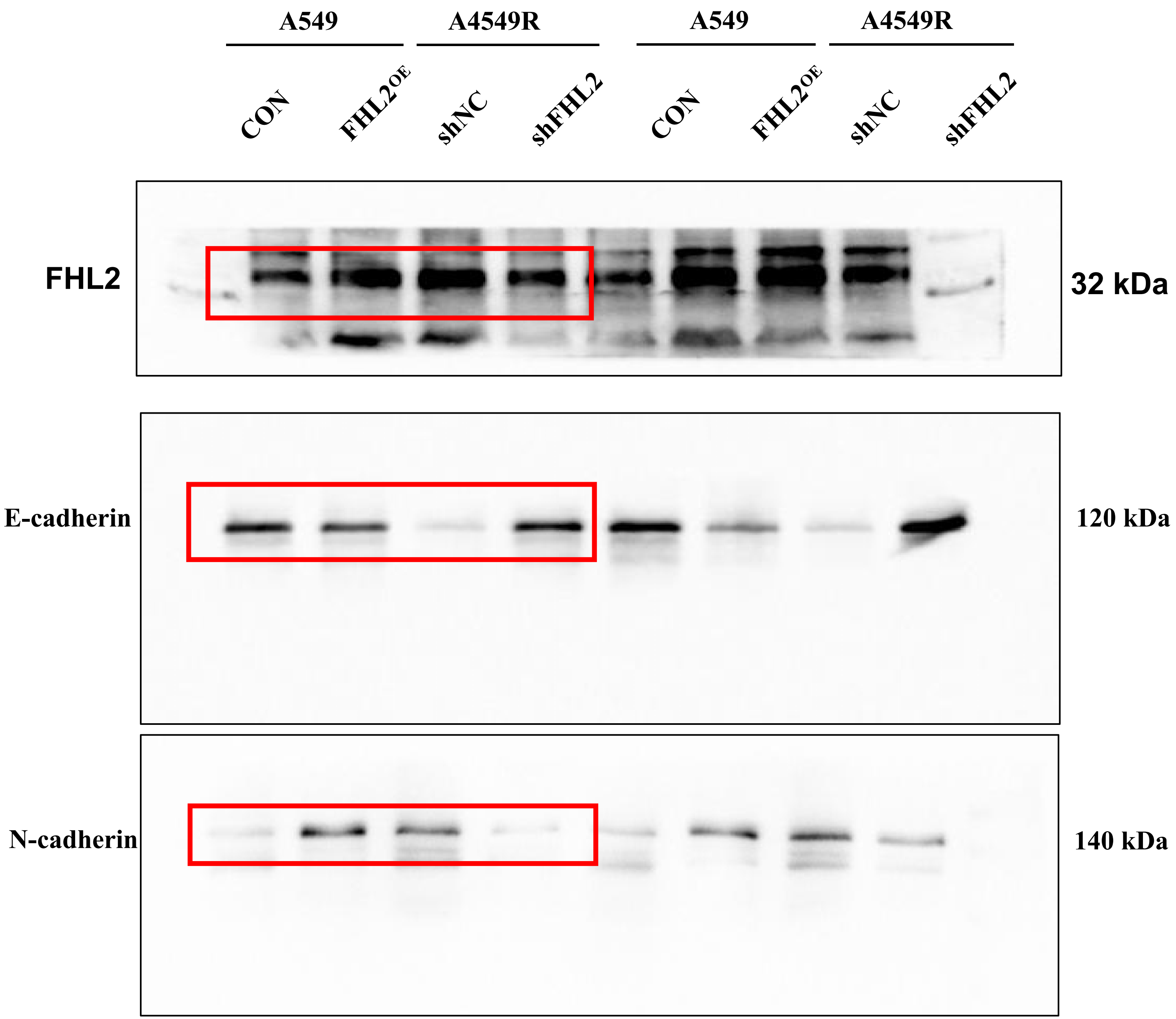

A549
A4549R
A549
A4549R
CON
FHL2OE
shNC
shFHL2
CON
FHL2OE
shNC
shFHL2
FHL2
32 kDa
E-cadherin
120 kDa
N-cadherin
140 kDa

Figure 3D

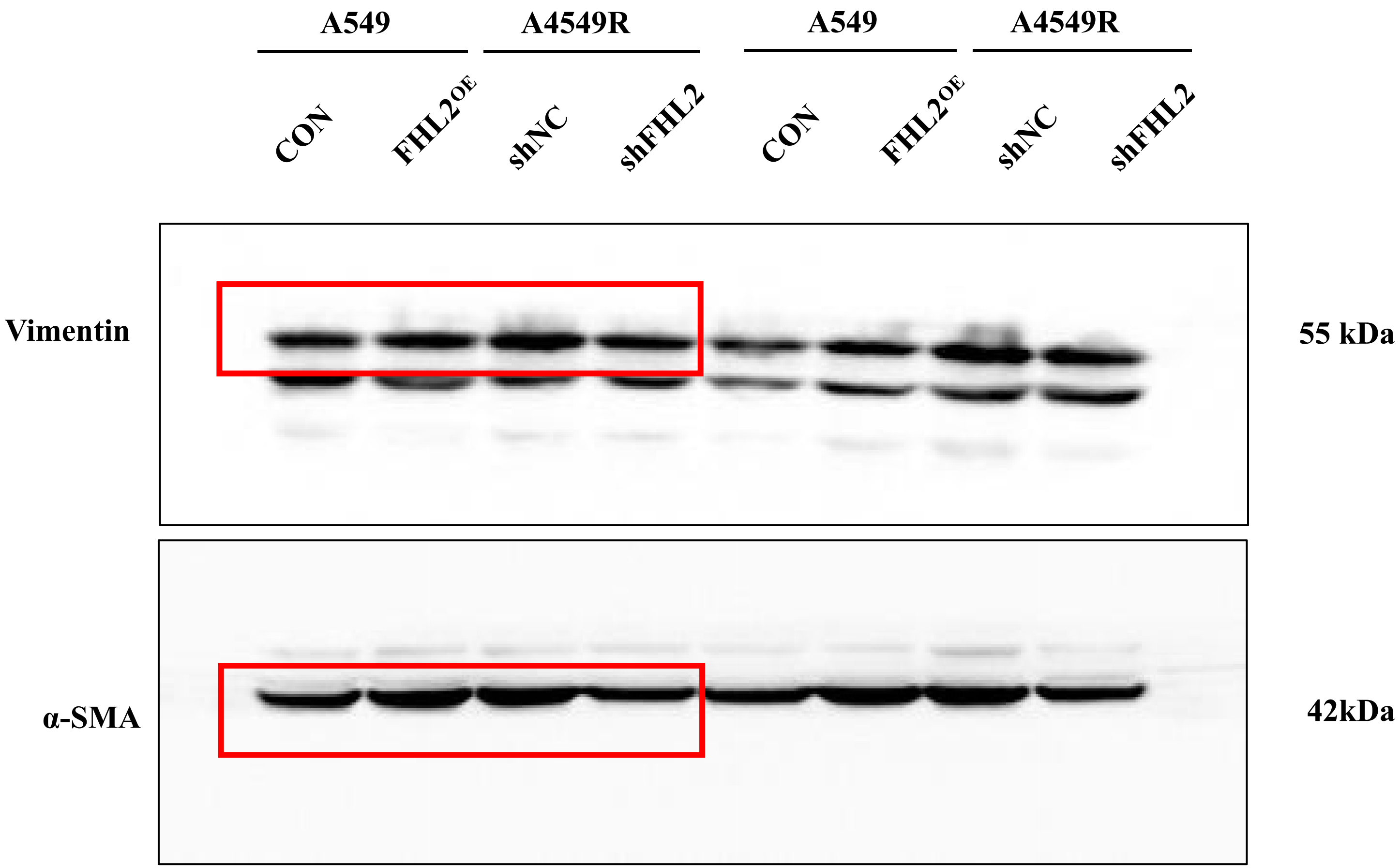

A549
A4549R
A549
A4549R
CON
FHL2OE
shNC
shFHL2
CON
FHL2OE
shNC
shFHL2
Vimentin
55 kDa
α-SMA
42kDa

Figure 3D

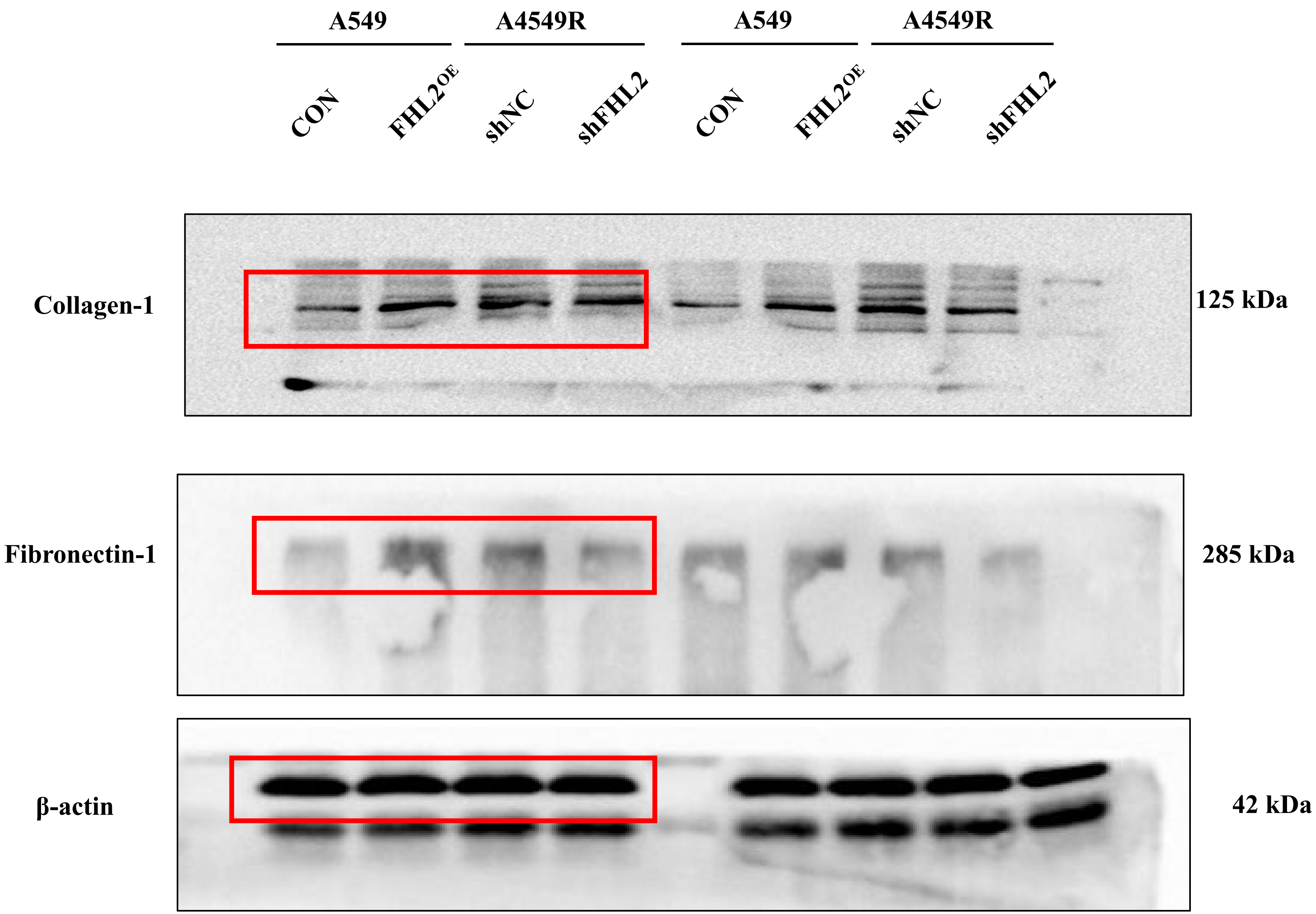
A549
A4549R
A549
A4549R
CON
FHL2OE
shNC
shFHL2
CON
FHL2OE
shNC
shFHL2
Collagen-1
125 kDa
Fibronectin-1
285 kDa
β-actin
42 kDa

Figure 3F

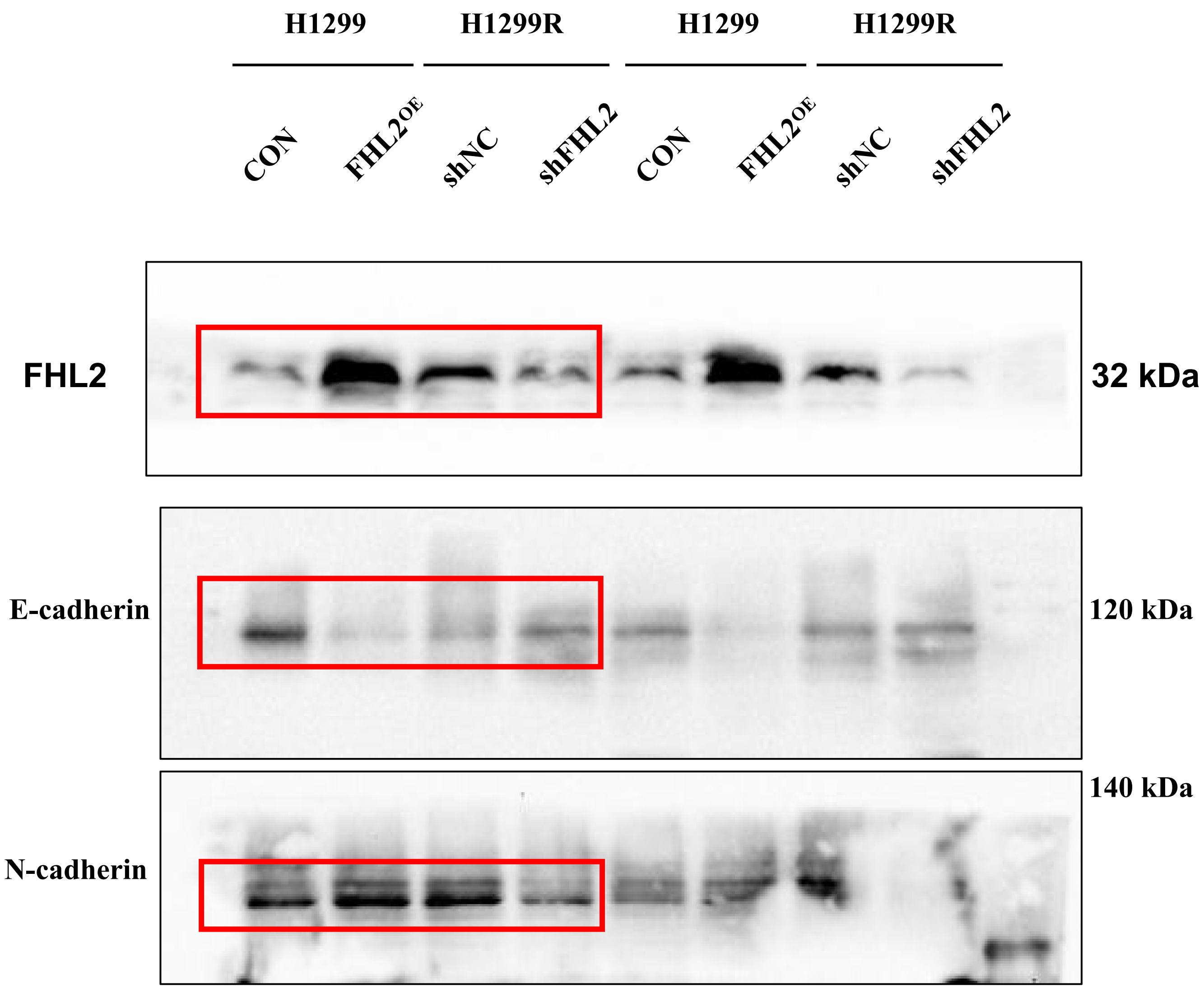

H1299
H1299R
H1299
H1299R
CON
FHL2OE
shNC
shFHL2
CON
FHL2OE
shNC
shFHL2
FHL2
32 kDa
E-cadherin
120 kDa
140 kDa
N-cadherin

Figure 3F

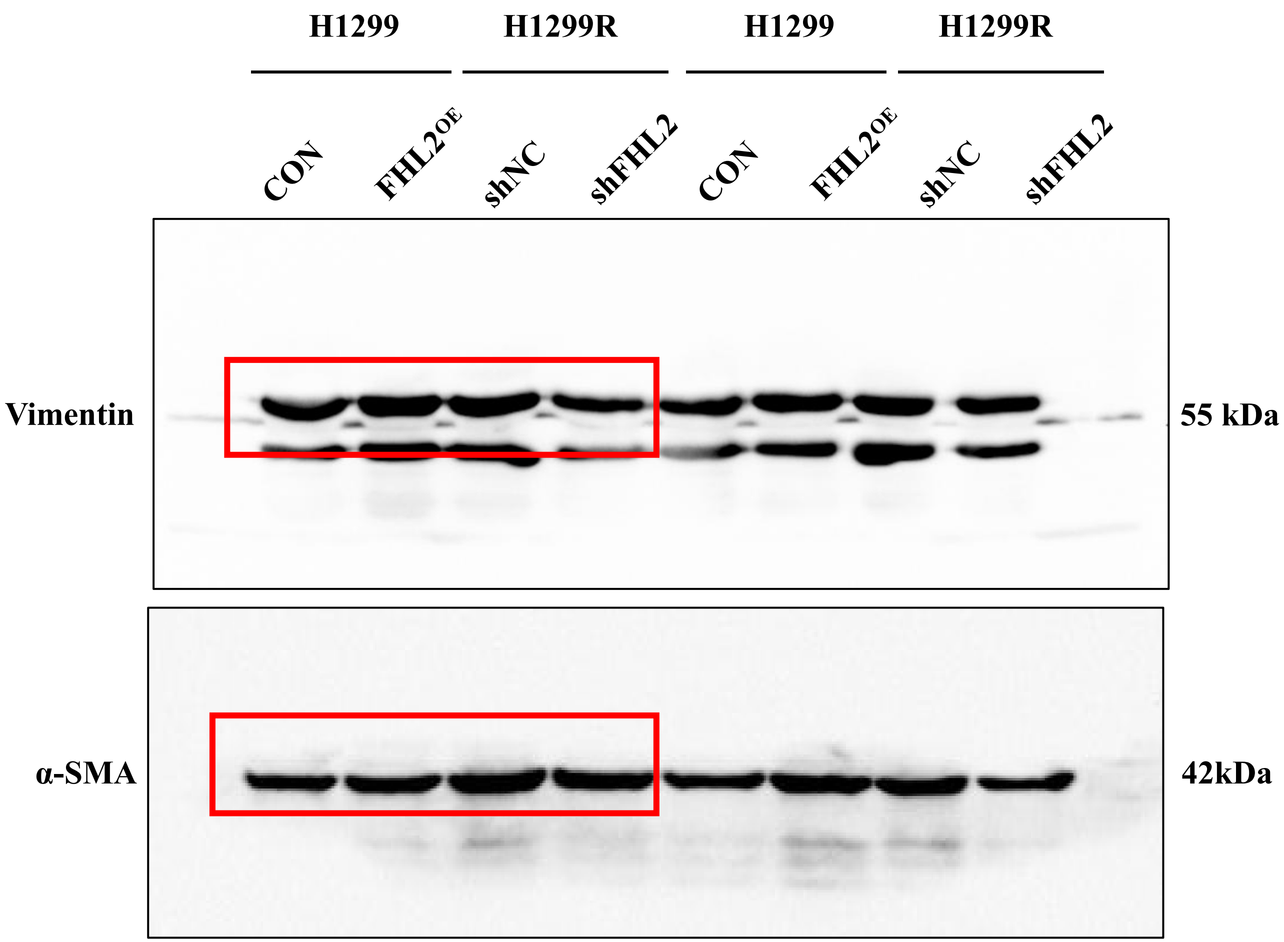

H1299
H1299R
H1299
H1299R
CON
FHL2OE
shNC
shFHL2
CON
FHL2OE
shNC
shFHL2
Vimentin
55 kDa
α-SMA
42kDa

Figure 3F

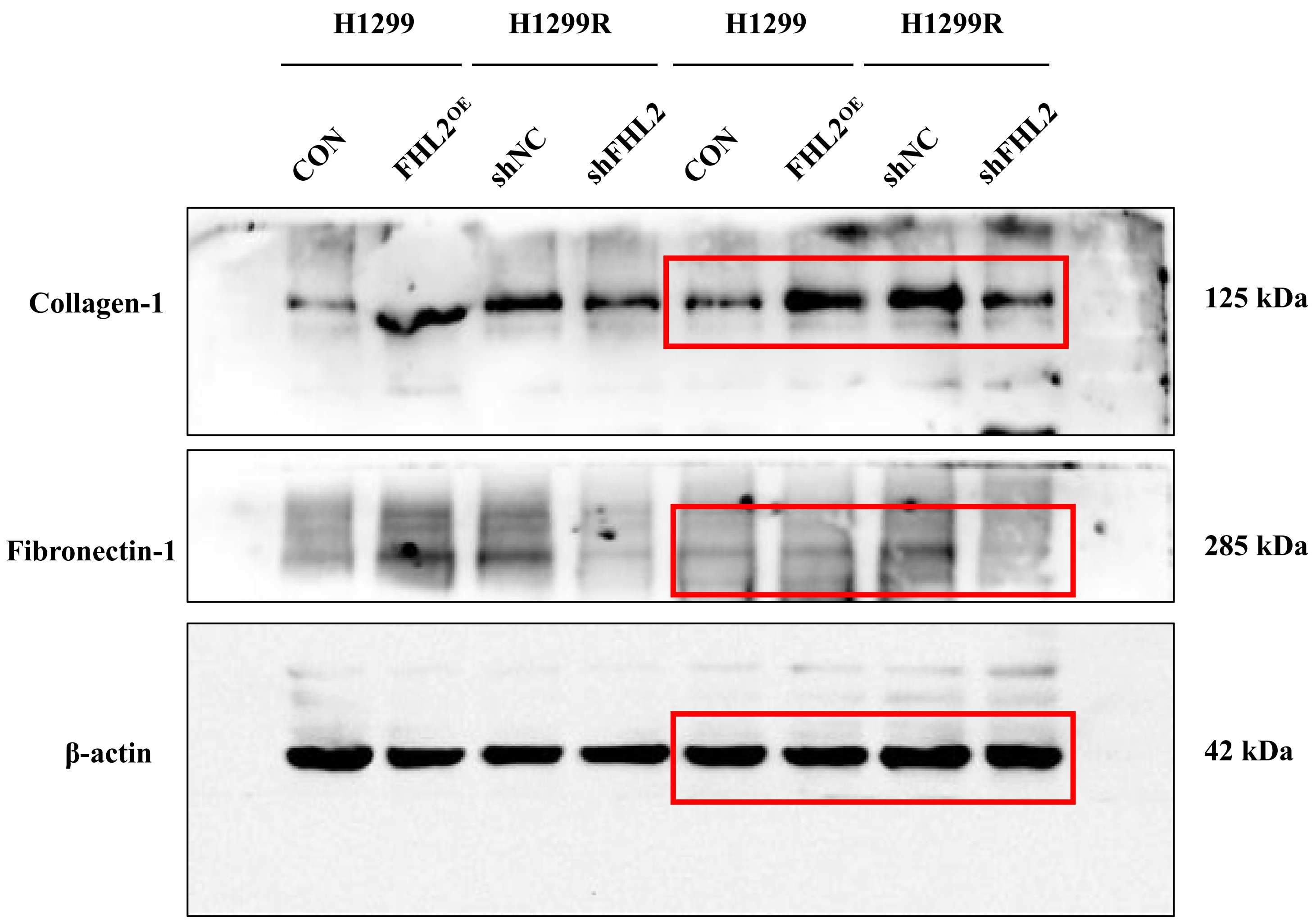
H1299
H1299R
H1299
H1299R
CON
FHL2OE
shNC
shFHL2
CON
FHL2OE
shNC
shFHL2
Collagen-1
125 kDa
Fibronectin-1
285 kDa
β-actin
42 kDa

Figure 5E

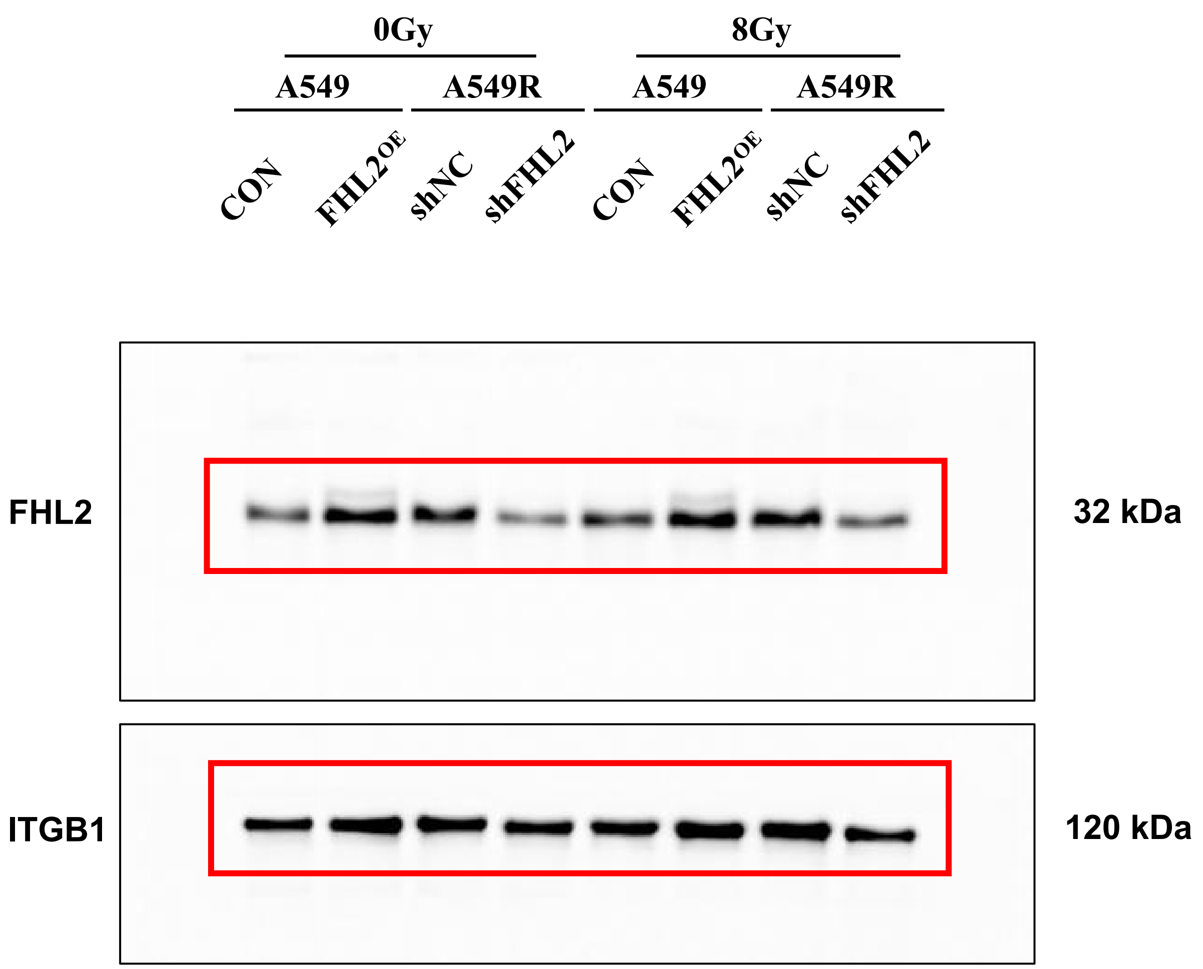
0Gy
8Gy
A549
A549R
A549
A549R
CON
FHL2OE
shNC
shFHL2
CON
FHL2OE
shNC
shFHL2
FHL2
32 kDa
ITGB1
120 kDa

Figure 4E

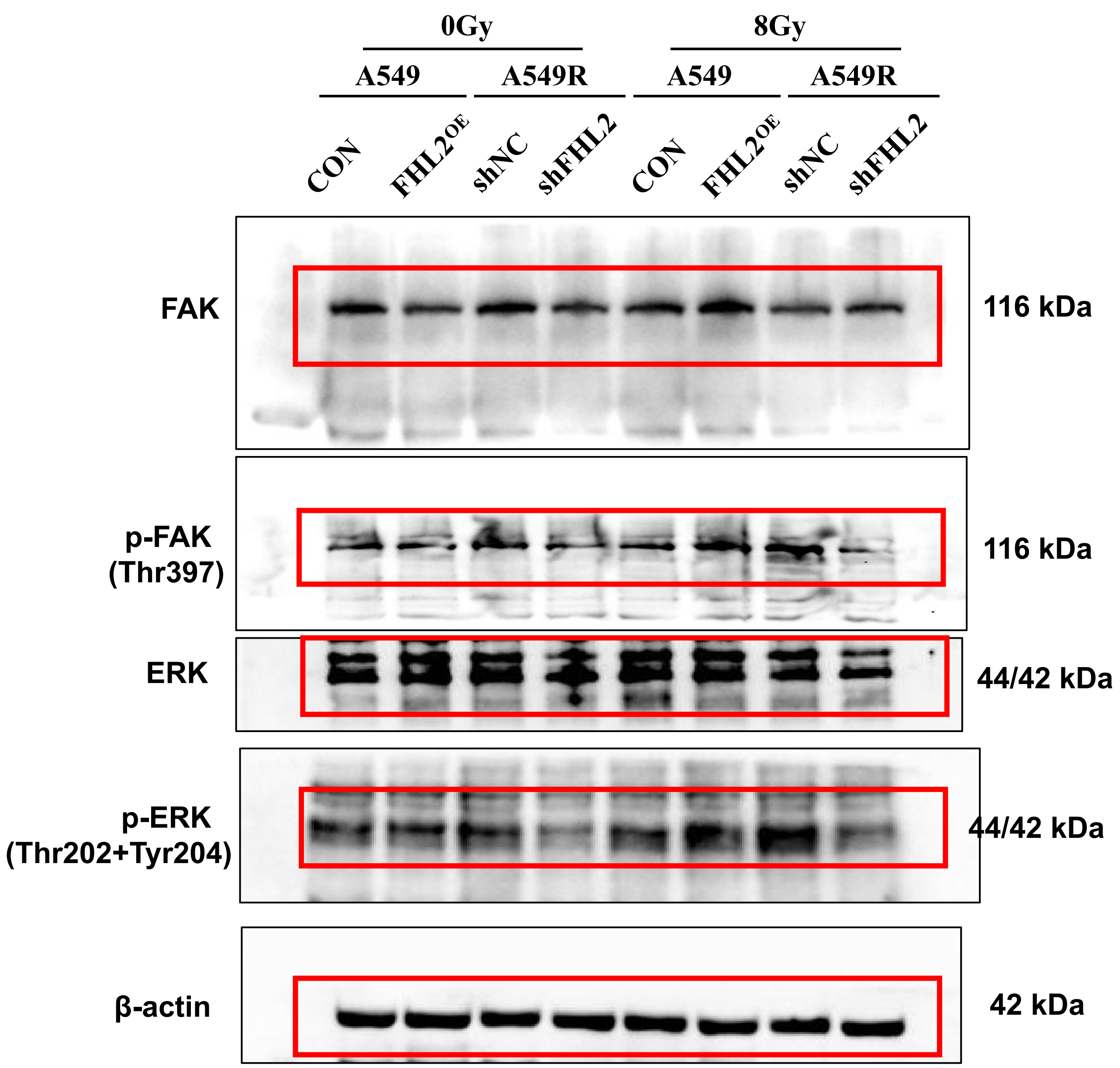
0Gy
8Gy
A549
A549R
A549
A549R
CON
FHL2OE
shNC
shFHL2
CON
FHL2OE
shNC
shFHL2
FAK
116 kDa
p-FAK
(Thr397)
116 kDa
ERK
44/42 kDa
p-ERK
(Thr202+Tyr204)
44/42 kDa
β-actin
42 kDa

Figure 4F

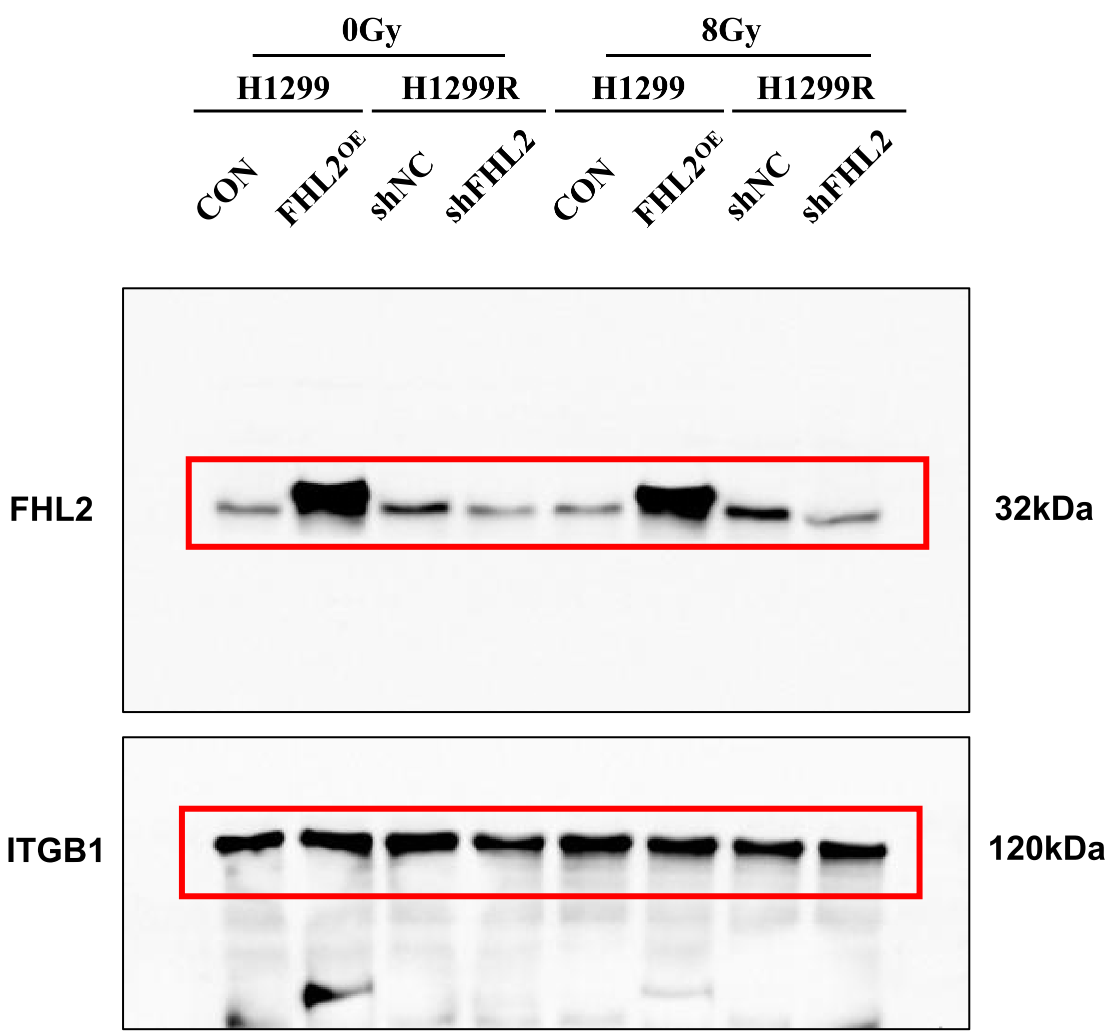
0Gy
8Gy
H1299
H1299R
H1299
H1299R
CON
FHL2OE
shNC
shFHL2
CON
FHL2OE
shNC
shFHL2
FHL2
32kDa
ITGB1
120kDa

Figure 4F

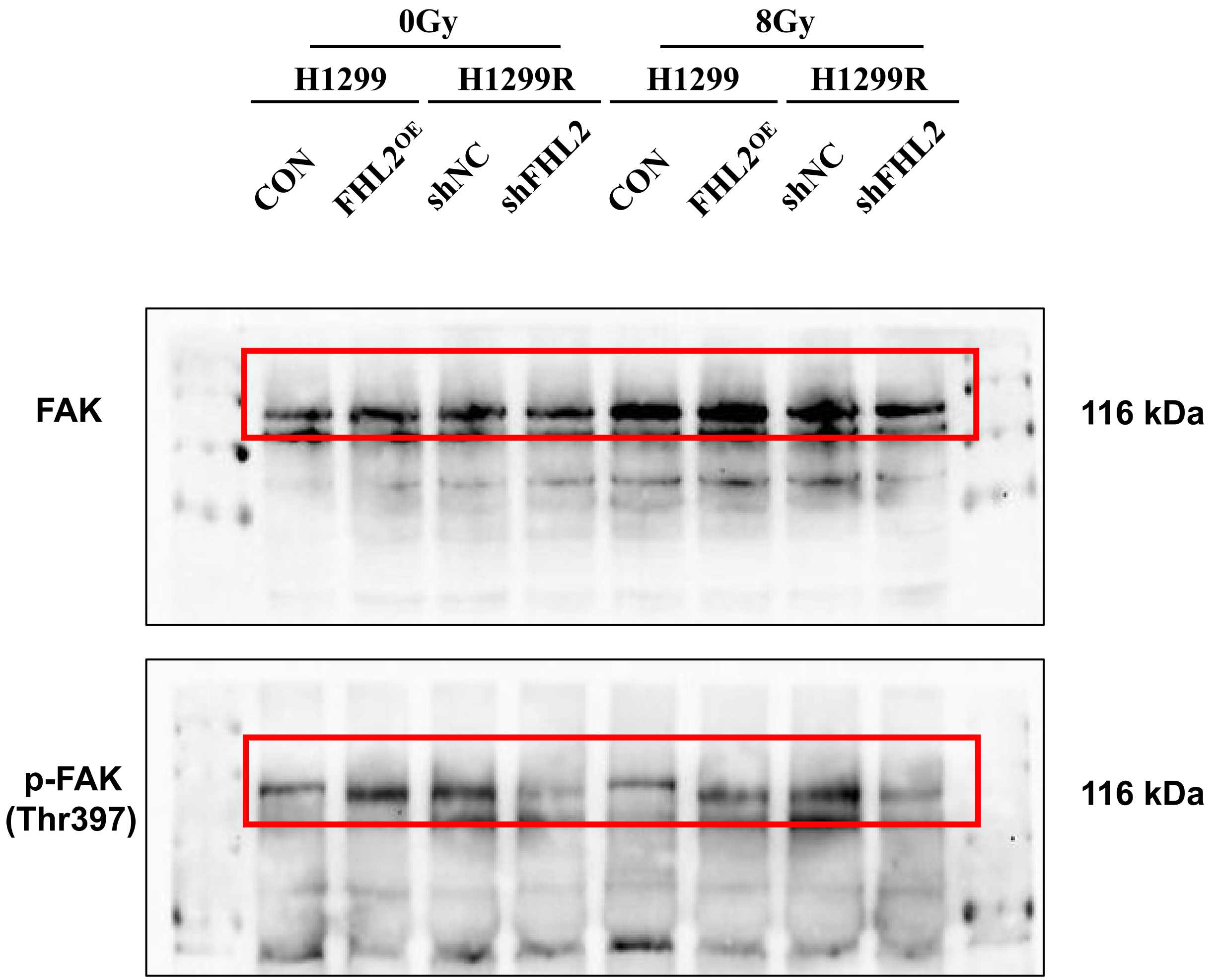
0Gy
8Gy
H1299
H1299R
H1299
H1299R
CON
FHL2OE
shNC
shFHL2
CON
FHL2OE
shNC
shFHL2
FAK
116 kDa
p-FAK
(Thr397)
116 kDa

Figure 4F

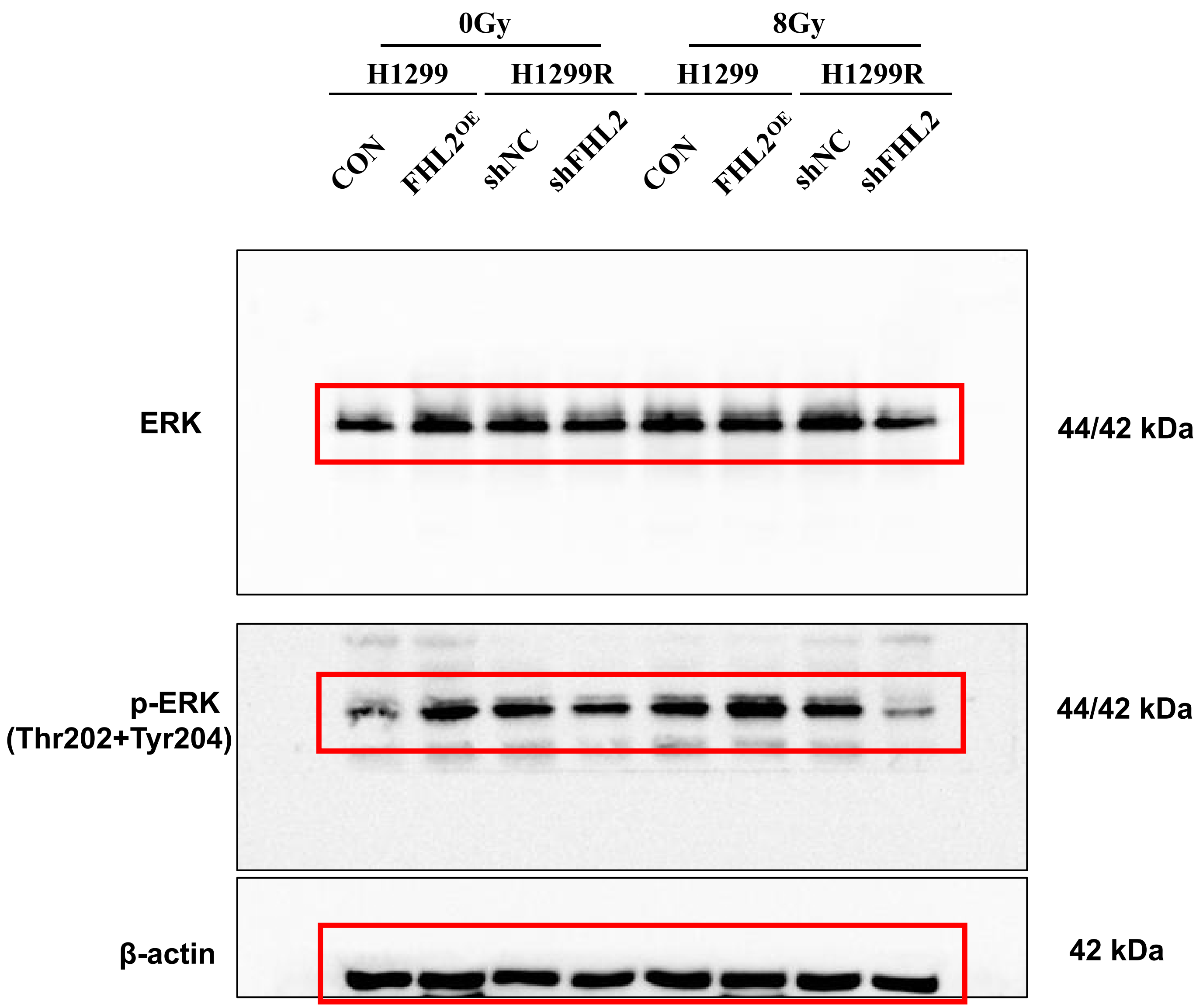
0Gy
8Gy
H1299
H1299R
H1299
H1299R
CON
FHL2OE
shNC
shFHL2
CON
FHL2OE
shNC
shFHL2
ERK
44/42 kDa
p-ERK
(Thr202+Tyr204)
44/42 kDa
β-actin
42 kDa

Figure 4J

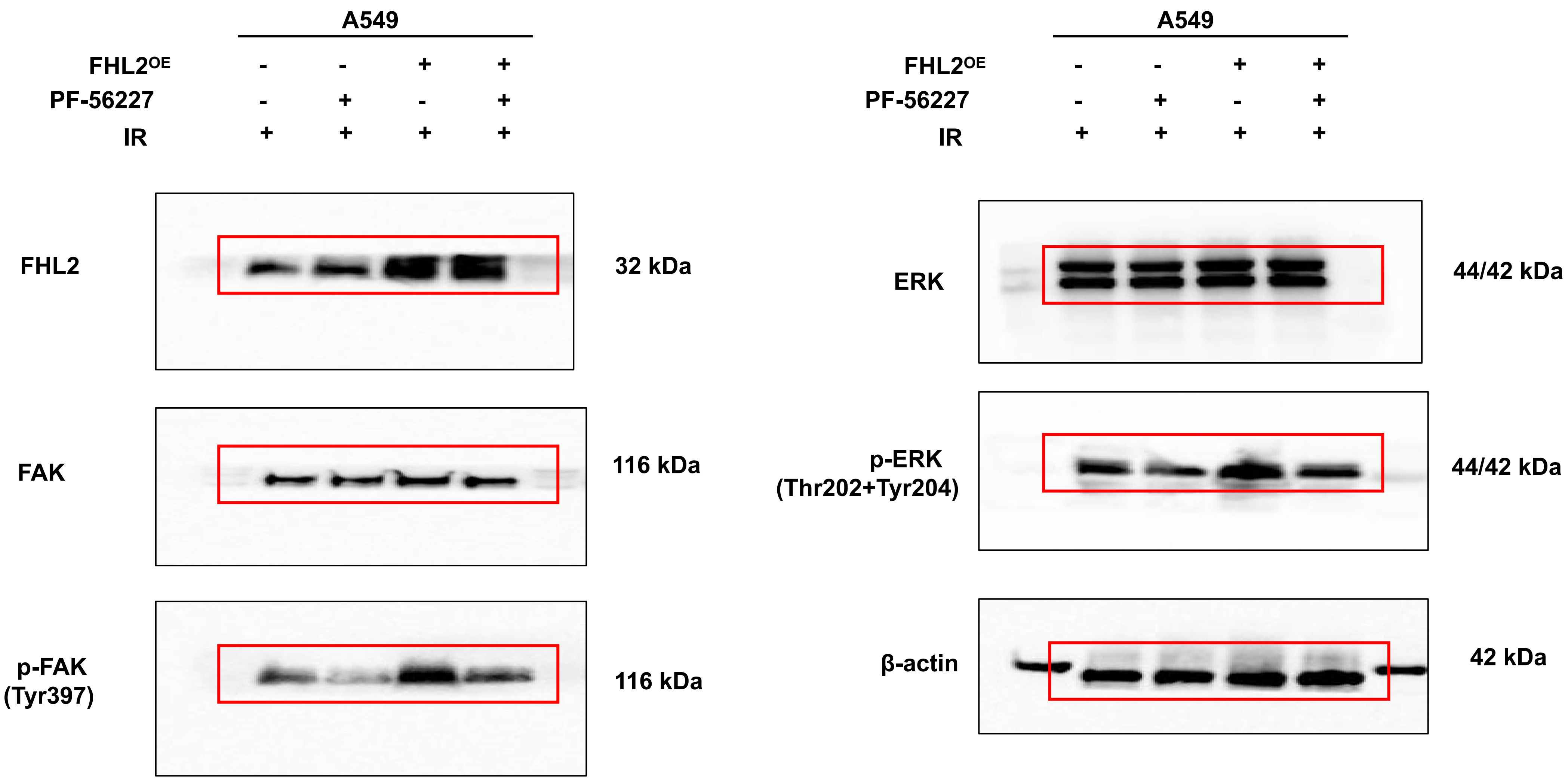
A549
FHL2OE - - + +
PF-56227 - + - +
IR + + + +
FHL2
32 kDa
FAK
116 kDa
p-FAK
(Tyr397)
116 kDa
A549
FHL2OE - - + +
PF-56227 - + - +
IR + + + +
ERK
44/42 kDa
p-ERK
(Thr202+Tyr204)
44/42 kDa
β-actin
42 kDa

Figure 4J

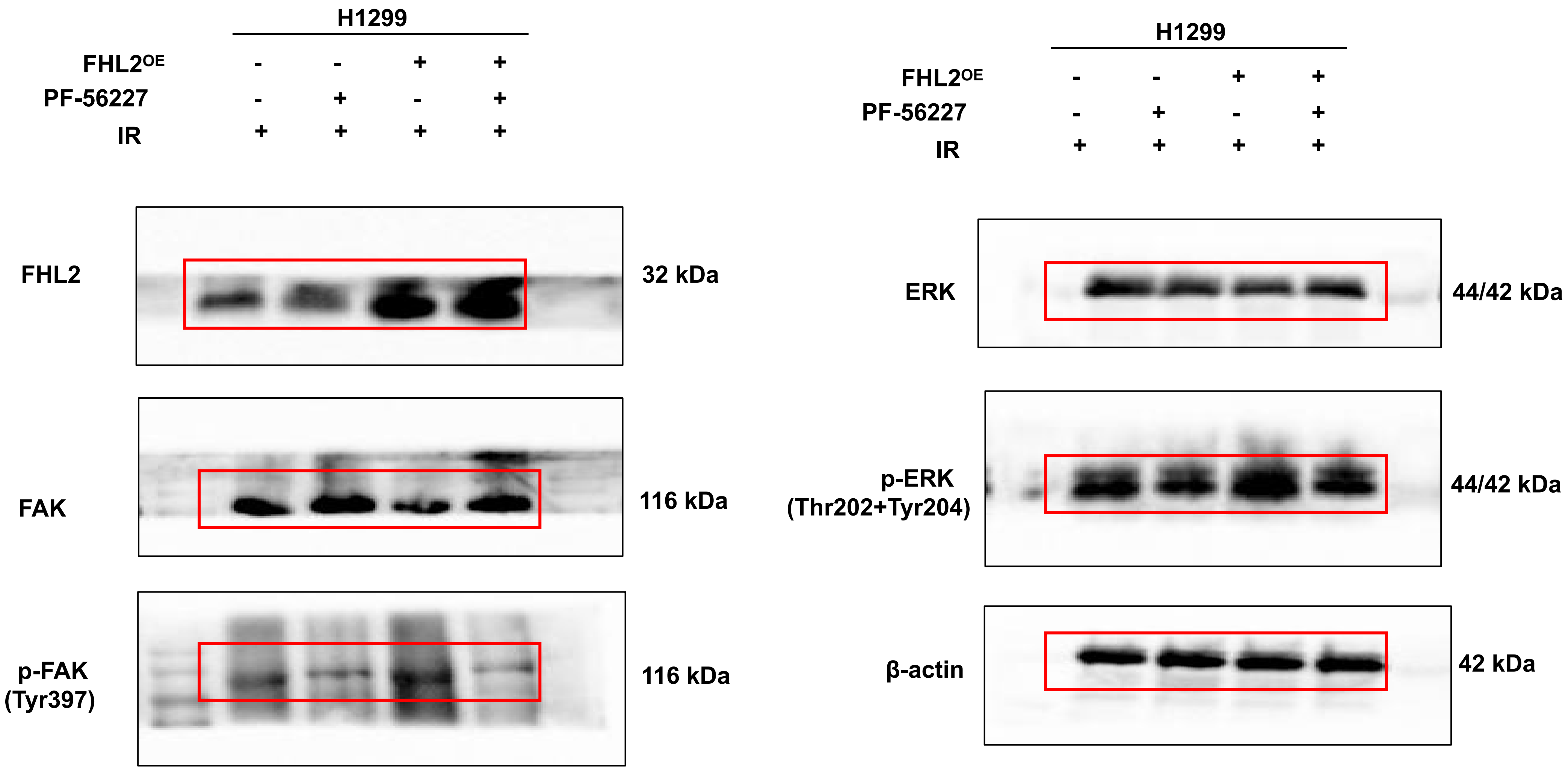
H1299
FHL2OE - - + +
PF-56227 - + - +
IR + + + +
FHL2
32 kDa
FAK
116 kDa
p-FAK
(Tyr397)
116 kDa
H1299
FHL2OE - - + +
PF-56227 - + - +
IR + + + +
ERK
44/42 kDa
p-ERK
(Thr202+Tyr204)
44/42 kDa
β-actin
42 kDa

Figure 5A

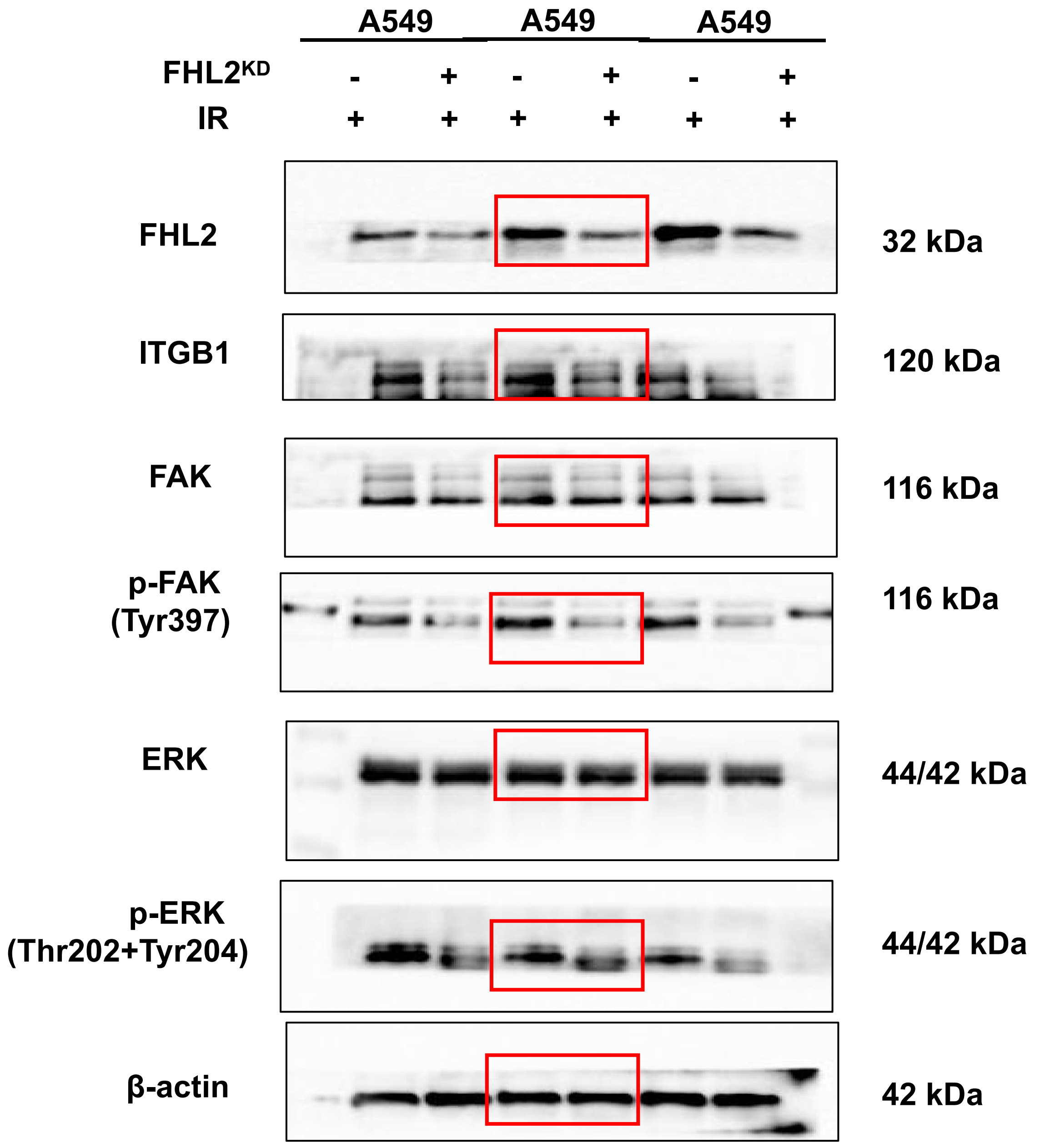

A549
A549
A549
FHL2KD
IR
- + - + - +
+ + + + + +
FHL2
ITGB1
FAK
p-FAK
(Tyr397)
ERK
p-ERK
(Thr202+Tyr204)
β-actin
32 kDa
120 kDa
116 kDa
116 kDa
44/42 kDa
44/42 kDa
42 kDa

Figure 5A

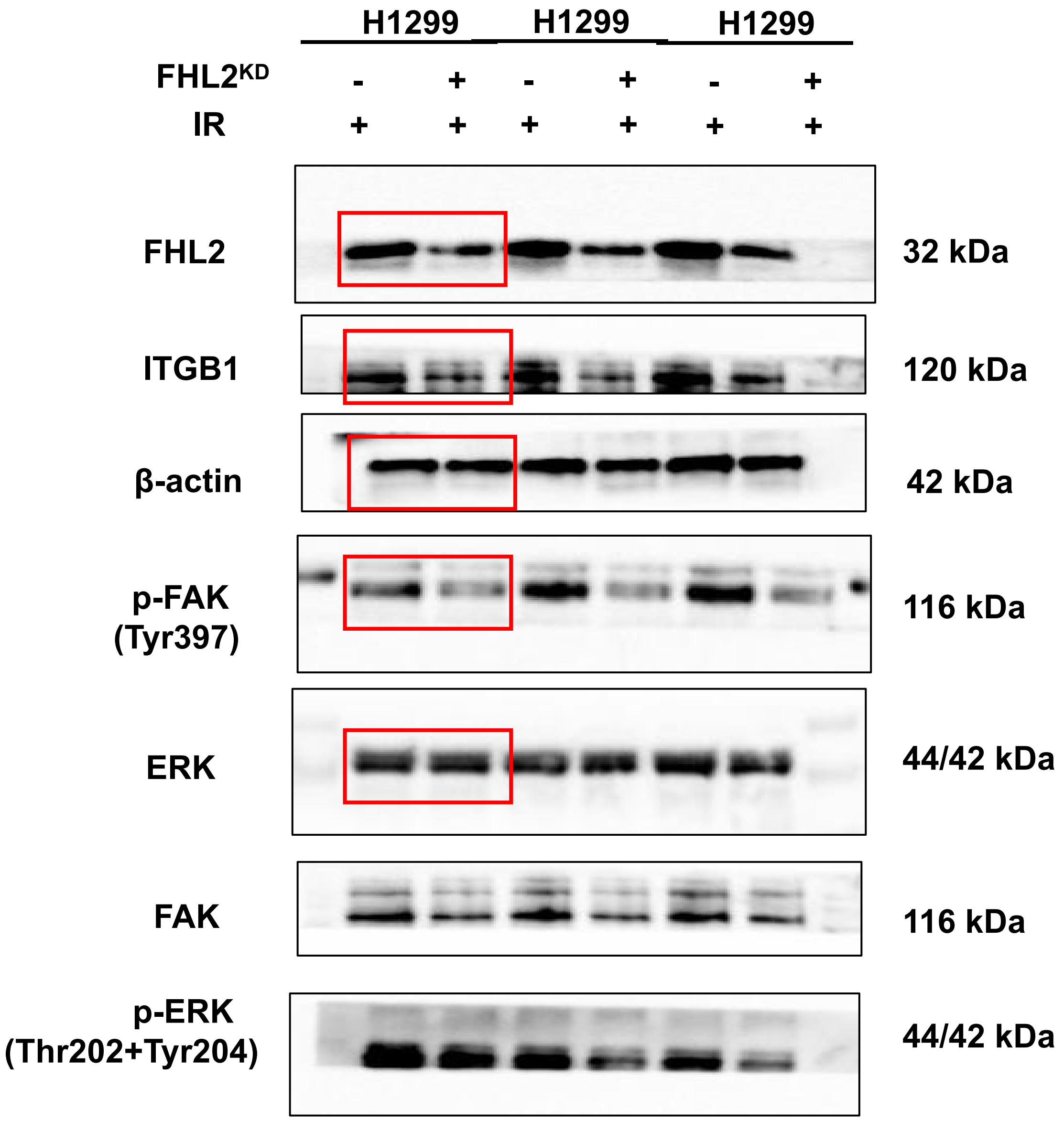
H1299
H1299
H1299
FHL2KD
IR
- + - + - +
+ + + + + +
FHL2
32 kDa
ITGB1
120 kDa
β-actin
42 kDa
p-FAK
(Tyr397)
116 kDa
ERK
44/42 kDa
FAK
116 kDa
p-ERK
(Thr202+Tyr204)
44/42 kDa

Figure 5H

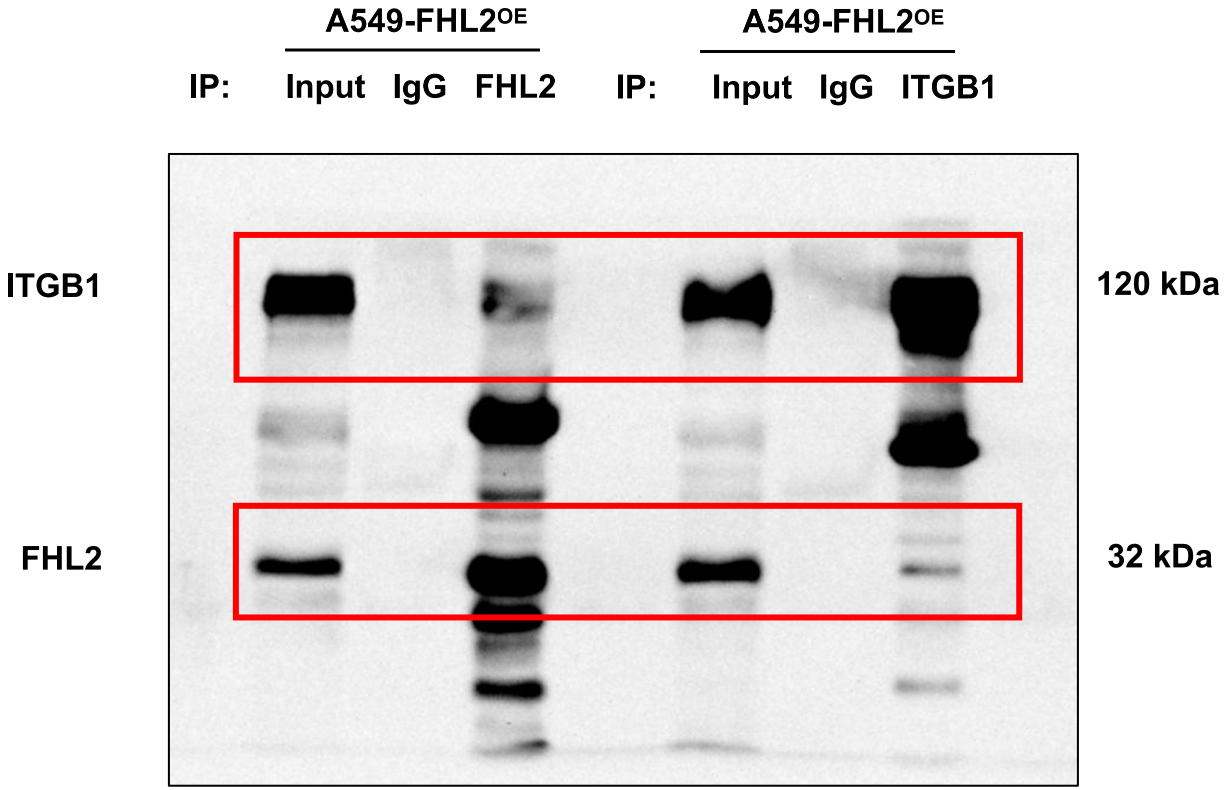
A549-FHL2^{OE}
A549-FHL2^{OE}
IP: Input IgG FHL2
IP: Input IgG ITGB1
ITGB1
FHL2
120 kDa
32 kDa

Figure 5H

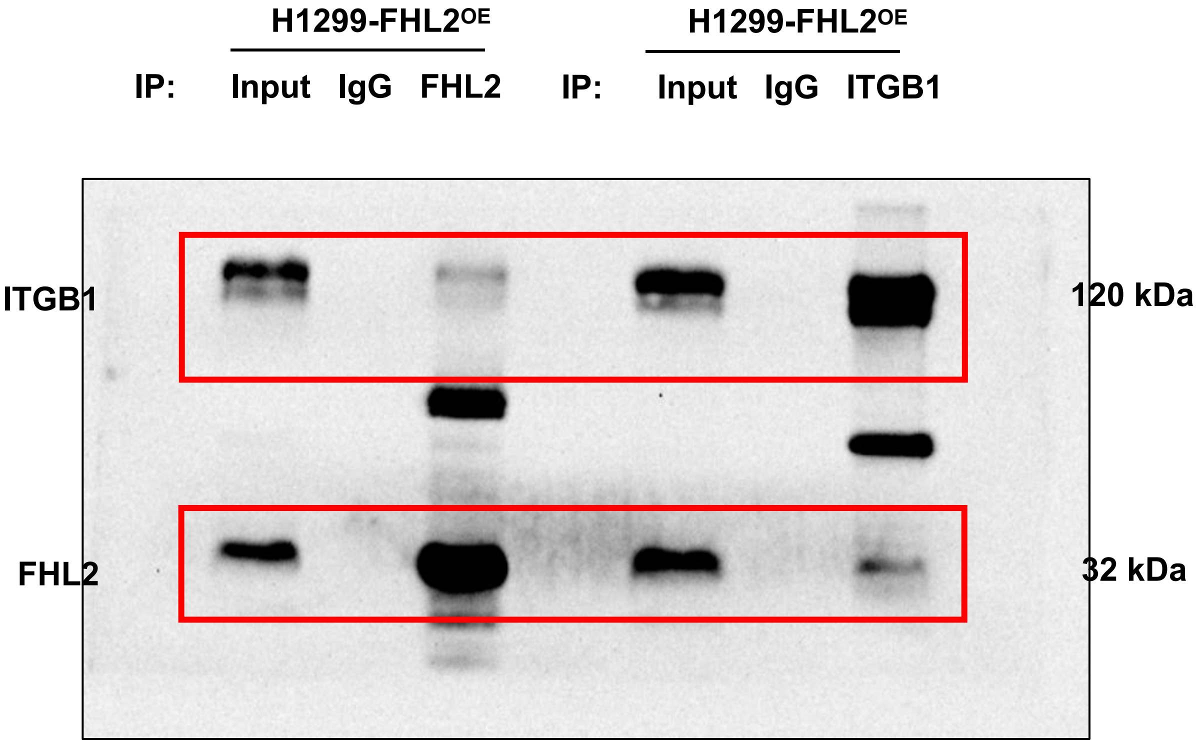
H1299-FHL2OE
H1299-FHL2OE
IP:
Input
IgG
FHL2
IP:
Input
IgG
ITGB1
ITGB1
FHL2
120 kDa
32 kDa

Figure 5I

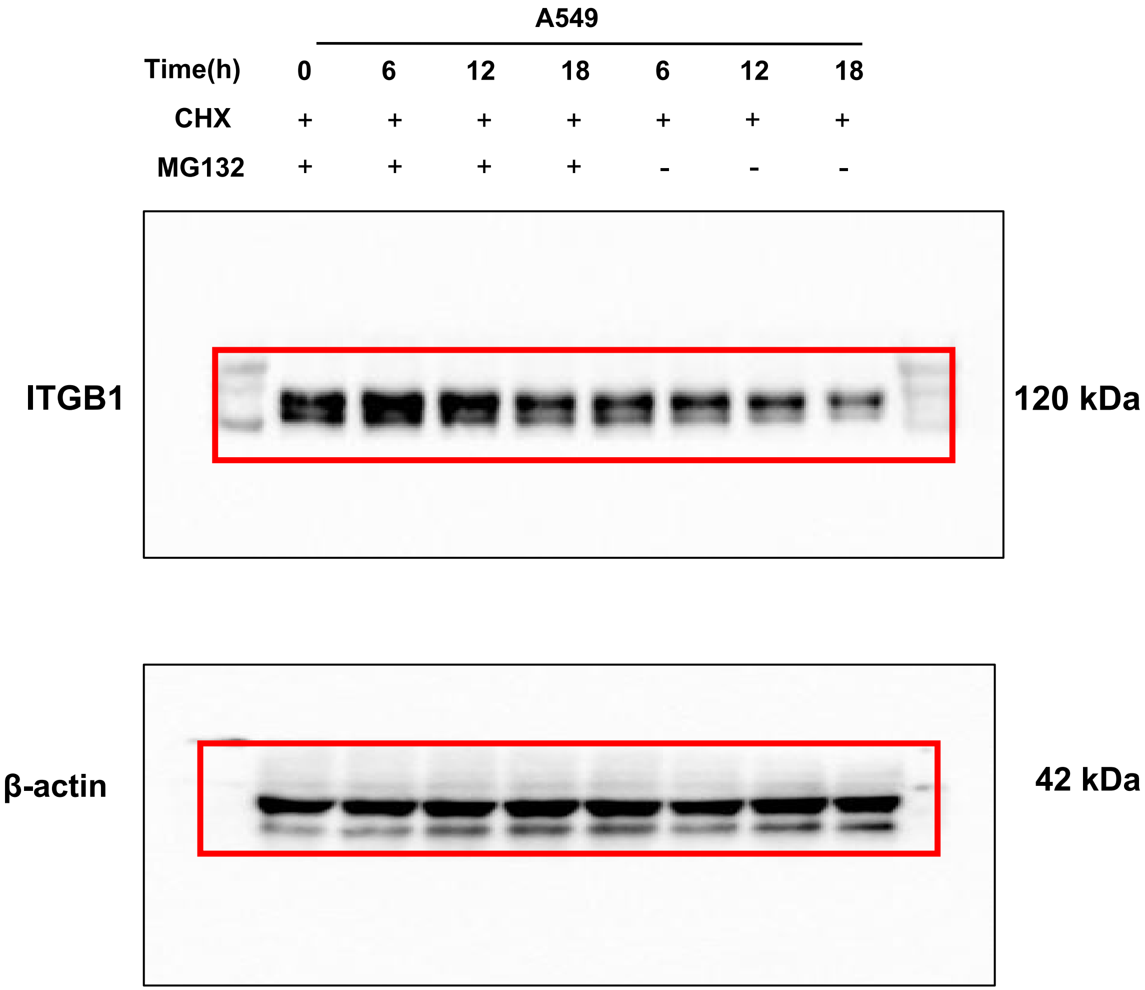
A549
Time(h) 0 6 12 18 6 12 18
CHX + + + + + + +
MG132 + + + + - - -
ITGB1
120 kDa
β-actin
42 kDa

Figure 5J

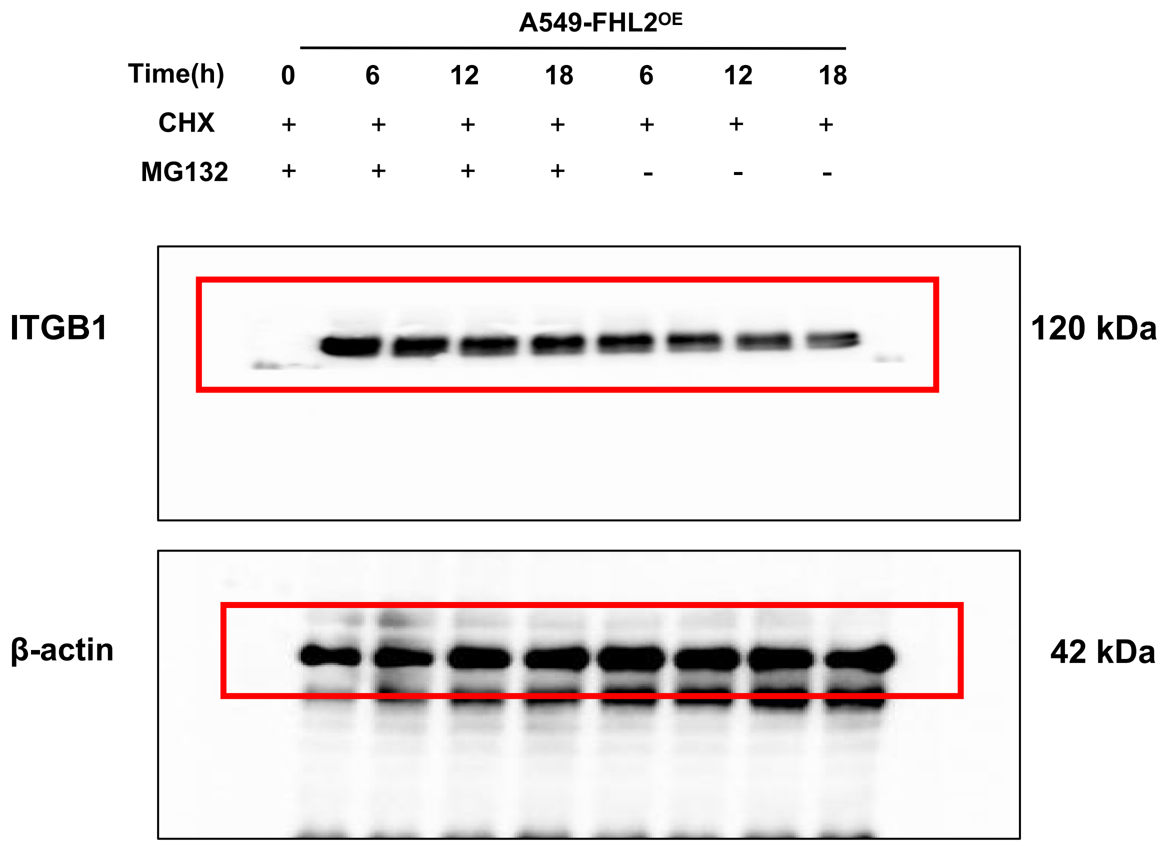
A549-FHL2OE
Time(h) 0 6 12 18 6 12 18
CHX + + + + + + +
MG132 + + + + - - -
ITGB1
120 kDa
β-actin
42 kDa

Figure 5K

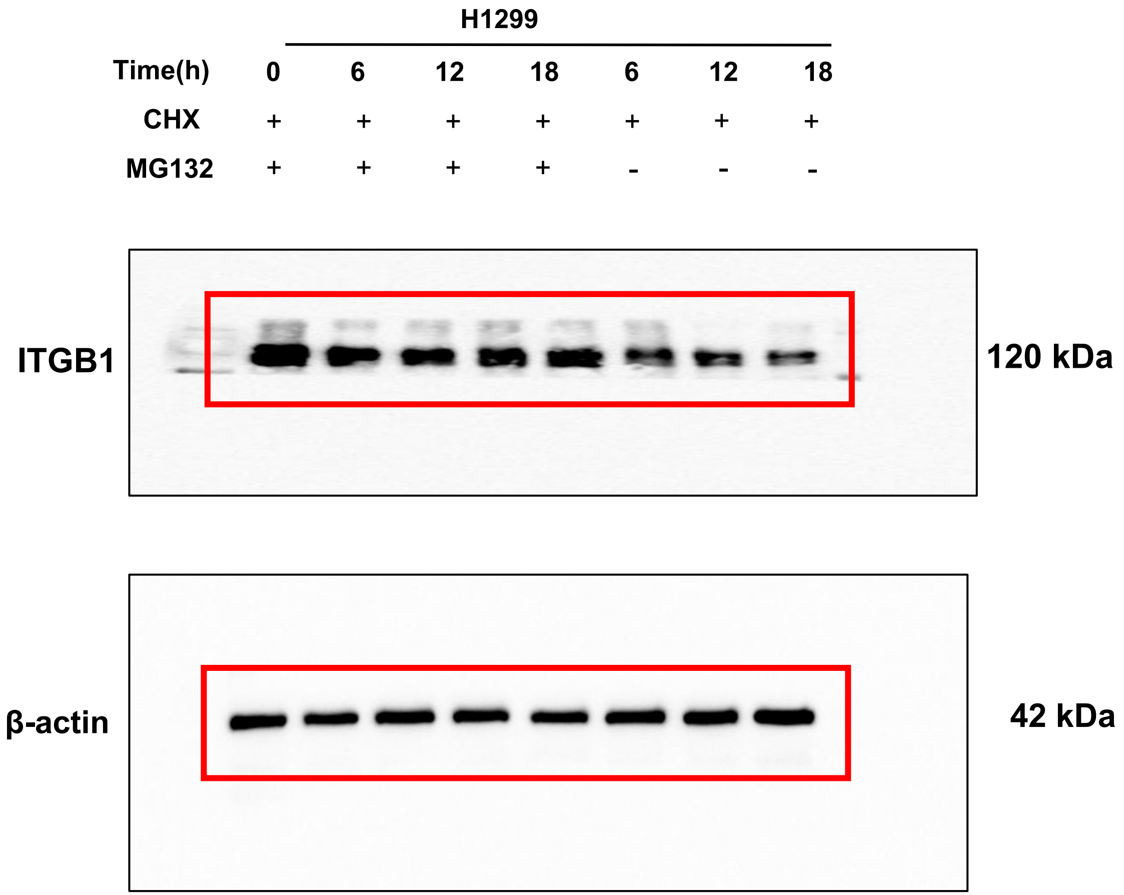
H1299
Time(h) 0 6 12 18 6 12 18
CHX + + + + + + +
MG132 + + + + - - -
ITGB1
120 kDa
β-actin
42 kDa

Figure 5L

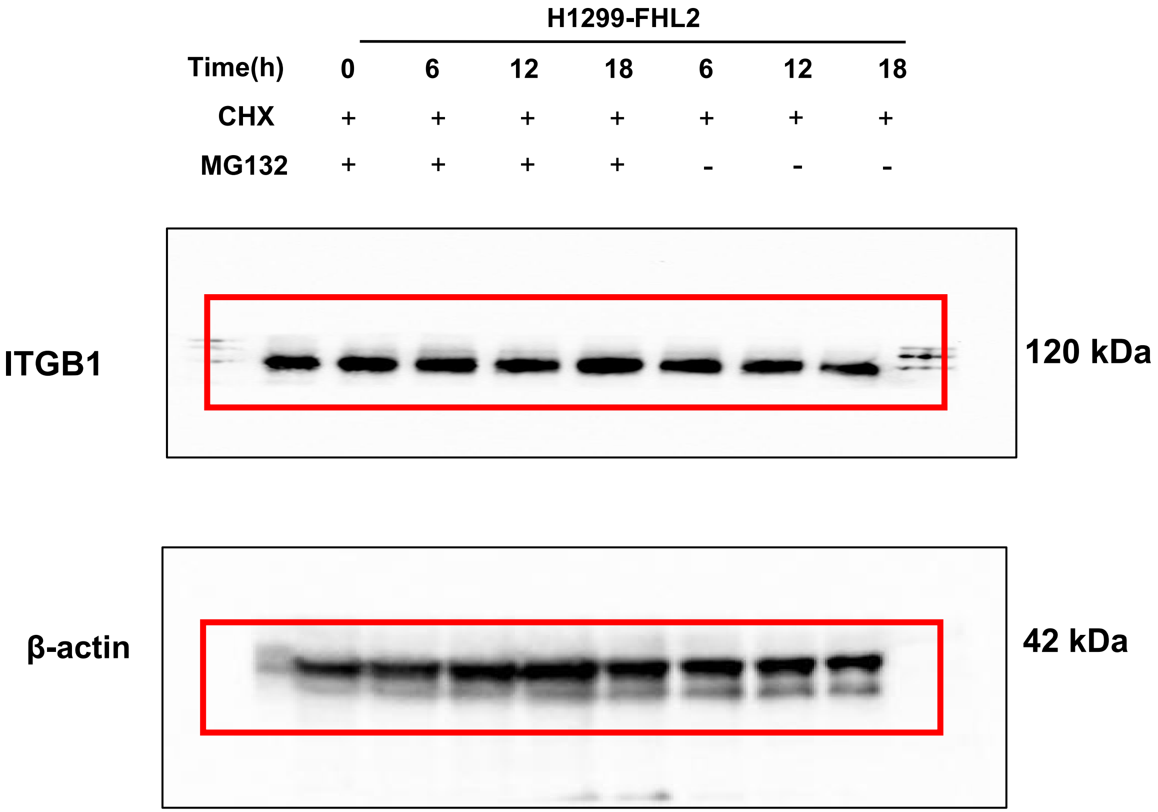

H1299-FHL2
Time(h) 0 6 12 18 6 12 18
CHX + + + + + + +
MG132 + + + + - - -
ITGB1
120 kDa
β-actin
42 kDa

Figure 5N

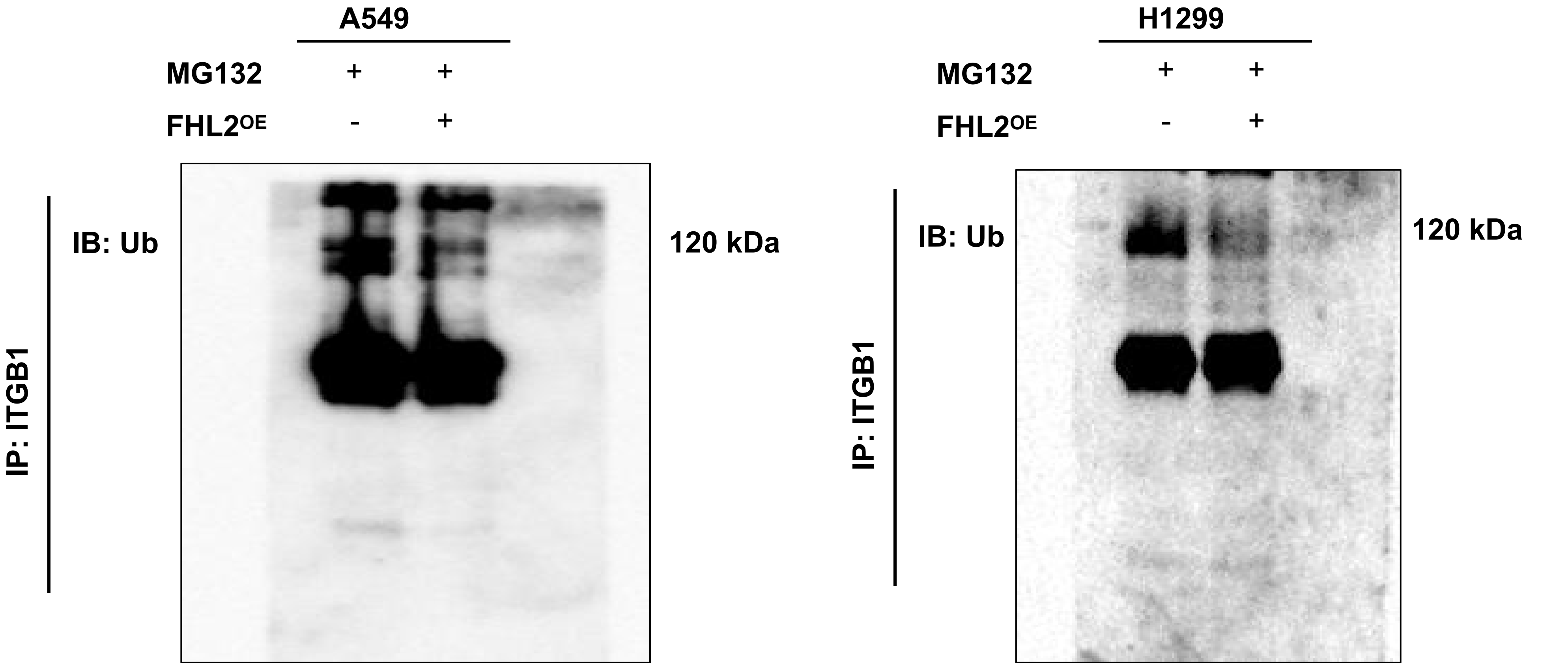
A549
MG132 + +
FHL2OE - +
IB: Ub
120 kDa
IP: ITGB1
H1299
MG132 + +
FHL2OE - +
IB: Ub
120 kDa
IP: ITGB1

Figure 5N

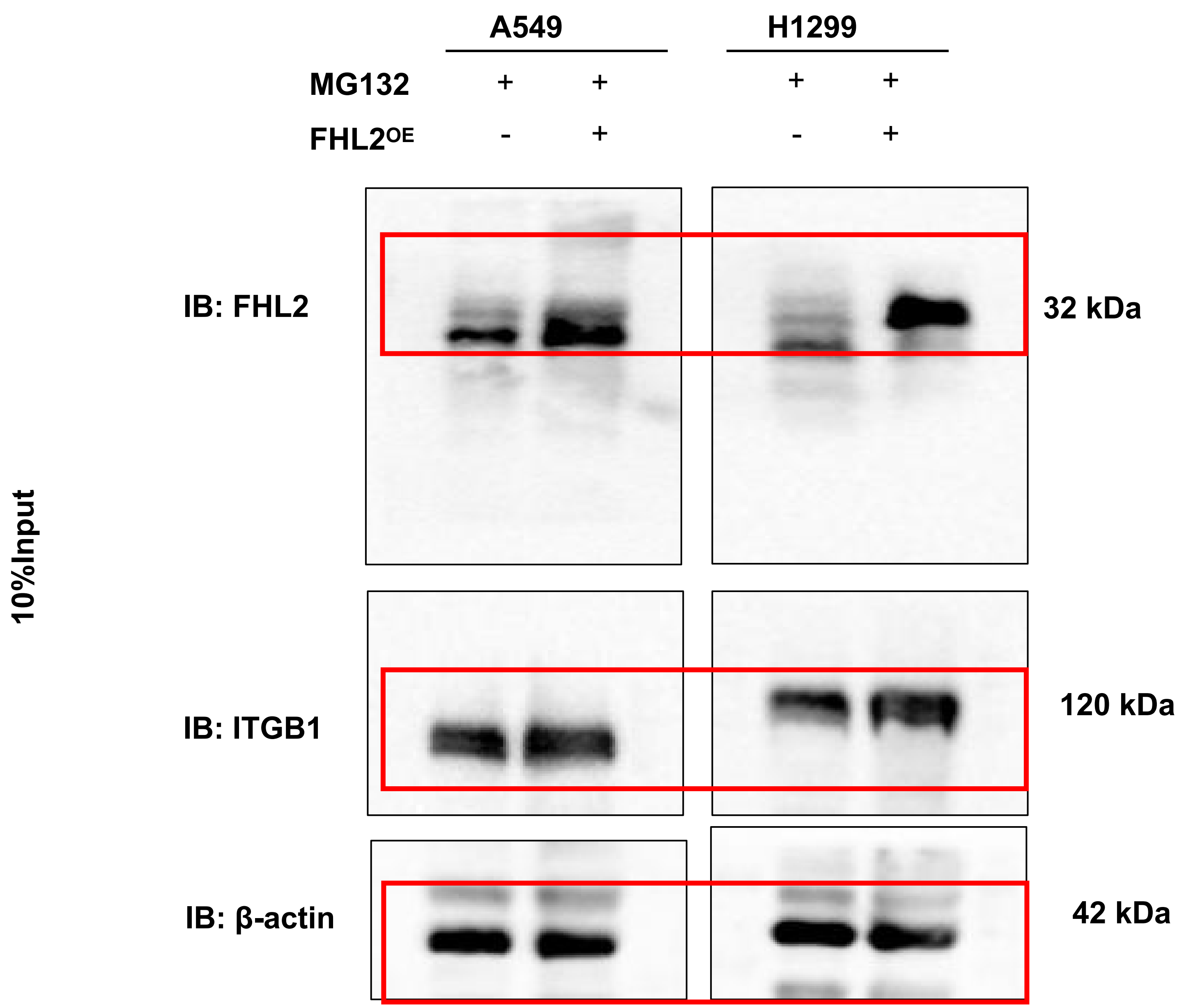
A549
H1299
MG132
+
+
+
+
FHL2OE
-
+
-
+
10%Input
IB: FHL2
32 kDa
IB: ITGB1
120 kDa
IB: β-actin
42 kDa

Figure 5O

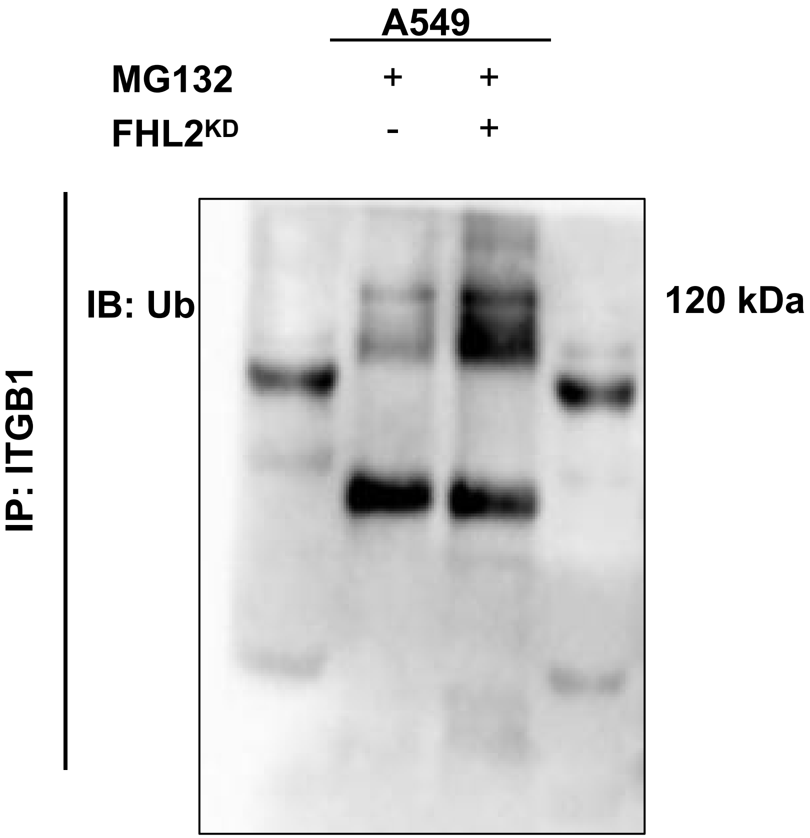
A549
MG132
+
+
FHL2KD
-
+
IB: Ub
120 kDa
IP: ITGB1

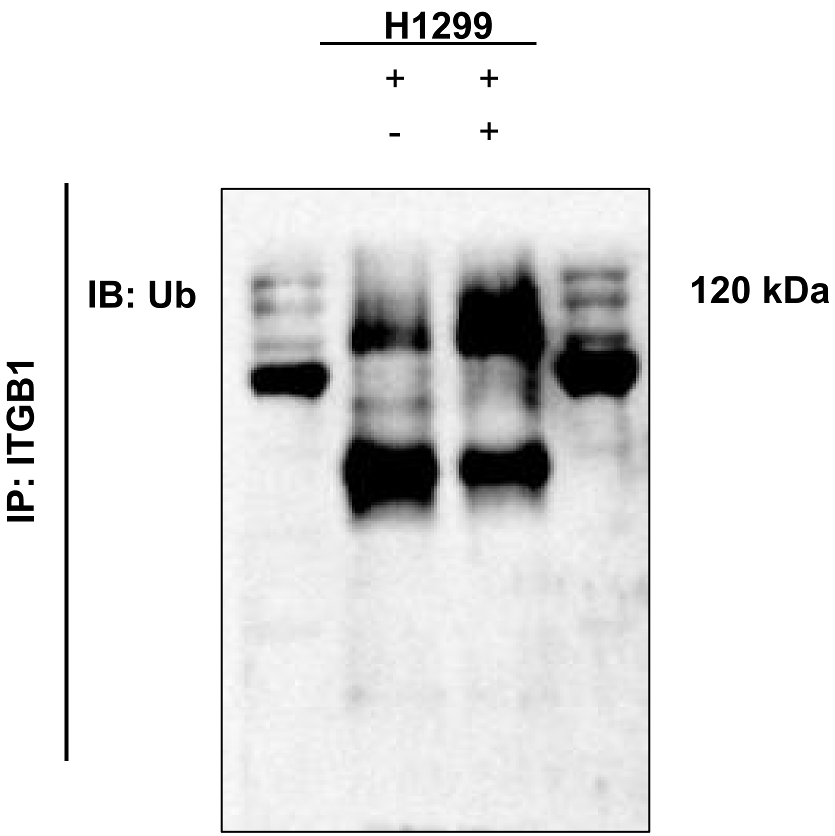
H1299
+
+
-
+
IB: Ub
120 kDa
IP: ITGB1

Figure 5O

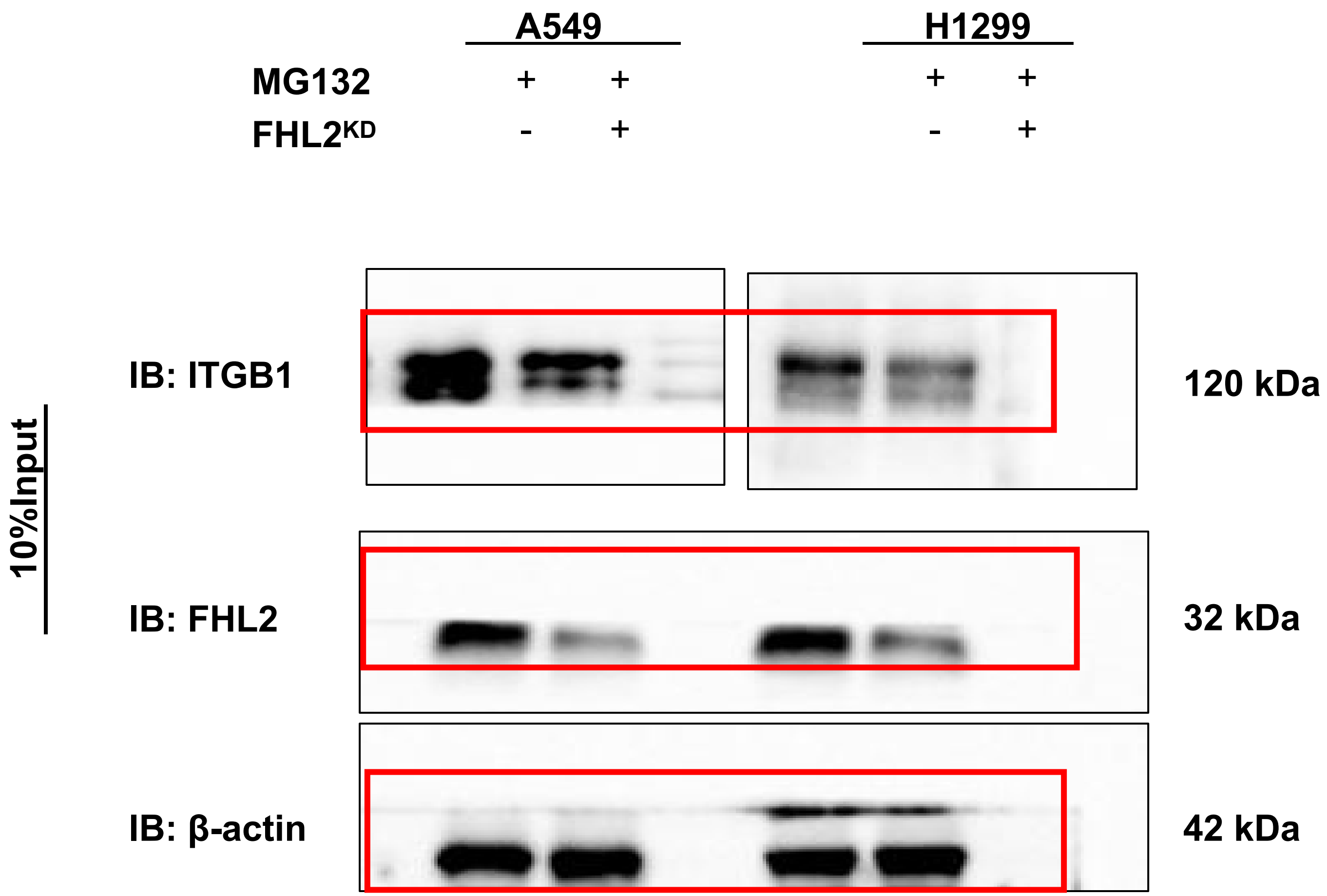

A549
H1299
MG132
+
+
+
+
FHL2KD
-
+
-
+
10%Input
IB: ITGB1
120 kDa
IB: FHL2
32 kDa
IB: β-actin
42 kDa

Figure 5P

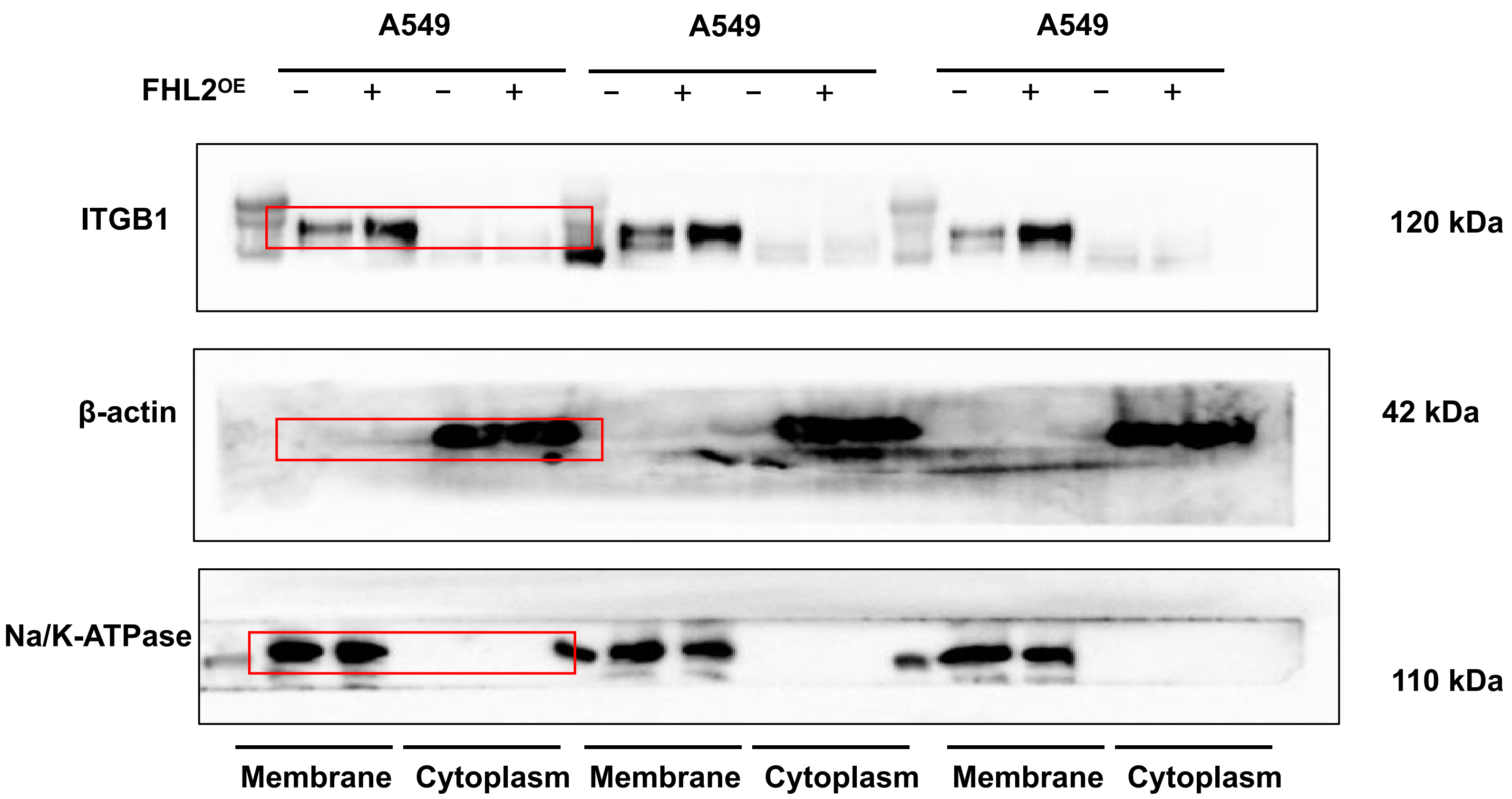
A549
A549
A549
FHL2OE
− + − +
− + − +
− + − +
ITGB1
120 kDa
β-actin
42 kDa
Na/K-ATPase
110 kDa
Membrane
Cytoplasm
Membrane
Cytoplasm
Membrane
Cytoplasm

Figure 5P

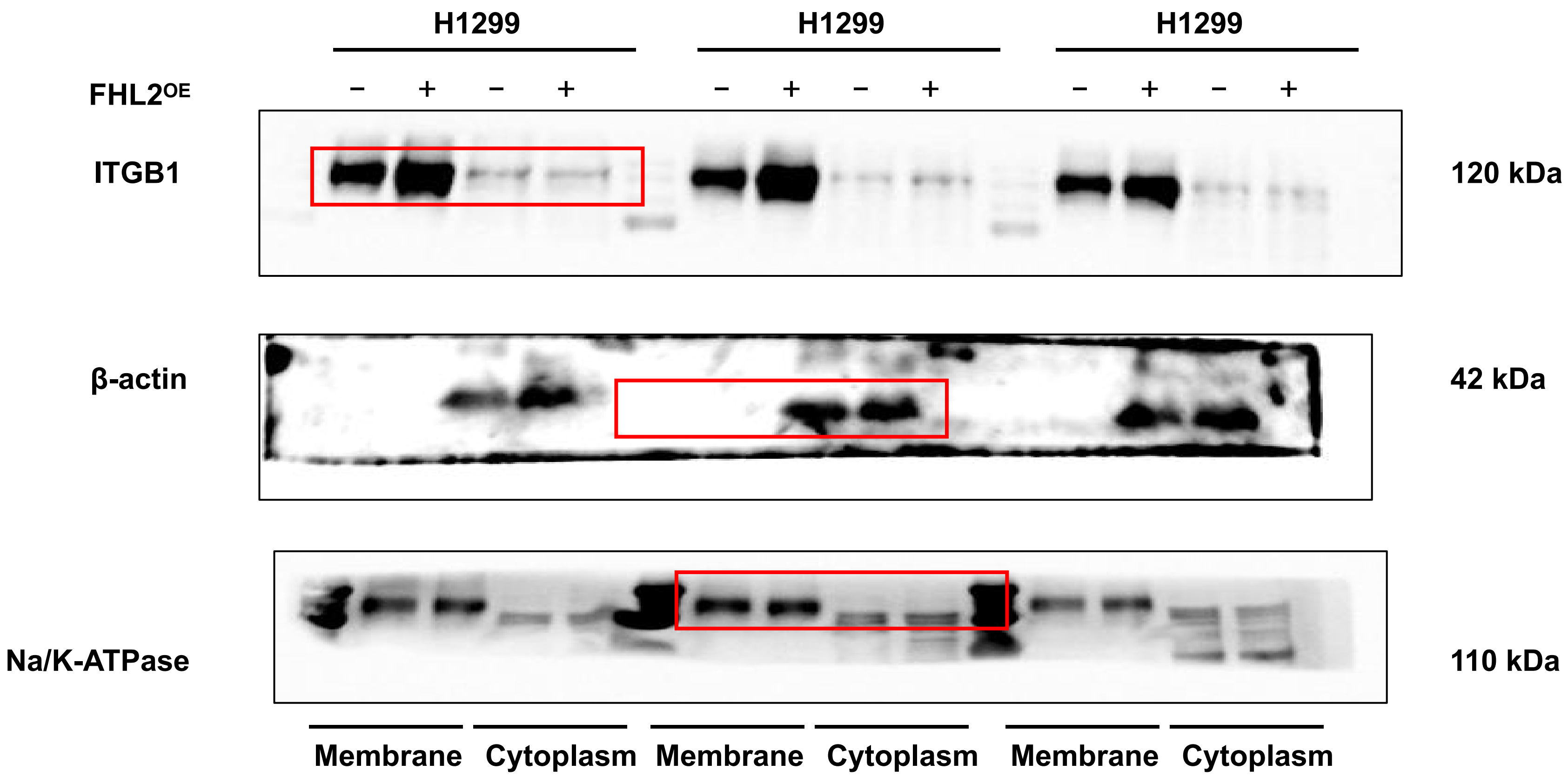

H1299
H1299
H1299
FHL2OE
– + – + – + – + – + – +
ITGB1
120 kDa
β-actin
42 kDa
Na/K-ATPase
110 kDa
Membrane
Cytoplasm
Membrane
Cytoplasm
Membrane
Cytoplasm

Figure 6A

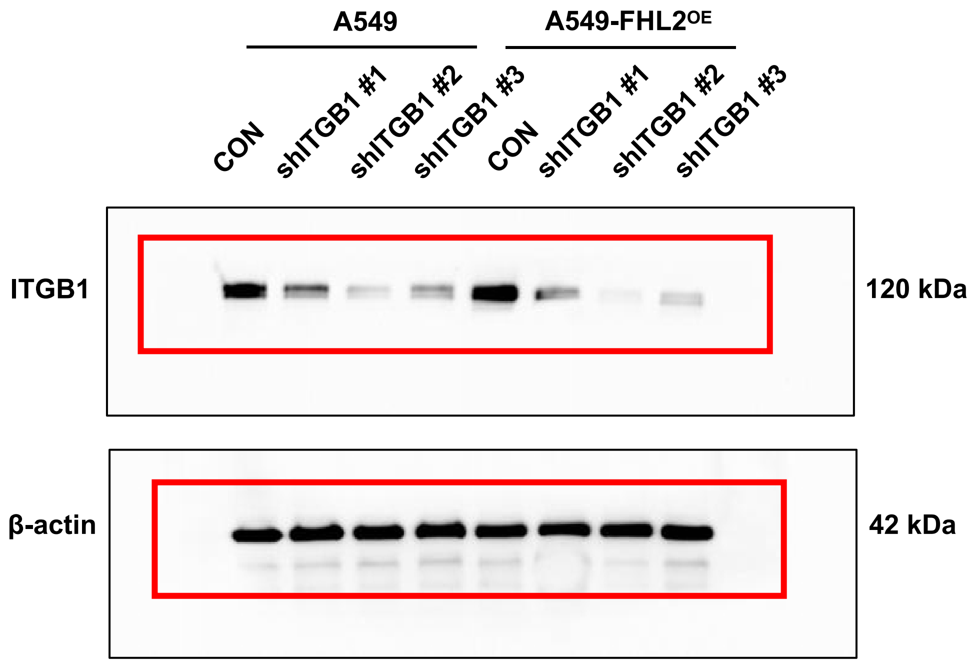

Figure 6A

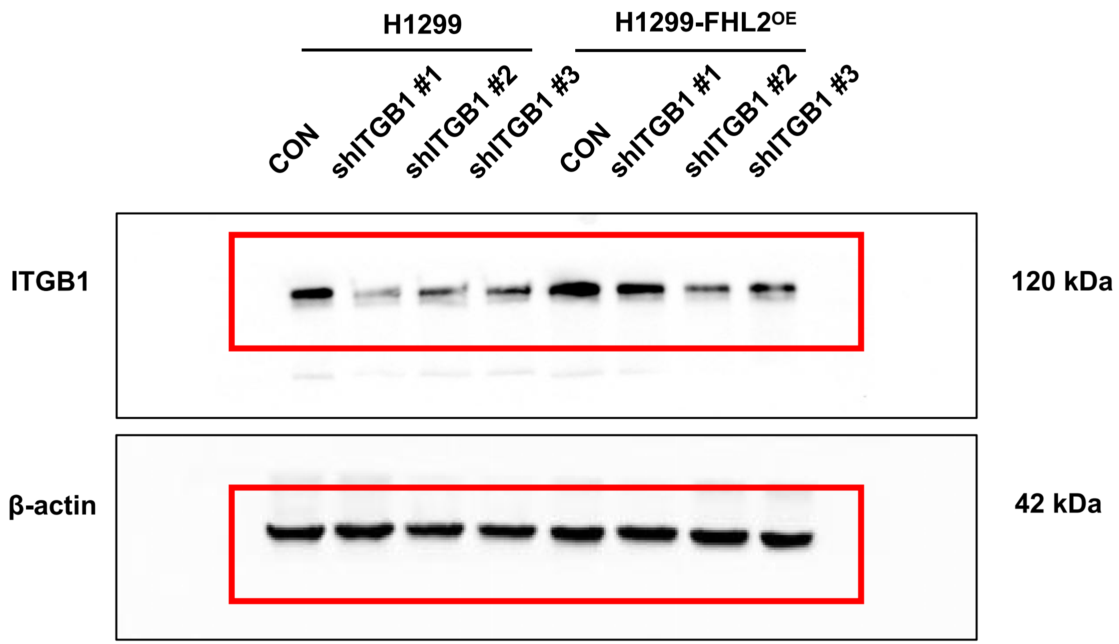

Figure 6E

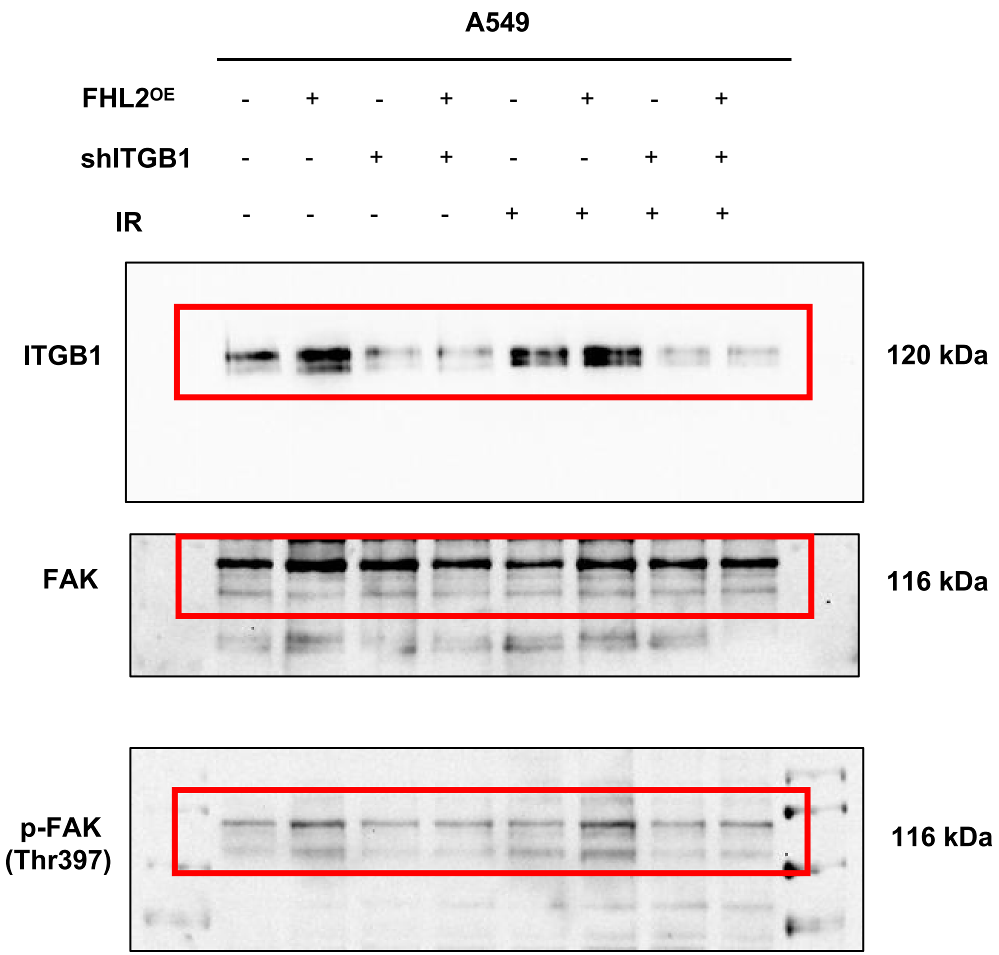
A549
FHL2OE - + - + - + - +
shITGB1 - - + + - - + +
IR - - - - + + + +
ITGB1
120 kDa
FAK
116 kDa
p-FAK
(Thr397)
116 kDa

Figure 6E

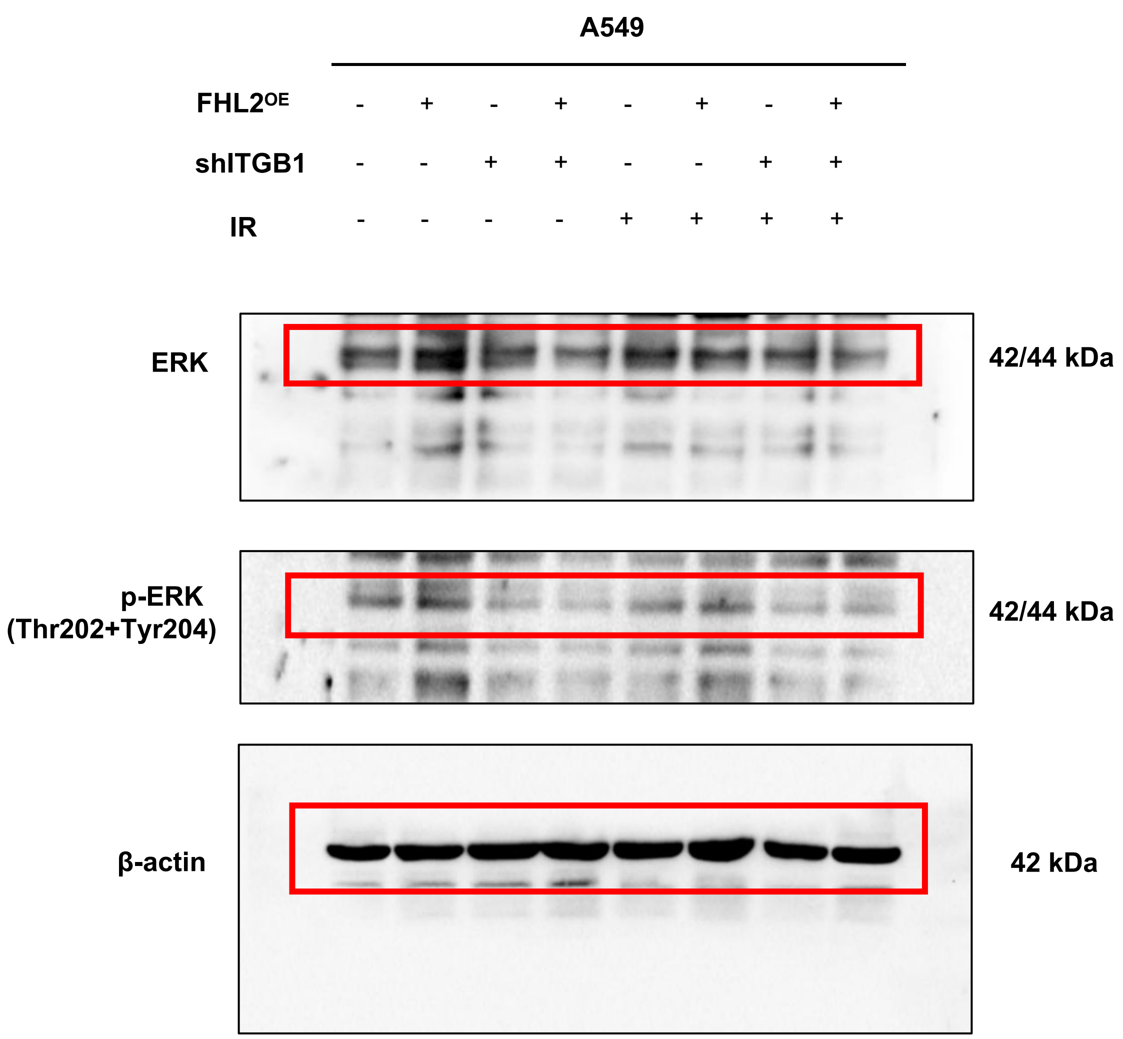
A549
FHL2OE - + - + - + - +
shITGB1 - - + + - - + +
IR - - - - + + + +
ERK
42/44 kDa
p-ERK
(Thr202+Tyr204)
42/44 kDa
β-actin
42 kDa

Figure 6F

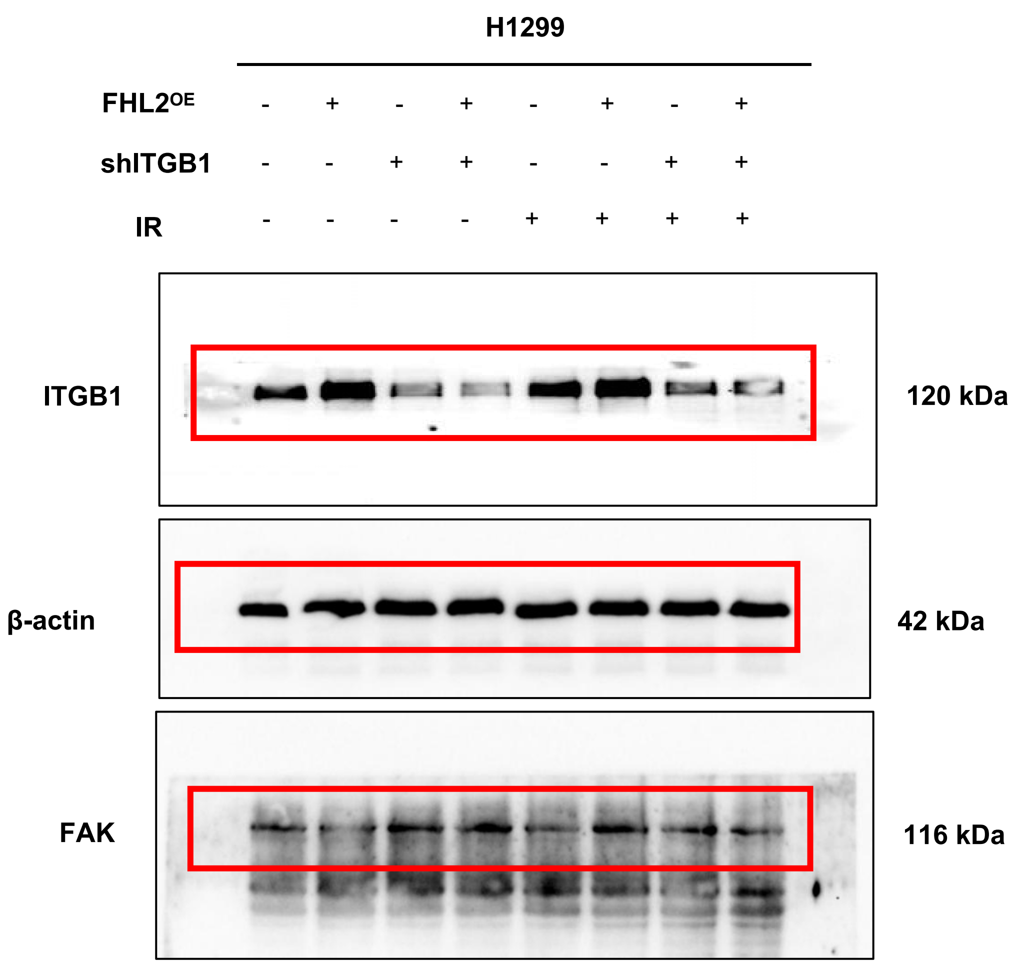
H1299
FHL2OE - + - + - + - +
shITGB1 - - + + - - + +
IR - - - - + + + +
ITGB1
120 kDa
β-actin
42 kDa
FAK
116 kDa

Figure 6F

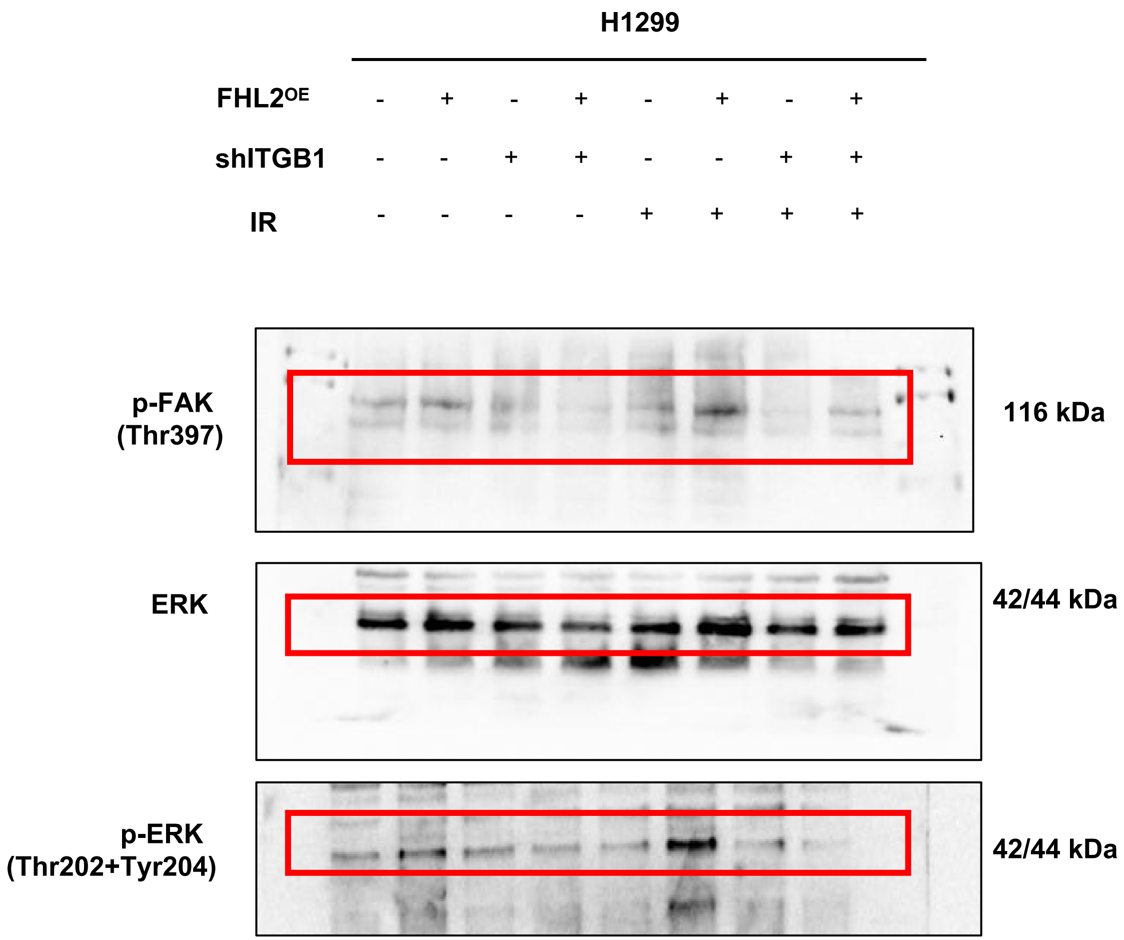

H1299
FHL2OE - + - + - + - +
shITGB1 - - + + - - + +
IR - - - - + + + +
p-FAK (Thr397)
116 kDa
ERK
42/44 kDa
p-ERK (Thr202+Tyr204)
42/44 kDa

Supplementary Figure 1B

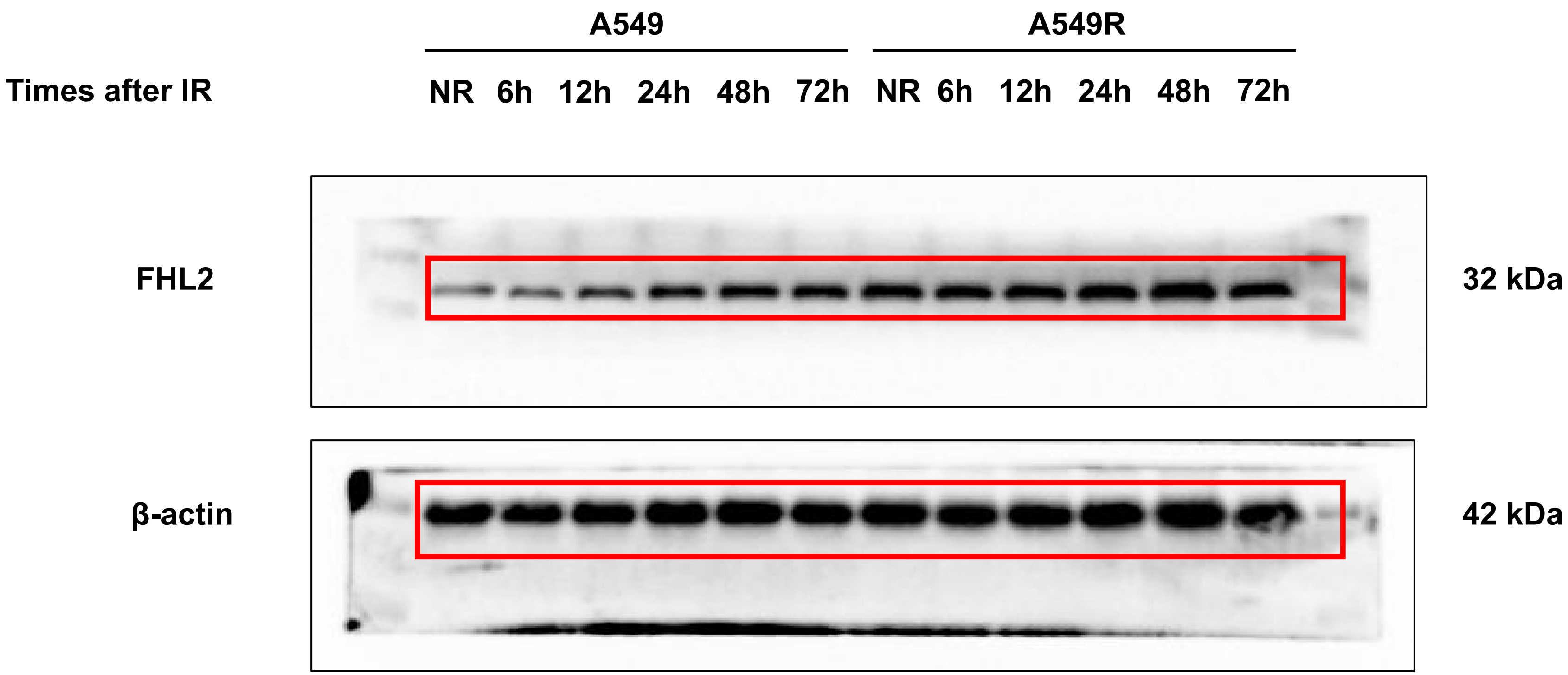

Supplementary Figure 1B

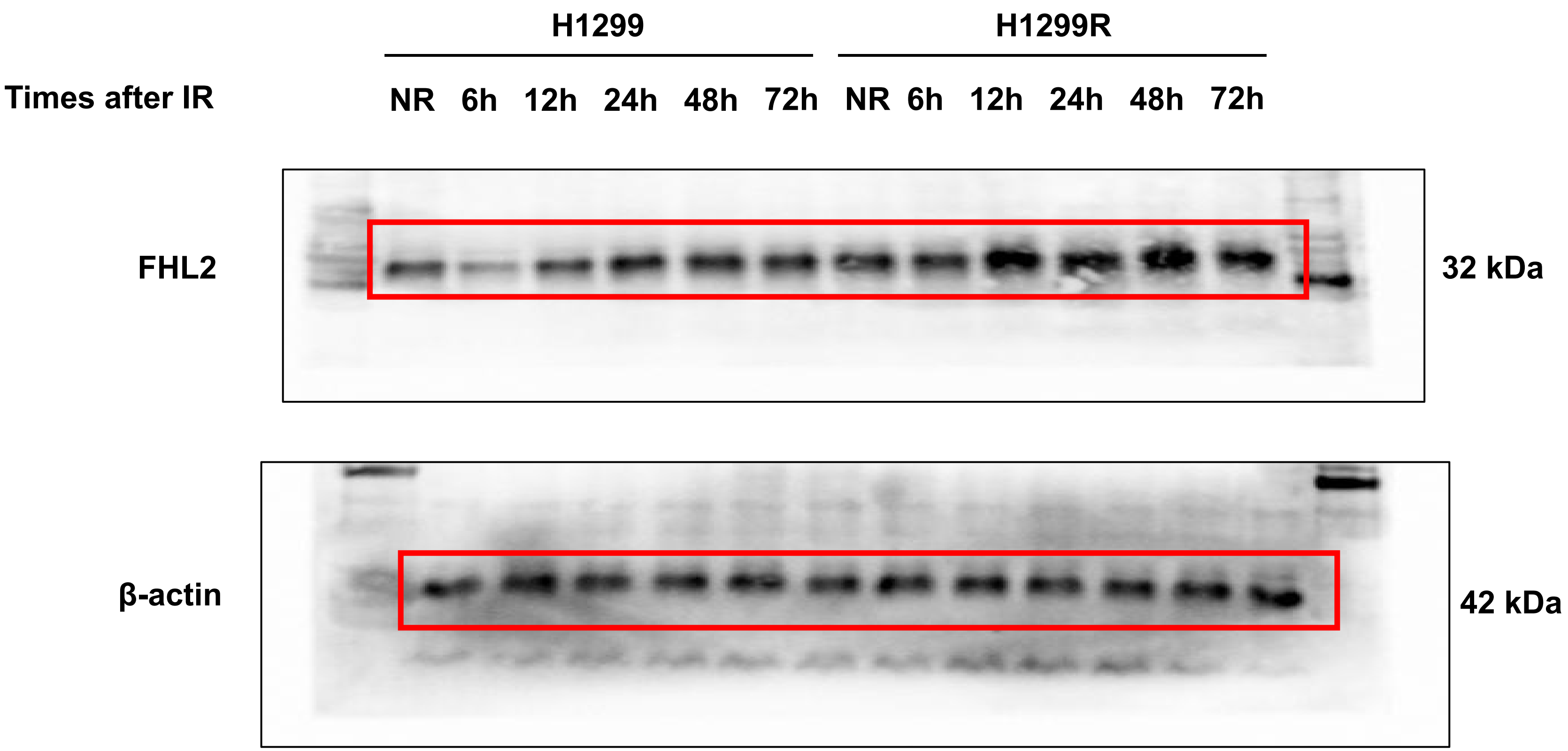

Supplement: Supplementary file 3 — Original Western Blots data [file 41420_2025_2757_MOESM3_ESM.pdf]
